# Ultra-short pulse propagation model for multi-core fibers based on local modes

**DOI:** 10.1038/s41598-017-16691-w

**Published:** 2017-11-28

**Authors:** Andrés Macho Ortiz, Carlos García-Meca, Francisco Javier Fraile-Peláez, Frederic Cortés-Juan, Roberto Llorente Sáez

**Affiliations:** 1 Nanophotonics Technology Centre, Universitat Politècnica de València, Camino de Vera s/n, 46022 Valencia, Spain; 2 Department Teoría de la Señal y Comunicaciones, Universidad de Vigo E.I. Telecomunicación, Campus Universitario, E 36202 Vigo (Pontevedra), Spain

## Abstract

Multi-core fibers (MCFs) have sparked a new paradigm in optical communications and open new possibilities and applications in experimental physics and other fields of science, such as biological and medical imaging. In many of these cases, ultra-short pulse propagation is revealed as a key factor that enables us to exploit the full potential of this technology. Unfortunately, the propagation of such pulses in real MCFs has not yet been modelled considering polarization effects or typical random medium perturbations, which usually give rise to both longitudinal and temporal birefringent effects. Using the concept of local modes, we develop here an accurate ultra-short pulse propagation model that rigorously accounts for these phenomena in single-mode MCFs. Based on this theory, we demonstrate analytically and numerically the intermodal dispersion between different LP_01_ polarized core modes induced by these random perturbations when propagating femtosecond pulses in the linear and nonlinear fiber regimes. The ever-decreasing core-to-core distance significantly enhances the intermodal dispersion induced by these birefringent effects, which can become the major physical impairment in the single-mode regime. To demonstrate the power of our model, we give explicit strategies to reduce the impact of this optical impairment by increasing the MCF perturbations.

## Introduction

In order to overcome the Shannon capacity of optical networks based on single-core single-mode fibers (SMFs), there has been an extensive research on space-division multiplexing (SDM) employing single-core multi-mode fibers (MMFs) and multi-core fibers (MCFs)^1–4^. In particular, single-mode multi-core fibers (SM-MCFs) allow us to increase the channel capacity limit of SMFs by exploiting six signal dimensions (time, wavelength, amplitude, phase, polarization and space) through spatial multi-dimensional modulation formats with a reduced digital signal processing at the receiver^5–8^. Interestingly, single-core fibers have also been used as an experimental platform for testing different phenomena related to diverse branches of physics, such as fluid dynamics, quantum mechanics, general relativity and condensed matter physics, as well as to develop applications in other fields^9–16^. Along this line, MCFs are potential laboratories that could extend the possibilities offered by single-core fibers. As an example, disordered MCFs exhibiting transverse Anderson localization have been reported as systems with potential applications in biological and medical imaging^15^.

In many of these scenarios, ultra-short optical pulses play a key factor to exploit the full potential of MCF media. In optical communications, for instance, ultra-short pulses allow us to increase the bit rate to deal with current data traffic demand and have been proposed for different applications such as supercontinuum light generation and optical combs suitable for wavelength-division multiplexed (WDM) systems^3,17–20^. In experimental physics, ultra-short optical pulses propagating in the nonlinear fiber regime have been employed to investigate important physical phenomena such as fiber-optical analogues of Hawking radiation or rogue waves on deep water via the analysis of the nonlinear Schrödinger equation^13,21^. In this way, MCFs may offer a physical platform to investigate the collision between the nonlinear solutions of these systems from a set of coupled nonlinear Schrödinger equations.

For the above reasons, it is important to have available a precise theoretical model encompassing all aspects of ultra-short pulse propagation in MCFs. In the picosecond regime, where higher-order dispersive and nonlinear effects can be neglected, the Manakov equations have been extended to MCFs and MMFs to analyse the nonlinear propagation of optical pulses wider than 1 ps by including polarization effects and the random longitudinal fiber perturbations, but omitting the temporal fluctuations of the medium and without any information of physical parameters such as the bending radius and the twist rate of the fiber^22,23^. Unfortunately, in the femtosecond regime, existing MCF propagation models exclude polarization effects and omit the temporal and longitudinal random perturbations of the fiber^24–32^. Since such perturbations modify the birefringence properties of the medium and the propagation constant of the core modes^33–35^, they should be considered in real deployed MCF systems or in experimental physics studies using this kind of optical waveguides.

In order to include these realistic fiber conditions in the mathematical description of the propagation of femtosecond optical pulses through a SM-MCF, we present here a theoretical model based on the concept of local modes, in which the aforementioned fiber perturbations and polarization effects are incorporated from the beginning in the Maxwell equations. As demonstrated analytically and numerically, the intermodal dispersion induced by these random perturbations between different fundamental polarized core modes LP_01,*mi*_ (where *m* indicates the core and *i* the polarization axis) can become the major physical impairment in the single-mode regime of the fiber when propagating ultra-short optical pulses. In this scenario, the intermodal dispersion, referred to in this work as the mode-coupling dispersion (MCD), is induced in the femtosecond regime not only by the mismatching between the propagation constants of the polarized core modes, but also by the frequency dependence of their mode overlapping. Remarkably, our results indicate that the random nature of the MCD, involving both dispersive effects and emerging from the fiber birrefringence fluctuations, should be considered for future MCF designs, digital signal processing (DSP) techniques and optical soliton transmissions in advanced SDM systems using MCFs^7^. In addition, it is worth mentioning that this model is general and can also be applied to SMF media. In the following, we first describe the proposed model in general terms, and subsequently discuss the impact of the MCD, indicating different strategies to reduce its effects via the use of fiber perturbations.

## Results

Let us consider a real SM-MCF as a nonlinear, anisotropous and temporal dispersive medium comprising longitudinal and temporal birefringent effects. Longitudinal birefringence perturbations are induced via the photo-elastic effect by macrobending, microbending and fiber twisting^35,36^. Furthermore, temporal birefringence perturbations are induced by external environmental factors, such as temperature variations and floor vibrations inducing temporal changes in the MCF structure. In order to describe theoretically ultra-short pulse propagation in real SM-MCFs considering these random perturbations, we employ the concept of local modes^35^.

**Figure 1:**
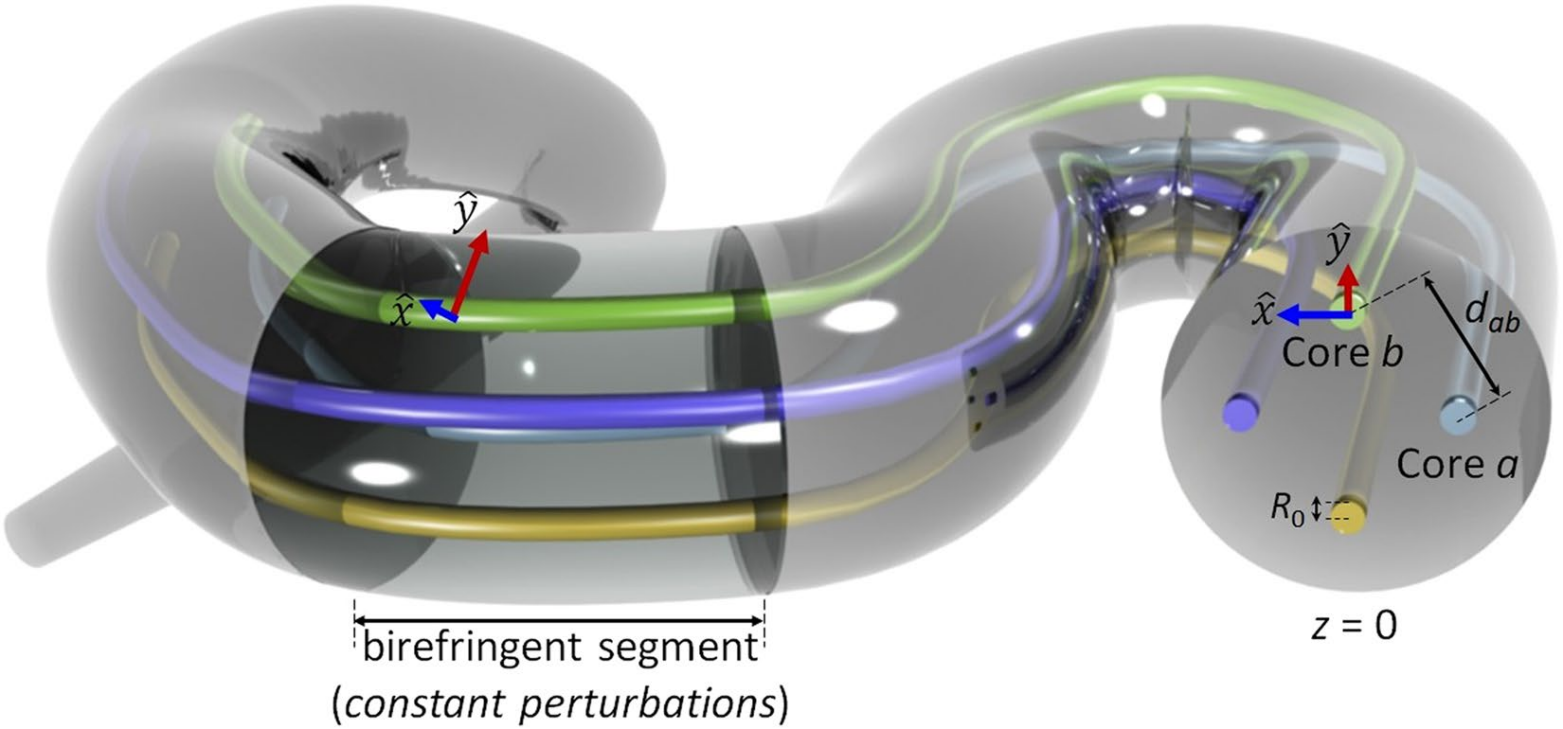
Multi-core fiber scenario of the proposed ultra-short pulse propagation model. Each core *m* propagates two polarized core modes (PCMs) LP_01,*mx*_ and LP_01,*my*_ through a series of birefringent segments, in which the longitudinal and time-varying transversal functions and phase constants of the PCMs are invariant. The PCMs of each birefringent segment define the fiber local modes. Only the cores *a* and *b* have been considered for the numerical calculations.

A local mode can be considered as an eigenfunction in a short core segment in which the perturbations of the ideal phase constant and the transversal function the LP_01_ mode are approximately constant in each polarization axis. Hence, each core can be modelled as a series of birefringent segments supporting local modes, in each of which the longitudinal and temporal MCF perturbations are approximately invariant but can fluctuate between adjacent segments (see Fig. 1). In this way, in contrast with previous works^22–32^, the fiber perturbations can be included from the beginning in the Maxwell equations. In ref.^35^, the coupled local-mode theory (CLMT) recently developed accounts for the MCF birefringence with a rigorous formalism, but considering monochromatic electromagnetic fields and omitting additional nonlinear effects such as the intrapulse stimulated Raman scattering. Consequently, the initial assumptions of the CLMT will be revisited here to develop a unified theory describing ultra-short pulse propagation in real MCFs.

### Multi-core fiber local modes

In contrast to the non-dispersive model of the CLMT^35^, we now assume non-monochromatic electric fields. Again, we consider both orthogonal polarizations in each core and a single optical carrier *ω*_0_. In order to simplify the mathematical analysis, let us describe the real wave function of the global electric field strength in the MCF structure using slowly-varying amplitude functions as in SMFs^18,37–40^:

1 $$\pmb{\mathscr{E}}({\boldsymbol{r}},t)\simeq {\rm{R}}{\rm{e}}\{\sum _{i=x,y}{E}_{i,{\omega }_{0}}({\boldsymbol{r}},t)\exp (j{\omega }_{0}t)\hat{{u}_{i}}\},$$

where *E*_*i*,*ω*0_ is the slowly-varying complex amplitude of the electric field strength in the *i* = *x*, *y* polarization axis. It should be noted that the slowly-varying amplitude approximation performed in equation (1) allows us to decouple the rapid temporal oscillation of the optical carrier from the slow temporal evolution of the complex amplitudes of the optical pulses. Therefore, the herein proposed model is valid if and only if the Maxwell equations are approximately satisfied when using equation (1). However, this assumption is not fulfilled if the pulse is too narrow, namely around the order of the period of the optical carrier or shorter. In such a case, the decomposition performed in equation (1) is no longer useful and the concept of the complex amplitude itself becomes unclear^40^. In our case, the optical carrier lies in the third transmission window (around 193.1 THz), which sets the limits of applicability of the ansatz given by equation (1) to pulses wider than ~10 fs (with a pulse bandwidth narrower than ~100 THz). In additional MCF applications which require the use of a different optical band, the validity of equation (1) can be easily tested by verifying that the pulse bandwidth is much lower than *ω*_0_/2π. Moreover, considering that the intrachannel pulse-to-pulse interactions are the predominant nonlinear effects for optical pulses shorter than ~50 ps in single-carrier and WDM transmissions^41–43^, we have assumed a single optical carrier. Consequently, the herein presented model will allow us to describe the propagation of pulses with a temporal width between 10 fs and 50 ps, not only in SM-MCF single-carrier transmissions, but also in WDM systems using these optical fibers. Specifically, in SM-MCF WDM transmissions, the derived coupled local-mode equations (see below) should be numerically solved for each optical carrier of the WDM system.

The complex amplitude *E*_*i*,*ω*0_ is the key term of the proposed model, as it will encode the MCF perturbations and the optical pulses. Using the perturbation theory^44^, *E*_*i*,*ω*0_ can be expressed in each polarization axis *i* = *x*, *y* of a *N*-core MCF as a function of the polarized core modes (PCMs) “*mi”* (with *m* = 1, …, *N*), where *mi* refers to the LP_01,*mi*_ mode associated with core *m* alone (i.e., in the absence of the other cores). In addition, each PCM *mi* can be understood as a series of local modes distributed along the different birefringent segments of core *m*. All in all, the complex amplitude *E*_*i*,*ω*0_ can be expressed as:

2 $$\begin{array}{ccc}{E}_{i,{\omega }_{0}}({\boldsymbol{r}},t) & \simeq  & \sum _{m=1}^{N}{E}_{mi,{\omega }_{0}}({\boldsymbol{r}},t)\,\\  & = & \sum _{m=1}^{N}\frac{1}{2\pi }\int {\mathop{{\rm{A}}}\limits^{ \sim }}_{mi}(z,\omega -{\omega }_{0};t){F}_{mi}(x,y,\omega ;z,t)\exp [-j{{\rm{\Phi }}}_{mi}(z,\omega ;t)]\exp [j(\omega -{\omega }_{0})t]{\rm{d}}\omega ,\end{array}$$

where *E*_*mi*,*ω*0_ is the complex amplitude of the electric field of the PCM *mi* associated with core *m* alone; ${\tilde{{\rm{A}}}}_{mi}$ is the Fourier transform of the complex envelope of the optical pulses in baseband (with Ω = *ω* − *ω*_0_), which includes the temporal birefringence fluctuations; *F*_*mi*_ is the transversal eigenfunction of the PCM *mi*; and Ф_*mi*_ is the complex phase function of the PCM *mi* modelling optical attenuation and the MCF longitudinal and temporal random perturbations:

3 $${{\rm{\Phi }}}_{mi}(z,\omega ;t)\,:={\varphi }_{mi}(z,\omega ;t)-j\frac{1}{2}\alpha (\omega )z={\beta }_{mi}(\omega )z+{\int }_{0}^{z}{\beta }_{mi}^{({\rm{B}}+{\rm{S}})}(\xi ,\omega ;t){\rm{d}}\xi -j\frac{1}{2}\alpha (\omega )z,$$

where *α* is the power attenuation coefficient of the MCF modelling optical absorption due to Rayleigh scattering (assumed to be similar in each PCM); and *ϕ*_*mi*_ is the real phase function including: (i) the ideal phase constant *β*_*mi*_, and (ii) the phase perturbation ${\beta }_{mi}^{({\rm{B}}+{\rm{S}})}$ induced by macrobending (B) and additional longitudinal and temporal fiber structure fluctuations (S).

Note that equation (2) differs substantially from the ansatz assumed in the original version of the CLMT^35^. Specifically, equation (2) assumes non-monochromatic electric fields, which allows us to describe higher-order coupling, as well as dispersive and nonlinear effects. In addition, the following considerations on the above equations are in order:

- The longitudinal and temporal MCF perturbations define the birefringent segments and the local modes in each PCM *mi*. These longitudinal and temporal changes are assumed to be slowly-varying in comparison with the spatial and temporal duration of the complex envelope. Note that these fiber perturbations are modelled in the ${\tilde{{\rm{A}}}}_{mi}$, *F*_*mi*_ and Φ_*mi*_ functions. Considering that the longitudinal and temporal MCF perturbations modify the ideal phase constant *β*_*mi*_(*ω*), thus, *F*_*mi*_ and ${\tilde{{\rm{A}}}}_{mi}$ should also be assumed to be both longitudinal and temporal dependent in order to satisfy the Maxwell equations. That is, the fiber perturbations influence not only Φ_*mi*_, but also ${\tilde{{\rm{A}}}}_{mi}$ and *F*_*mi*_. The semicolon symbol is used to denote explicitly longitudinal and temporal changes induced by these MCF perturbations (to the right of the semicolon).
- The aforementioned perturbations are included in equation (2) without approximating Ф_*mi*_(*z*, *ω*; *t*) to Ф_*mi*_(*z*, *ω*_0_; *t*). In this way, we can describe accurately the frequency dependence of the phase-mismatching between local modes including the fiber birefringence. This flexibility will allow us to investigate the behaviour of the MCD in real SM-MCFs and the reduction of this optical impairment via the use of MCF perturbations.
- When operating in the nonlinear regime, and for optical pulses shorter than 200 fs, the nuclei motion induced by the vibration of the electronic structure of silica atoms must be included in the constitutive relation between the electric field strength and the nonlinear polarization^38,45^. For optical frequencies well below the electronic transitions, the electronic contribution to the nonlinear polarization can be considered instantaneous. However, since protons and neutrons are considerably heavier than electrons, the nuclei motions have resonant frequencies much lower than the electronic transitions and, consequently, they should be retained in the constitutive relation. In particular, Raman scattering is a well-known effect arising from the nuclear contribution to the nonlinear polarization. In our model, the isotropic and anisotropic Raman response is modelled by the *h* and *u* functions, respectively (see below). In the Supplementary Information we provide a detailed description of the isotropic and anisotropic response of the nonlinear polarization with the electric field strength including both electronic and nuclei motions.

### Coupled local-mode equations for ultra-short optical pulses

Inserting equations (1)–(3) in the Maxwell equations, the complex envelopes are found to satisfy the following relation:

4 $$\begin{array}{c}\begin{array}{ccc}j({{\rm{\partial }}}_{z}+{\hat{{\rm{D}}}}_{ax}^{({\rm{e}}{\rm{q}})}+\frac{1}{2}\hat{\alpha }){{\mathscr{A}}}_{ax}(z,t) & = & {\hat{{\rm{M}}}}_{ax,ay}^{({\rm{e}}{\rm{q}})}{{\mathscr{A}}}_{ay}(z,t)+\sum _{m=b}^{N}{\hat{{\rm{K}}}}_{ax,mx}^{({\rm{e}}{\rm{q}})}{{\mathscr{A}}}_{mx}(z,t)\\  &  & +{\hat{{\rm{q}}}}_{ax}^{({\rm{I}})}({|{{\mathscr{A}}}_{ax}(z,t)|}^{2}{{\mathscr{A}}}_{ax}(z,t))+\frac{2}{3}{\hat{{\rm{g}}}}_{ax,ay}^{({\rm{I}})}({|{{\mathscr{A}}}_{ay}(z,t)|}^{2}{{\mathscr{A}}}_{ax}(z,t))\\  &  & +\frac{1}{3}\exp (-j2{\rm{\Delta }}{\varphi }_{ay,ax}^{(0)}(z;t)){\hat{{\rm{g}}}}_{ax,ay}^{({\rm{I}})}({{\mathscr{A}}}_{ax}^{\ast }(z,t){{\mathscr{A}}}_{ay}^{2}(z,t))\\  &  & +{\hat{{\rm{q}}}}_{ax}^{({\rm{R}})}[(f(t)\ast {|{{\mathscr{A}}}_{ax}(z,t)|}^{2}){{\mathscr{A}}}_{ax}(z,t)]\\  &  & +{\hat{{\rm{g}}}}_{ax,ay}^{({\rm{R}})}[(h(t)\ast {|{{\mathscr{A}}}_{ay}(z,t)|}^{2}){{\mathscr{A}}}_{ax}(z,t)]\\  &  & +\frac{1}{2}{\hat{{\rm{g}}}}_{ax,ay}^{({\rm{R}})}\{[u(t)\ast ({{\mathscr{A}}}_{ax}(z,t){{\mathscr{A}}}_{ay}^{\ast }(z,t))]{{\mathscr{A}}}_{ay}(z,t)\}\\  &  & +\frac{1}{2}\exp (-j2{\rm{\Delta }}{\varphi }_{ay,ax}^{(0)}(z;t)){\hat{{\rm{g}}}}_{ax,ay}^{({\rm{R}})}\\  &  & \times \{[u(t)\ast ({{\mathscr{A}}}_{ax}^{\ast }(z,t){{\mathscr{A}}}_{ay}(z,t))]{{\mathscr{A}}}_{ay}(z,t)\},\end{array}\end{array}$$

where ${{\mathscr{A}}}_{mi}$ (*z*, *t*) is the complex envelope of the optical pulses in the time domain; ${\hat{{\rm{D}}}}_{ax}^{({\rm{eq}})}$ is the equivalent dispersion operator in the core *a* and *x*-polarization including the frequency dependence of the MCF perturbations in the time domain; $\hat{{\rm{\alpha }}}$ is the attenuation operator, which accounts for the frequency dependence of the attenuation coefficient in the time domain; the *h* and *u* functions describe the isotropic and anisotropic Raman response, respectively; the *f* function is defined as *f* : = *h* + *u*; the phase-mismatching term ${\rm{\Delta }}{\varphi }_{ay,ax}^{(0)}(z;t)\,:={\varphi }_{ay}(z,{\omega }_{0};t)-{\varphi }_{ax}(z,{\omega }_{0};t)$ describes the phase-mismatching between the PCMs *ax* and *ay* at *ω*_0_; ${\hat{{\rm{M}}}}_{ax,ay}^{({\rm{eq}})}$ and ${\hat{{\rm{K}}}}_{ax,mx}^{({\rm{eq}})}$ are, respectively, the equivalent intra- and inter-core mode-coupling dispersion operators between the PCMs *ax*-*ay* and *ax*-*mx*; ${\hat{{\rm{q}}}}_{ax}^{({\rm{I}})}$ and ${\hat{{\rm{g}}}}_{ax,ay}^{({\rm{I}})}$ are the nonlinear mode-coupling dispersion operators associated with the instantaneous response of the nonlinear polarization and accounting for the nonlinear mode overlapping between the PCMs *ax*-*ax* and *ax*-*ay*; and ${\hat{{\rm{q}}}}_{ax}^{({\rm{R}})}$ and ${\hat{{\rm{g}}}}_{ax,ay}^{({\rm{R}})}$ are analogous to ${\hat{{\rm{q}}}}_{ax}^{({\rm{I}})}$ and ${\hat{{\rm{g}}}}_{ax,ay}^{({\rm{I}})}$, but associated with the nonlinear polarization induced by the delay response of the nuclei motion of silica atoms (Raman effect). The theoretical model is completed by 2 *N*−1 additional coupled local-mode equations, which can be obtained just by exchanging the corresponding subindexes in equation (4). A comprehensive description of the mathematical derivation of these equations and the main parameters of the model can be found in the Supplementary Information.

The proposed coupled local-mode equation presents new interesting terms when compared with previous ultra-short pulse propagation models in MCF^24–32^. Specifically, the linear operators of equation (4) are found to be longitudinal and temporal dependent, instead of constant coupling coefficients and unperturbed propagation constants. Thanks to these linear operators, our model is able to describe accurately the linear and nonlinear propagation of each PCM and the linear and nonlinear MCD (also termed in the literature as the intermodal dispersion) between different LP_01,*mi*_ modes including the longitudinal and temporal MCF perturbations.

It is worthy to note that the MCD is induced in each birefringent segment by two different dispersive effects when propagating femtosecond optical pulses through a MCF: (i) the frequency dependence of the local mismatching between the phase functions *ϕ*_*mi*_(*z*, *ω*; *t*) of the PCMs, referred to as the phase-mismatching dispersion (PhMD); and (ii) the frequency dependence of the mode overlapping between the PCMs, modelled by the coupling coefficients and referred to as the coupling-coefficient dispersion (CCD). As an example, the PhMD between the PCMs *ax* and *mx* is given by the phase-mismatching Δ*ϕ*_*mx,ax*_(*z*, *ω*; *t*) and the CCD by the coupling coefficients ${\tilde{k}}_{ax,mx}(z,\omega ;t)$ and ${\tilde{k}}_{mx,ax}(z,\omega ;t)$, both dispersive effects modelled by the operators ${\hat{{\rm{D}}}}_{ax}^{({\rm{eq}})}$, ${\hat{{\rm{D}}}}_{mx}^{({\rm{eq}})}$, ${\hat{{\rm{K}}}}_{ax,mx}^{({\rm{eq}})}$ and ${\hat{{\rm{K}}}}_{mx,ax}^{({\rm{eq}})}$. Along this line, note that the equivalent dispersion operators ${\hat{{\rm{D}}}}_{ax}^{({\rm{eq}})}$ and ${\hat{{\rm{D}}}}_{mx}^{({\rm{eq}})}$ describe not only the linear propagation of the PCMs *ax* and *mx*, but also the exact phase-mismatching Δ*ϕ*_*mx,ax*_(*z*, *ω*; *t*) at each angular frequency *ω* at a given *z* point. Although the CCD has been previously reported considering ideal MCFs without birefringent effects, the CCD and the PhMD induced by these perturbations have been overlooked so far in the femtosecond regime^24–32^. However, the analysis of both physical impairments is essential to describe the propagation of ultra-short optical pulses in real MCFs perturbed by longitudinal and temporal birefringent effects. The first- and higher-order MCD induced by both dispersive effects will be further analysed in the next sections.

Remarkably, the MCD can be observed in a SM-MCF between the PCMs of different cores (inter-core MCD) and between the PCMs of a single core (intra-core MCD). Note that the intra-core MCD is the well-known linear and nonlinear polarization-mode dispersion (PMD). Hence, from now on we will discuss the inter-core MCD (IMCD) involving mode-coupling between the PCMs of different cores.

### Inter-core mode-coupling dispersion

Although the proposed model allows us to investigate a wide range of propagation phenomena in MCFs, our efforts are mainly focused on a deeper understanding of the IMCD induced by the fiber perturbations. In order to clarify the impact of the MCF birefringence on this physical impairment when propagating femtosecond optical pulses, we discuss the IMCD from equation (4) by omitting the optical power attenuation, the PMD (intra-core MCD) and the nonlinear effects in a first approximation. For simplicity, to facilitate the physical interpretation and gain insight into the effects of the IMCD, let us also consider only two cores *a* and *b*, a single polarization axis (along the *x* direction), and a short MCF segment in a time interval where the longitudinal and temporal fiber perturbations can be assumed to be constant. Note that these initial assumptions allow us to investigate the IMCD in a MCF comprising cores of different characteristics: heterogeneous, homogeneous, coupled, uncoupled, lowly- or highly-birefringent, trench- or hole-assisted, and with step- or gradual-index profile.

In this case, stimulating the PCM *ax* at *z* = 0, the IMCD can be modelled in each PCM and in the MCF segment by two different linear and time-invariant (LTI) systems with the following transfer functions in baseband Ω = *ω* − *ω*_0_ (see section 2 of the Supplementary Information for more details):

5a $$\begin{array}{ccccc}{H}_{ax}(z,{\rm{\Omega }})\,: & = & \frac{{\mathop{{\rm{A}}}\limits^{ \sim }}_{ax}(z,{\rm{\Omega }})}{{\mathop{{\rm{A}}}\limits^{ \sim }}_{ax}(0,{\rm{\Omega }})} & = & \exp (-\,j\frac{{\rm{\Delta }}{\beta }_{bx,ax}^{({\rm{e}}{\rm{q}})}({\rm{\Omega }}+{\omega }_{0})}{2}z)\\  &  &  &  & \times [\cos (\mathop{\eta }\limits^{ \sim }({\rm{\Omega }})z)+j\frac{{\rm{\Delta }}{\beta }_{bx,ax}^{({\rm{e}}{\rm{q}})}({\rm{\Omega }}+{\omega }_{0})}{2\mathop{\eta }\limits^{ \sim }({\rm{\Omega }})}\,\sin (\mathop{\eta }\limits^{ \sim }({\rm{\Omega }})z)];\end{array}$$

5b $${H}_{bx}(z,{\rm{\Omega }})\,:=\frac{{\tilde{{\rm{A}}}}_{bx}(z,{\rm{\Omega }})}{{\tilde{{\rm{A}}}}_{ax}(0,{\rm{\Omega }})}=-j\frac{{\tilde{k}}_{bx,ax}({\rm{\Omega }}+{\omega }_{0})}{\tilde{\eta }({\rm{\Omega }})}\exp (+j\frac{{\rm{\Delta }}{\beta }_{bx,ax}^{({\rm{eq}})}({\rm{\Omega }}+{\omega }_{0})}{2}z)\sin (\tilde{\eta }({\rm{\Omega }})z),$$

where *H*_*ax*_ and *H*_*bx*_ are the transfer functions of the two LTI systems describing the IMCD effects in the PCMs *ax* and *bx*, respectively; ${\rm{\Delta }}{\beta }_{bx,ax}^{({\rm{eq}})}$ is the mismatching of the equivalent phase constants between the PCMs *ax*-*bx* in the MCF segment, including the fiber perturbations ${\rm{\Delta }}{\beta }_{bx,ax}^{({\rm{B}}+{\rm{S}})}$ and the intrinsic phase-mismatching ${\rm{\Delta }}{\beta }_{bx,ax}$ when considering heterogeneous cores; and $\tilde{\eta }(z,{\rm{\Omega }})$ is the complex function defined as:

6 $$\tilde{\eta }({\rm{\Omega }})\,:={[{\tilde{k}}_{ax,bx}({\rm{\Omega }}+{\omega }_{0}){\tilde{k}}_{bx,ax}({\rm{\Omega }}+{\omega }_{0})+ \sim {({\rm{\Delta }}{\beta }_{bx,ax}^{({\rm{eq}})}({\rm{\Omega }}+{\omega }_{0}))}^{2}/4]}^{1/2},$$

with ${\tilde{k}}_{ax,bx}$ and ${\tilde{k}}_{bx,ax}$ the coupling coefficients between the PCMs *ax* and *bx*. Furthermore, performing the first-order Taylor series approximation ${\rm{\Delta }}{\beta }_{bx,ax}^{({\rm{eq}})}\approx {\rm{\Delta }}{\beta }_{bx,ax}^{({\rm{eq}})(0)}+{\rm{\Delta }}{\beta }_{bx,ax}^{({\rm{eq}})(1)}{\rm{\Omega }}$ and $\tilde{\eta }({\rm{\Omega }})\approx {\tilde{\eta }}^{(0)}+{\tilde{\eta }}^{(1)}{\rm{\Omega }}$ (with ${\rm{\Delta }}{\beta }_{bx,ax}^{({\rm{e}}{\rm{q}})(r)}:={{\rm{d}}}^{r}{\rm{\Delta }}{\beta }_{bx,ax}^{({\rm{e}}{\rm{q}})}({\rm{\Omega }}=0)/{{\rm{d}}{\rm{\Omega }}}^{k}$ and ${\mathop{\eta }\limits^{ \sim }}^{(r)}:={{\rm{d}}}^{r}\mathop{\eta }\limits^{ \sim }({\rm{\Omega }}=0)/{{\rm{d}}{\rm{\Omega }}}^{k}$), we can directly infer the main implications of the IMCD from equations (5):

- For ideal homogeneous cores without MCF perturbations $({\rm{\Delta }}{\beta }_{bx,ax}^{({\rm{eq}})}=0)$, it is straightforward to demonstrate that the impulse response of both LTI systems is proportional to the Dirac delta functions ${\delta }(t\pm {\tilde{\eta }}^{(1)}z)$, as detailed in Section 2.1 of the Supplementary Information. Therefore, the pulse splitting induced by the first-order CCD and predicted by Chiang^24^ can be observed in both cores for a MCF length *L* satisfying the condition $L > {T}_{{\rm{P}}}/2{\tilde{\eta }}^{(1)}$, where *T*_P_ is the temporal pulse width. In ref.^32^, the heterogeneous case was analysed following a similar approach to the original work of Chiang^24^, that is, considering the two-core fiber as an ideal optical coupler and thus omitting the realistic perturbations of the medium.
- The power of our model reveals itself when considering real homogeneous and heterogeneous MCFs with longitudinal and temporal birefringent effects inducing a significant local phase-mismatching ${\rm{\Delta }}{\beta }_{bx,ax}^{({\rm{eq}})}$. In this case, the LTI systems introduce an additional group delay (with opposite sign in cores *a* and *b*) induced by the exponential terms of equations (5), and therefore, the impulse response is found to be proportional to $\delta (t\pm {\tilde{\eta }}^{(1)}z\pm {\rm{\Delta }}{\beta }_{bx,ax}^{({\rm{eq}})(1)}z)$. Note that this effect is produced by the first-order PhMD, modelled by the term ${\rm{\Delta }}{\beta }_{bx,ax}^{({\rm{eq}})(1)}$, which accounts for the equivalent differential group delay between the PCMs *ax* and *bx*. Consequently, the pulse splitting and the group delay induced by the first-order CCD and the first-order PhMD inherit the possible random nature of ${\tilde{\eta }}^{(1)}$ and ${\rm{\Delta }}{\beta }_{bx,ax}^{({\rm{eq}})(1)}$ along the MCF length (arising from the stochastic perturbations of the medium). In addition, the IMCD can also vary in time following the temporal fluctuations of the MCF perturbations that modify the value of ${\tilde{\eta }}^{(1)}$ and ${\rm{\Delta }}{\beta }_{bx,ax}^{({\rm{eq}})(1)}$.
- The increment of the MCF perturbations given by $|{\rm{\Delta }}{\beta }_{bx,ax}^{({\rm{eq}})}|$ increases the group delay in the non-excited core *b*, but reduces the group delay in the excited core *a*. Note that the transfer function *H*_*ax*_ can be approximated to *H*_*ax*_ ≈ 1 if the absolute value of the equivalent phase-mismatching $|{\rm{\Delta }}{\beta }_{bx,ax}^{({\rm{eq}})}|$ is much higher than the coupling coefficients, as can be deduced from equations (5a) and (6). Consequently, the MCF perturbations can be used as a potential strategy of birefringence management to reduce the impact of the IMCD on the MCF transmission.
- As mentioned before in the theoretical description of equation (4), and also inferred from equations (5), the CCD and the PhMD are induced by the frequency dependence of the coupling coefficients and the local phase-mismatching ${\rm{\Delta }}{\beta }_{bx,ax}^{({\rm{eq}})}$, respectively. Thus, the second- and higher-order dispersive effects of the IMCD will introduce an additional chirp in the optical pulses, modifying their complex envelopes. In particular, although the higher-order PhMD is difficult to observe between orthogonal PCMs of a given core with similar dispersive parameters (also termed in the literature as the higher-order PMD), the higher-order PhMD becomes an important issue when PCMs of different cores are involved. In the same way, higher-order dispersive effects of the CCD will also modify the complex envelopes of the optical pulses.
- In spite of the fact that equations (5) are only an approximate solution of the coupled local-mode equations in the linear regime of the fiber, these expressions also allow us to infer a fundamental behaviour of the IMCD in the nonlinear regime. The Kerr effect will increase $|{\rm{\Delta }}{\beta }_{bx,ax}^{({\rm{eq}})}|$ reducing the mode-coupling between both PCMs, in line with the behaviour of the nonlinear inter-core crosstalk experimentally observed in ref.^34^. Nonetheless, in general, equation (4) must be solved numerically to perform a complete analysis of the IMCD effects in the nonlinear regime. As we will see below, additional propagating effects will appear on the optical pulses induced by the MCF nonlinearities, which can only be observed when solving numerically the coupled local-mode equations.

These points are verified through numerical calculations of equation (4) in next section. As we will see, many interesting IMCD effects related to MCF perturbations that could not be addressed with previous femtosecond pulse propagation models^24–32^ emerge when using the proposed theory.

### Numerical calculations

**Figure 2:**
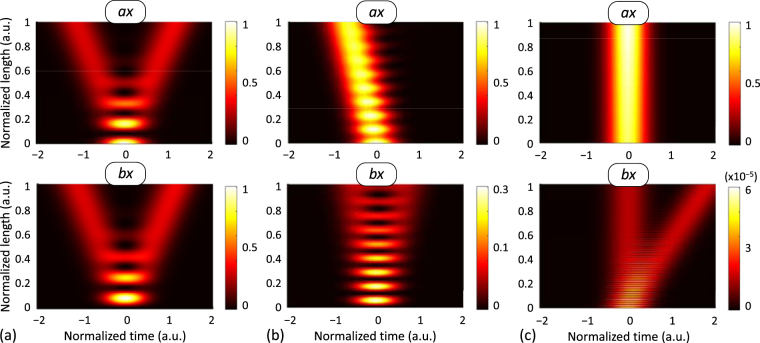
First-order IMCD with constant bending conditions. Simulation results of a 350-fs Gaussian optical pulse propagating through a 40-m homogeneous 2-core MCF (cores *a* and *b*) with three different constant bending radius: (**a**) *R*_B_ = ∞, (**b**) *R*_B_ = 10 cm and (**c**) *R*_B_ = 1 cm. (Colorbar: normalized intensity).

**Figure 3:**
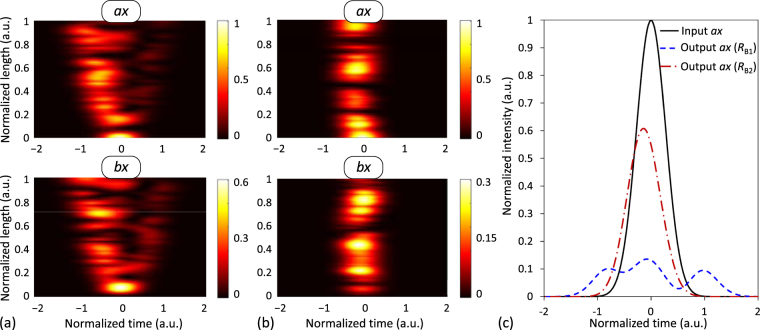
First-order IMCD with random bending conditions. Simulation results of a 250-fs Gaussian optical pulse propagating through a homogeneous 2-core MCF comprising 50 birefringent segments with random bending conditions. Two different normal distributions were considered: (**a**) *R*_B1_ = N(*μ* = 100, *σ* ^2^ = 40) cm and (**b**) *R*_B2_ = N(*μ* = 10, *σ* ^2^ = 2) cm. (**c**) Pulse dispersion comparison at the MCF output for the PCM *ax*. (Colorbar: normalized intensity).

**Figure 4:**
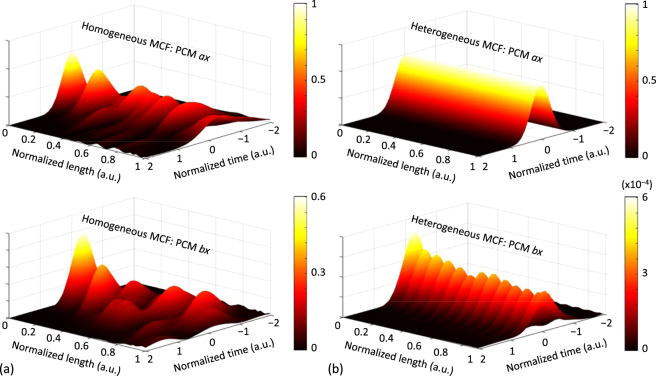
Higher-order IMCD in real homogeneous and heterogeneous MCFs. 200-fs Gaussian optical pulse propagated along a 40-m 2-core MCF in the PCMs *ax* and *bx* considering higher-order PhMD effects. (**a**) Homogeneous MCF. (**b**) Heterogeneous MCF with index difference Δ*n* = *n*_*a*_ − *n*_*b*_ = 0.002. (Colorbar: normalized intensity).

**Figure 5:**
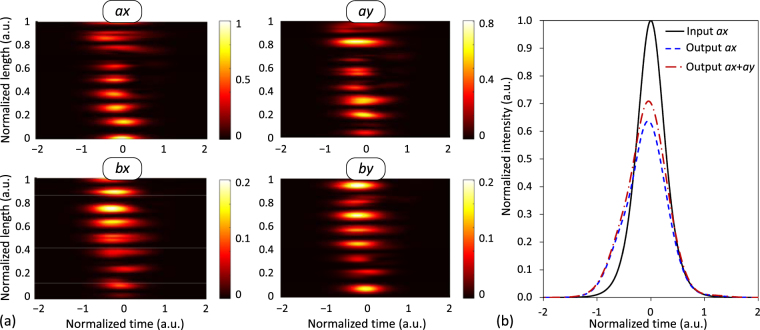
IMCD impact on optical solitons. (**a**) 600-fs fundamental soliton propagated along a 40-m 2-core dispersion-shifted homogeneous MCF in the PCMs *ax, ay, bx, by* considering first- and second-order IMCD effects. (**b**) Pulse shape comparison at the output of the core *a*. (Colorbar: normalized intensity).

**Figure 6:**
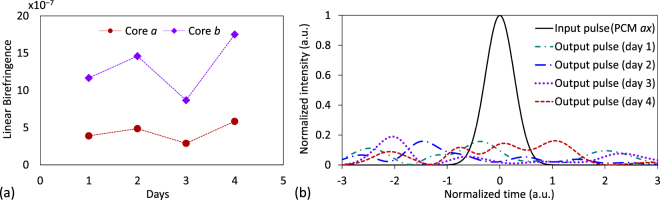
Time-varying IMCD. (**a**) Temporal evolution of the intrinsic linear birefringence assumed in cores *a* and *b* for the numerical simulations. (**b**) Corresponding optical pulse calculated in the PCM *ax* at the MCF output each simulated day.

In all the analysed cases, we considered a MCF comprising a fiber length of *L* = 40 m and two cores *a* and *b* distributed in a square lattice with a core-to-core distance *d*_*ab*_ = 26 μm and a core radius *R*_0_ = 4 μm, as depicted in Fig. 1. The wavelength of the optical carrier *λ*_0_ was selected to be in the third transmission window with *λ*_0_ = 1550 nm. The peak power of the optical pulses was taken to be 0 dBm to analyse the IMCD effects in the linear regime (Figs 2, 3, 4 and 6) and 40.7 dBm to investigate the impact of the medium perturbations on a fundamental soliton (Fig. 5). The time variable was normalized using the group delay of the PCM *ax* as a reference with *t*_N_ = (*t* − *β*_*ax*_^(1)^ *z*)/*T*_P_, where *T*_P_ is defined in this work as the full-width at 1/2*e* (~18%) of the peak power. In order to investigate the main effects of the IMCD, different fiber parameters are considered in each numerical example. The specific parameters of each simulation are detailed in Tables S1 and S2 of the Supplementary Information.

As a first simple example, we considered a homogeneous MCF with constant bending conditions. The material refractive index of the cores *a* and *b* and the cladding was selected to be *n*_*a*_ = *n*_*b*_ = 1.452 and *n*_cl_ = 1.444, respectively. Figure 2 shows the simulation results of the coupled local-mode equations when a 350-fs Gaussian optical pulse is launched into the PCM *ax* at *z* = 0. In this example, the linear birefringence is induced via the photo-elastic effect by three different constant bending radius *R*_B_ = ∞, 10 cm and 1 cm, depicted in Fig. 2(a–c), respectively. Moreover, the chromatic dispersion [also known as group-velocity dispersion (GVD)] and the PMD (induced by the intrinsic random fiber birefringence) were omitted to isolate the effects of the first-order IMCD. In this way, the pulse is only propagated by the PCMs *ax* and *bx*.

Figure 2(a) depicts the ideal homogeneous MCF, where the pulse splitting previously observed by Chiang^24^ appears induced by the first-order CCD (see Supplementary Information for more details). Moreover, it can be observed from Fig. 2(b) and (c) that the lower the bending radius, the higher the phase-mismatching induced between the PCMs is. Therefore, an additional group delay appears in the optical pulse induced by the first-order PhMD, with opposite sign in the PCMs *ax* and *bx*. Specifically, note that the group delay increases in core *b* when reducing the bending radius as a direct consequence of the impulse response *h*_*bx*_(*t*) [see equations (5)], which is proportional to the Dirac delta function $\delta (t\pm {\tilde{\eta }}^{(1)}z+{\rm{\Delta }}{\beta }_{bx,ax}^{({\rm{eq}})(1)}z)$ (with ${\rm{\Delta }}{\beta }_{bx,ax}^{({\rm{eq}})(1)}$ < 0 in this case). In contrast, the group delay decreases in core *a* when the bending radius is reduced [Fig. 2(c)]. As was pointed out previously, *H*_*ax*_ tends to 1 as $|{\rm{\Delta }}{\beta }_{bx,ax}^{({\rm{eq}})}|$ increases. Furthermore, the pulse splitting is reduced in both cores because of the reduction of the mode-coupling induced by the fiber bending. This shows that MCF longitudinal perturbations (low values of the bending radius in this case) can be used to reduce the effect of the IMCD along the MCF propagation.

Another interesting potential effect of the first-order IMCD is related to the random birefringence that arises from a randomly-varying fiber bending radius. In this case, the effect of the first-order PhMD along with the CCD can also be observed when considering a high number of MCF birefringent segments where the bending radius fluctuates with a Normal distribution between adjacent segments. We simulate the MCF of the first example considering a 250-fs Gaussian optical pulse and 50 birefringent segments with two different random distributions of the bending radius (*R*_B_): *R*_B1_ = N(*μ* = 100, *σ* ^2^ = 40) cm and *R*_B2_ = N(*μ* = 10, *σ* ^2^ = 2) cm, where N is the normal distribution function. The numerical results are shown in Fig. 3(a) and (b) for each bending radius distribution, respectively.

As can be seen from Fig. 3(a), the group delay and the pulse splitting present a random evolution in each core due to the stochastic nature of the MCF perturbations inducing a random differential group delay ${\rm{\Delta }}{\beta }_{bx,ax}^{({\rm{eq}})(1)}$ between the PCMs *ax* and *bx*, in line with our previous theoretical predictions. However, if the average value of the bending radius is reduced, the phase-mismatching between the core modes increases (see Section 4 of the Supplementary Information), and therefore, the pulse dispersion induced by the first-order PhMD decreases, as can be seen from Fig. 3(b) and (c). The comparison of the pulse dispersion at the MCF output in the PCM *ax* is shown in Fig. 3(c) for each bending radius distribution, verifying that the IMCD is reduced with the second bending radius *R*_B2_. It should be noted that, for higher fiber distances, although the intrinsic random linear birefringence of the MCF may increase the pulse distortion (via the PMD), small index differences induced in each core by the fiber fabrication process could reduce the inter-core crosstalk levels^46^ and the IMCD effects. On the other hand, although the circular birefringence has been omitted in these simulations to isolate the effects of the IMCD, in the Supplementary Information we include additional simulations in which both linear and circular random birefringent effects are considered. As can be noted from Fig. S3, the circular birefringence only induces power exchange between orthogonal polarizations. Remarkably, we cannot observe an additional pulse distortion in this case taking into account that the PMD can be neglected in both cores when the intrinsic linear birefringence is omitted in the numerical simulations.

In the next example, we compare the effects of the IMCD induced by the CCD and higher-order effects of the PhMD in homogeneous and heterogeneous MCFs. As detailed in the previous section, higher-order dispersive effects of the PhMD appear when considering a non-vanishing Δ*β*_*bx,ax*_^(*r*)^ with *r* ≥ 2. To investigate the additional chirp induced by the higher-order PhMD, a 200-fs Gaussian optical pulse was simulated in the same homogeneous MCF as in the previous examples, but with Δ*β*_*bx,ax*_^(1)^ = 0.28 ps/km, Δ*β*_*bx,ax*_^(2)^ = 0.2 ps^2^/km and Δ*β*_*bx,ax*_^(3)^ = 0 ps^3^/km (which are typical values induced by manufacturing imperfections^47,48^). A second heterogeneous MCF with Δ*n* = *n*_*a*_ − *n*_*b*_ = 0.002, Δ*β*_*bx,ax*_^(1)^ = 6.5 ps/km, Δ*β*_*bx,ax*_^(2)^ = 1 ps^2^/km and Δ*β*_*bx,ax*_^(3)^ = 0.1 ps^3^/km was also simulated (dispersive parameters which can be found in a heterogeneous MCF desing^49^). In both cases, the bending radius was assumed to vary randomly along 50 birefringent segments as a Normal distribution of *R*_B_ = N(*μ* = 100, *σ* ^2^ = 40) cm. In order to illustrate the higher-order PhMD effects, the GVD is compensated in both cores along the MCF propagation using the dispersive parameters of a given PCM as a reference, in this case the PCM *ax* (the specific dispersive parameters and additional details of this simulation can be found in Table S2 of the Supplementary Information).

The simulation results are shown in Fig. 4, where we can observe the additional chirp induced by the second- and third-order PhMD (Δ*β*_*bx,ax*_^(2)^ and Δ*β*_*bx,ax*_^(3)^), which increases the temporal width of the pulse complex envelope in the PCMs *ax* and *bx*. Although Δ*β*_*bx,ax*_^(2)^ and Δ*β*_*bx,ax*_^(3)^ are lower in the homogeneous MCF [Fig. 4(a)] than in the heterogeneous MCF [Fig. 4(b)], the pulse distortion induced by the higher-order IMCD is significantly higher in the former case. In the heterogeneous case, the second-order PhMD effects are reduced due to the increment of the intrinsic phase-mismatching Δ*β*_*bx,ax*_(*ω*_0_) between the PCMs *ax* and *bx*. These results allow us to conclude that, in the femtosecond regime, the GVD compensation can be performed at the DSP using the same digital filter for each PCM when heterogeneous cores and short MCF distances are involved (*L* ≤ *L*_PhMD_ = *T*_P_^2^/|Δ*β*_*bx,ax*_^(2)^|, see below). Nonetheless, in homogeneous MCFs, while the criterion *L* ≤ *L*_PhMD_ is fulfilled, Δ*β*_*bx,ax*_(*ω*_0_) = 0. Hence, the second-order PhMD induces a higher pulse distortion and the GVD compensation must be performed using a different digital filter per core, with the specific dispersive parameters of each one. In the case of which *L* ≪ *L*_PhMD_ in homogeneous MCFs, the GVD compensation can also be performed using the same digital filter for each core. Moreover, due to their low inter-core crosstalk levels, disordered MCFs exhibiting transverse Anderson localization^14,15^ could also be proposed as a means to reduce the impact of the IMCD on some applications. In particular, disordered MCFs could be of extreme utility to improve the image quality in lensless endoscopy^50^. In the Supplementary Information we also analyse in Figs S4 and S5 the higher-order effects of the IMCD when including mode-coupling between orthogonal polarizations induced by the circular fiber birefringence. In both cases, we can observe a higher pulse distortion than in Fig. 4 when including the circular birefringence along with the PMD and the second-order PhMD.

For completeness, the IMCD effects are also investigated in the nonlinear fiber regime along with the PMD (intra-core MCD). Remarkably, the impact of such perturbations on a bright soliton is analysed. A 600-fs fundamental soliton (~350 fs full width at half maximum) was launched into the PCM *ax* of a dispersion-shifted homogeneous 2-core MCF with *n*_*a*_ = *n*_*b*_ = 1.452, *n*_*cl*_ = 1.444, and usual first- and second-order GVD coefficients of *β* ^(2)^ = −1 ps^2^/km and *β* ^(3)^ = 0.1 ps^2^/km, respectively. The peak power (*P*_0_) required to support the fundamental soliton is found to be 40.7 dBm considering a nonlinear refractive index of *n*_NL_ = 2.6·10^−20^ *m* ^2^/W at 1550 nm. The fundamental soliton condition was numerically tested by omitting: the core *b*, the fiber birefringent effects, *β* ^(3)^, the self-steepening (induced by the frequency dependence of ${\hat{{\rm{q}}}}_{ax}^{({\rm{I}})}$), and the intrapulse Raman scattering inducing frequency shift in optical pulses shorter than 1 ps [Raman-induced frequency shift (RIFS)]^18^.

Now, in order to simulate realistic MCF conditions, we include the core *b*, higher-order dispersive and nonlinear effects, and assume Δ*β*_*bx,ax*_^(1)^ = 0.2 ps/km and Δ*β*_*bx,ax*_^(2)^ = 0.1 ps^2^/km induced by manufacturing imperfections (similar values for the *y*-polarization). In this case, we also include the intrinsic linear birefringence of the medium along with the linear and circular birefringence induced by the fiber bending and twisting conditions. We consider 50 birefringent segments along the MCF length, where the linear and circular birefringence fluctuate between adjacent segments. The circular birefringence is induced by a random twist rate *f*_T_ given by the Normal distribution *f*_T_ = N(*μ* = 0.1, *σ* ^2^ = 0.01) turns/m. The linear birefringence is induced by: (i) the random bending conditions with *R*_B_ = N(*μ* = 100, *σ* ^2^ = 40) cm; and (ii) the intrinsic linear birefringence of each core, fixed to 2·10^−7^ in both cores *a* and *b*.

According to Fig. 5, we can observe that the soliton condition is broken along the MCF propagation. As discussed later, the second-order PhMD becomes one of the major physical impairment in coupled-core MCFs with a near-zero Δ*β*_*bx,ax*_^(2)^ parameter. Therefore, in the first propagation meters, the additional chirp induced by the second-order PhMD along with the first-order CCD increases the pulse width and reduces the peak power. As a result of the peak power reduction, the pulse width is increased along the MCF length and the soliton peak is shifted from its original position due to the first-order PhMD and the second-order GVD (induced by *β* ^(3)^). In this case, note that the effects of the RIFS and the self-steepening are difficult to observe with *T*_P_ = 600 fs, *L* = 40 m, *β* ^(2)^ = −1 ps^2^/km, and *P*_0_ ≈ 40 dBm. Nevertheless, in optical pulses of few femtoseconds and in MCFs with a higher *β* ^(2)^ coefficient, the soliton distortion will be increased not only by the IMCD and the second-order GVD, but also by the RIFS and the self-steepening nonlinear effects.

So far, we have evaluated the longitudinal birefringent effects of the MCF, but omitting the temporal perturbations of the medium. However, as was indicated above, the IMCD can also fluctuate in time due to the temporal fluctuation of the MCF birefringence modifying the value of the phase function *ϕ*_*mi*_(*z*,*ω*;*t*) in each PCM *mi*. Therefore, the random group delay induced by the first-order PhMD in each MCF segment may present a time-varying evolution.

To verify this statement, we perform a numerical simulation considering a time-varying intrinsic linear birefringence of the optical medium. Specifically, we simulate the homogeneous 2-core MCF of Fig. 4(a) but assuming a constant bending radius of *R*_B_ = 100 cm and varying the intrinsic linear birefringence of each core over a 4-day period. The intrinsic linear birefringence was assumed to vary from day to day following a normal distribution with different average value in each core, but with a similar temporal evolution, in line with the experimental work reported in ref.^35^ [see Fig. 6(a)]. Nonetheless, note that faster temporal changes of the linear birefringence can also be considered in each core in line with ref.^51^. In any case, our previous discussion and the coupled local-mode equations are also found to be valid for faster time-varying birefringent conditions if these MCF fluctuations are approximately constant in time intervals of the order of *T*_P_, as indicated above. Figure 6(b) shows the temporal dispersion of a 150-fs Gaussian optical pulse obtained each day at the MCF output for the PCM *ax*. As can be seen, the group delay and the pulse shape presents random fluctuations in different days as a direct consequence of the temporal random group delay induced by the first-order PhMD and CCD in each MCF segment. From these results, it is clear that the time-varying effects of the IMCD should be taken into account to compensate for this physical impairment using DSP techniques in future SDM optical systems.

Finally, once we know in general terms the effects of the IMCD in ultra-short optical pulses, we investigate the fiber length scales over which the dispersive effects of the IMCD should be considered in the pulse propagation phenomena when comparing this physical impairment with the first-order GVD. To this end, we compare the GVD, CCD and PhMD lengths considering a MCF without random perturbations, given by the expressions for the PCMs *ax* and *bx* (see Supplementary Information for more details):

7 $${L}_{{\rm{GVD}}}\,:={T}_{{\rm{P}}}^{2}/|{\beta }_{ax}^{(2)}|;\quad {L}_{{\rm{CCD}}}\,:={T}_{{\rm{P}}}/2|{\tilde{k}}_{ax,bx}^{(1)}|;\quad {L}_{{\rm{PhMD}}}\,:={T}_{{\rm{P}}}^{2}/|{\rm{\Delta }}{\beta }_{bx,ax}^{(2)}|.$$

**Figure 7:**
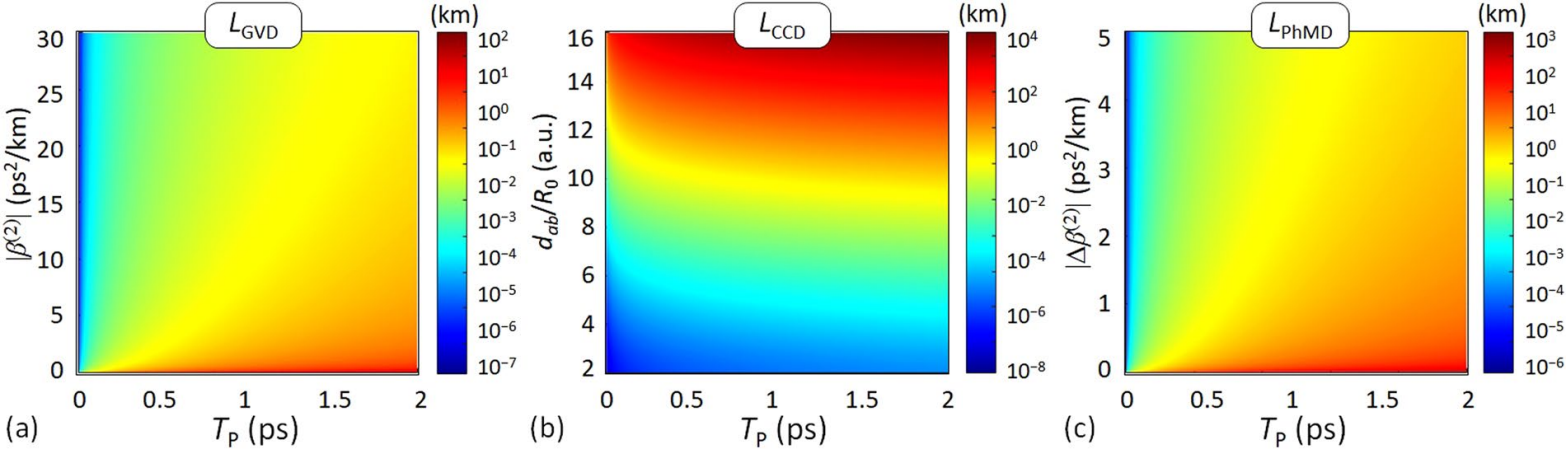
Comparison of the dispersion lengths. (**a**) Group-velocity dispersion (GVD) length, (**b**) coupling-coefficient dispersion (CCD) length, and (**c**) phase-mismatching dispersion (PhMD) length.

Figure 7 depicts the comparison of the GVD, CCD and PhMD dispersion lengths. As can be seen, the first-order GVD is expected to become the major physical impairment in MCFs where the mode-coupling effects are significantly reduced. This scenario should be considered in homogeneous uncoupled-core MCFs, i.e., with *β*_*ax*_^(2)^ ≈ *β*_*bx*_^(2)^ and *d*_*ab*_/*R*_0_ > 7, or in heterogeneous MCFs with inter-core crosstalk levels lower than −30 dB [see Fig. 4(b)]. Specific examples of these fibers can be found in refs^48,52^. On the other hand, the IMCD becomes one of the major pulse distortion effects in MCFs operating in the strong coupling regime (*d*_*ab*_/*R*_0_ < 7). In particular, femtosecond pulses propagating in coupled-core MCFs^53,54^ will be highly degraded by this optical impairment. In this scenario, the IMCD induced by the first-order CCD becomes the predominant impairment in coupled-core MCFs with homogeneous and low dispersive cores, i.e., with *β*_*ax*_^(2)^ ≈ *β*_*bx*_^(2)^ < 10 ps^2^/km. Nevertheless, the first-order GVD along with the IMCD induced by the second-order PhMD will be the predominant physical impairments in coupled-core MCFs with Δ*β*_*bx,ax*_^(2)^ ≠ 0, especially when these fibers comprise homogeneous but non-identical cores with a near-zero Δ*β*_*bx,ax*_^(2)^ parameter.

## Discussion

**Figure 8:**
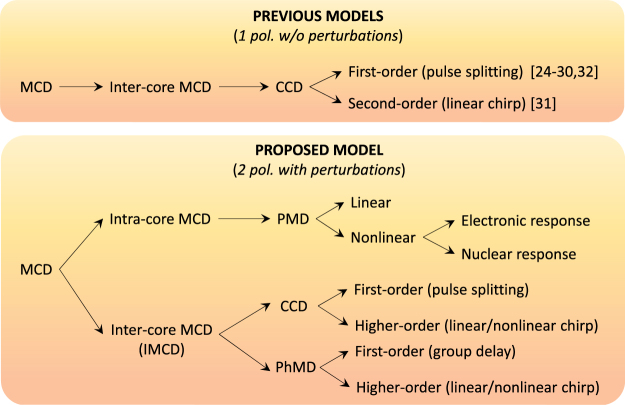
Schematic comparison of the MCD effects which can be analysed with the proposed model and previous works of femtosecond pulse propagation in MCFs. Considering a single polarization, ideal homogeneous cores and omitting the fiber perturbations, only the CCD can be modelled. However, including both orthogonal polarizations and the perturbations of the optical medium, the linear and nonlinear PMD along with the IMCD (CCD + PhMD) can be investigated.

We have presented a general theory modelling the propagation of ultra-short optical pulses in real SM-MCFs perturbed by random longitudinal and temporal birefringent effects. The rigorous formalism here reported including the longitudinal and temporal fiber birefringent perturbations allows us to describe many interesting effects that could not be addressed with previous ultra-short pulse propagation models in the femtosecond regime^24–32^. Figure 8 shows a schematic comparison of our model with previous works.

As can be seen, previous ultra-short pulse propagation models^24–32^ consider a single polarization, ideal cores and omit the fiber birefringent perturbations. Therefore, these works can only describe the frequency dependence of the ideal coupling coefficients, the CCD. More specifically, the first- and second-order CCD inducing pulse splitting and linear chirp was investigated from these models.

Nevertheless, new dispersive effects induced by the mode-coupling among the fundamental core modes LP_01_ emerge when including two polarizations and the fiber perturbations in the Maxwell equations using the concept of local modes. Numerical calculations based on the developed theory reveal the existence of intermodal dispersion, referred to as the MCD in this work, induced by the random perturbations of the optical medium when operating in the linear and nonlinear fiber regimes. Specifically, in the femtosecond regime, the inter-core MCD involves the frequency dependence of the mode overlapping and the phase-mismatching between the fiber local modes, dispersive effects referred to as the CCD and the PhMD, respectively. The CCD is completed in our model by including the fiber perturbations and higher-order dispersive effects inducing pulse splitting and nonlinear chirp. The PhMD, overlooked so far in previous models^24–32^, emerges from: (i) the longitudinal and temporal fiber perturbations inducing a random group delay (first-order PhMD), and (ii) the intrinsic phase-mismatching between local modes inducing a deterministic group delay and a linear and nonlinear chirp (second- and higher-order PhMD). It is worth noting that the PhMD is analogous to the PMD observed in optical transmissions using SMFs. Nonetheless, although the second-order PMD is difficult to observe in SMF transmissions, the second-order PhMD is expected to become the predominant physical impairment (along with the first-order GVD) in coupled-core MCFs comprising homogeneous but non-identical cores with a near-zero Δ*β*_*bx,ax*_^(2)^ parameter [see Fig. 4(a) and Fig. 7(c)]. In contrast, the CCD will be the predominant IMCD effect in coupled-core MCFs with low dispersive homogeneous cores [see Fig. 7(b)].

The potential implications of these effects evidenced by our model should be considered in the future to enhance the performance of MCFs for communication applications and to improve our understanding and control over MCF-based experimental physics. As an important example, the core-to-core distance of the MCFs employed in optical networks is being progressively reduced to increase the number of cores in a single cladding^53–56^. A reduced core-to-core distance will increase the IMCD and the pulse distortion, as discussed in Fig. 7. In this scenario, our results show that the increment of the MCF perturbations (increasing the average value of the linear birefringence via the photo-elastic effect and mismatching the phase constant of the local modes) is an effective birefringence management strategy to reduce the impact of the IMCD on the MCF. Remarkably, our results also indicate that the second-order PhMD induces a significant higher pulse distortion in real homogeneous MCFs, with not identical but similar dispersive parameters, than in heterogeneous MCFs, with a higher value of Δ*β* ^(2)^ between adjacent cores. As a result, in heterogeneous MCFs, the digital compensation of the GVD in femtosecond optical pulses can be performed at the DSP using the same digital filter for each core when short propagation distances are involved (*L* ≤ *L*_PhMD_).

Furthermore, note that in contrast with previous SMF and MCF models^18,22–35,39^, our theory also includes the nonlinear PMD and the nonlinear IMCD that arises from the isotropous and anisotropous response of the nonlinear polarization considering both electronic and nuclei motion. In particular, our results show that the linear and nonlinear IMCD induced by the external fiber perturbations and manufacturing imperfections should also be taken into account when propagating femtosecond optical solitons in MCFs. More specifically, the random distortion and the pulse chirping emerging from the first- and second-order IMCD break the soliton condition along the MCF propagation (see Fig. 5). Hence, the proposed model allows us to investigate the impact of the linear and nonlinear birefringence induced by the fiber perturbations and higher-order nonlinear effects on optical solitons, rogue waves and breathers^21^.

The CLMT can be applied to design MCFs comprising cores of different manufacturing characteristics: homogeneous, heterogeneous, coupled, uncoupled, lowly- or highly-birefringent, trench- or hole-assisted, step- or gradual-index. Therefore, this general theory allows us to implement novel MCF designs operating in single-mode regime with specific IMCD characteristics, which open new paths to explore in dispersion engineering and pulse shaping applications. However, additional nonlinear terms involving cross-coupling effects among the PCMs of different cores should be included for coupled-core MCFs with a core-to-core distance lower than three times the core radius (*d*_*ab*_ < 3*R*_0_), as discussed in ref.^34^. Nevertheless, in such a case, the accuracy of this model (based on the perturbation theory) may be reduced if the supermodes of the MCF do not meet the approximation performed in equation (2) when assuming that *E*_*i*,*ω*0_ ≈ ∑*E*_*mi*,*ω*0_. Moreover, while the computational time of the coupled local-mode equations may increase considerably when large MCF distances are involved, it may be reduced by inserting phase plates^23^ between birefringent segments (see Section 4 of the Supplementary information for more details).

On the other hand, in spite of the fact that we have focused our analysis on the single-mode regime of the fiber, note that the extension of equation (4) to the multi-mode regime is straightforward when including additional LP mode groups in the complex amplitude of the global electric field strength *E*_*i*,*ω*0_ given by equation (2). Inserting *E*_*i*,*ω*0_ in the Maxwell equations, the coupled local-mode equations can be extended to the multi-mode regime by performing a similar mathematical discussion as in the single-mode regime. Along these lines, it should be noted that equation (5) can also be employed to analyse the impact of the intermodal dispersion on the linear regime between higher-order LP modes of different cores by calculating the appropriate value of the coupling coefficients and the equivalent phase-mismatching.

Finally, it is worth mentioning that our model can also play an essential role in other branches of physics. As mentioned in the introduction, single-core fibers have been investigated as an experimental platform for testing diverse physical phenomena from various fields, including quantum mechanics, general relativity or condensed matter physics, among others^9–16^, based on the analogies of the fiber-optical nonlinear Schrödinger equation. In a similar way, the CLMT can be employed to elucidate the underlying wave propagation phenomena of any physical system with propagating equations of the form of the coupled nonlinear Schrödinger equations, that is, our coupled local-mode equations when higher-order coupling, dispersive and nonlinear effects are omitted. Hence, exotic physical phenomena such as superposed nonlinear waves in coherently coupled Bose-Einstein condensates^57^, interacting rogue waves^58–60^ or nonlinear ion-acoustic waves^61,62^ can be explored in MCF media expanding the possibilities of single-core fibers. In the same line, additional physical phenomena such as relativistic effects could also be analysed using MCF media. Note that an optical pulse propagating through a single-core fiber establishes a moving medium which corresponds to a space-time geometry. This gravitational approach was employed by Philbin *et al*.^13^ to demonstrate a fiber-optical analogy of the event horizon in a black hole using an ultra-short optical pulse of 70 fs. Therefore, additional gravitational phenomena could be investigated in MCFs when adjacent cores perturb the virtual space-time geometry created by an ultra-short optical pulse propagating in a given core of the fiber.

## Methods

Numerical calculations have been performed in Matlab, combining the coupled local-mode equations presented in this work with the equivalent refractive index model^35^ and the split-step Fourier method^18^. The split-step Fourier method allows us to simulate linear and nonlinear propagation employing a low computational time. According to this method, equation (4) is rewritten as:

8 $$({{\rm{\partial }}}_{z}+{\hat{{\rm{D}}}}_{ax}^{({\rm{e}}{\rm{q}})}+\frac{1}{2}\hat{\alpha }){{\mathscr{A}}}_{ax}(z,t)+j{\hat{{\rm{M}}}}_{ax,ay}^{({\rm{e}}{\rm{q}})}{{\mathscr{A}}}_{ay}(z,t)+j\sum _{m=b}^{N}{\hat{{\rm{K}}}}_{ax,mx}^{({\rm{e}}{\rm{q}})}{{\mathscr{A}}}_{mx}(z,t)={\hat{{\rm{N}}}}_{ax}^{({\rm{e}}{\rm{q}})}{{\mathscr{A}}}_{ax}(z,t),$$

where ${\hat{{\rm{N}}}}_{ax}^{({\rm{eq}})}$ is the operator modelling the nonlinear propagation of the PCM *ax*. Then, the left-hand side of equation (8), which describes the linear propagation, is simulated in the frequency domain, while the right-hand side, which accounts for the nonlinear propagation, is simulated in the time domain. A detailed description of the computational method along with the physical parameters used in the numerical simulations can be found in Section 4 of the Supplementary Information.

## Electronic supplementary material

Supplementary Information
