# Supplementary material for: Ultra-short pulse propagation model for multi-core fibers based on local modes
Source: Sci Rep. 2017 Nov 28;7:16457. doi: 10.1038/s41598-017-16691-w (PMC5705628; doi:10.1038/s41598-017-16691-w)
Supplement: Supplementary file 1 — Supplementary Information [file 41598_2017_16691_MOESM1_ESM.pdf]

Supplementary Information: Ultra-short Pulse Propagation Model for Multi-Core Fibers based on Local Modes

Andrés Macho[1,*], C. García-Meca[1], F. Javier Fraile-Peláez[2], F. Cortés-Juan[1] & Roberto Llorente[1]

[1]Nanophotonics Technology Centre, Universitat Politècnica de València, Camino de Vera s/n, 46022 Valencia, Spain
[2]Dept. Teoría de la Señal y Comunicaciones, Universidad de Vigo E.I. Telecomunicación, Campus Universitario, E 36202 Vigo (Pontevedra), Spain
*amachor@ntc.upv.es

Abstract

This supplementary information is organised as follows. First, we will describe in some more detail the derivation of the coupled local-mode equations modelling the propagation of ultra-short pulses in multi-core fiber media. Next, we will analyse the mode-coupling dispersion in the linear regime. In the third section, we will give some numerical examples that illustrate our findings. Finally, we will describe the numerical method and the main fiber parameters used in the numerical calculations.

Contents

1 Ultra-short pulse propagation model **2**
- 1.1 Multi-core fiber local modes . . . 2
- 1.2 Coupled-wave equations . . . 4
- 1.3 Coupled local-mode equations . . . 6

2 Theoretical analysis of the IMCD in ideal and real MCFs **14**
- 2.1 Ideal homogeneous MCFs . . . 14
- 2.2 Real homogeneous and heterogeneous MCFs . . . 16

3 Numerical examples **17**
- 3.1 Ideal homogeneous multi-core fiber: first- and higher-order CCD . . . 17
- 3.2 Linear and circular random birefringence perturbations: first-order IMCD . . . 19
- 3.3 Higher-order IMCD . . . 20
- 3.4 IMCD length . . . 21

4 Numerical method: local split-step Fourier method **23**

1 Ultra-short pulse propagation model

Consider a real weakly-guiding multi-core fiber (MCF) operating in the single-mode regime with longitudinal and temporal birefringence effects. The longitudinal birefringence perturbations are induced by macrobending, microbending, fiber twisting and intrinsic manufacturing imperfections. The temporal birefringence perturbations are induced by external environmental factors, such as temperature variations and floor vibrations inducing temporal changes in the bending radius and twist rate. In order to describe the propagation of ultra-short optical pulses in the linear and nonlinear fiber regime, the MCF can be modelled as a nonlinear, anisotropous and temporal dispersive medium.

Moreover, in order to include the MCF random perturbations from the Maxwell equations, we employ a similar formalism as in Ref. [1] using the concept of local modes. A local mode is defined as an eigenfunction in a short core segment in which the perturbations of the ideal phase constant and the transversal eigenfunction of the LP_{01} mode are approximately constant in both orthogonal polarizations. Hence, each core can be modelled as a series of birefringent segments and local modes where the longitudinal and temporal MCF perturbations are approximately invariant but can fluctuate between adjacent segments, as shown in Fig. 1 of the manuscript.

Now, in contrast to Ref. [1], let us assume non-monochromatic electric fields to model the dispersive nature of the MCF. We consider both orthogonal polarizations in each core and a single optical carrier with angular frequency ω_0. Thus, the real wave function of the global electric field strength of the MCF can be expressed using the slowly-varying amplitude approximation as:

$$\boldsymbol{\mathcal{E}}\left(\boldsymbol{r},t\right) \simeq \frac{1}{2}\sum_{i=x,y}\left[E_{i,\omega_0}\left(\boldsymbol{r},t\right)\exp\left(j\omega_0 t\right) + E_{i,-\omega_0}\left(\boldsymbol{r},t\right)\exp\left(-j\omega_0 t\right)\right]\hat{u}_i, \tag{1}$$

where $E_{i,\omega 0}$ is the complex amplitude of the electric field strength in the $i = x, y$ polarization axis; and $E_{i,-\omega 0}$ is the complex conjugate term. Note that the slowly-varying amplitude approximation performed in Eq. (1) allows us to decouple the rapid temporal oscillation of the optical carrier from the slow temporal evolution of the complex amplitudes [2]-[4]. In order to satisfy the Maxwell equations when using Eq. (1), we assume the optical carrier located in the third transmission window (∼193.1 THz) and optical pulses wider than 10 fs. A detailed discussion of the limits of applicability of Eq. (1) can be found in the main text.

1.1 Multi-core fiber local modes

The theoretical model we propose entails as an essential prerequisite to include the fiber perturbations from the Maxwell equations to describe ultra-short pulse propagation in MCF media. To this end, the longitudinal and temporal fluctuations of the fiber should be described by the complex amplitude E_{i,ω_0}. From the perturbation theory [5], this term can be expressed in each polarization axis $i = x, y$ of a N-core MCF as a function of the polarized core modes (PCMs) mi (with $m = 1, ..., N$), where mi refers to the $LP_{01,mi}$ mode associated with core m alone (i.e., in the absence of the other cores). In addition, each PCM mi comprises a series of local modes distributed along the different birefringent segments of the core m. All in all, the complex amplitude E_{i,ω_0} is expressed as follow:

$$\begin{aligned} E_{i,\omega_0}\left(\boldsymbol{r},t\right) &\simeq \sum_{m=1}^{N} E_{mi,\omega_0}\left(\boldsymbol{r},t\right) \\ &= \sum_{m=1}^{N}\frac{1}{2\pi}\int \widetilde{\mathtt{A}}_{mi}\left(z,\omega-\omega_0;t\right)F_{mi}\left(x,y,\omega;z,t\right)\exp\left(-j\Phi_{mi}\left(z,\omega;t\right)\right)\exp\left(j\left(\omega-\omega_0\right)t\right)\mathrm{d}\omega, \end{aligned} \tag{2}$$

where E_{mi,ω_0} is the complex amplitude of the electric field of the PCM mi considering isolated cores; $\widetilde{\mathtt{A}}_{mi}$ is the Fourier transform of the complex envelope in baseband (with $\Omega = \omega - \omega_0$) including the temporal birefringence fluctuations; F_{mi} is the transversal eigenfunction of the PCM mi; and Φ_{mi} is the complex phase function

of the PCM mi modelling optical attenuation and the MCF longitudinal and temporal random perturbations:

$$\Phi_{mi}\left(z,\omega;t\right) := \phi_{mi}\left(z,\omega;t\right) - j\frac{1}{2}\alpha\left(\omega\right)z, \tag{3}$$

where α is the power attenuation coefficient of the MCF modelling optical absorption due to Rayleigh scattering and assumed similar in each PCM; and ϕ_{mi} is the real phase function involving the longitudinal and temporal MCF perturbations in each spectral component ω:

$$\phi_{mi}\left(z,\omega;t\right) := \int_0^z \beta_{mi}^{(\mathrm{eq})}\left(\xi,\omega;t\right)\mathrm{d}\xi = \beta_{mi}\left(\omega\right)z + \int_0^z \beta_{mi}^{(\mathrm{B+S})}\left(\xi,\omega;t\right)\mathrm{d}\xi, \tag{4}$$

with $\beta_{mi}^{(\mathrm{eq})}$ the equivalent phase constant of each local mode of the PCM mi, which comprises the ideal phase constant β_{mi} and the phase perturbation $\beta_{mi}^{(\mathrm{B+S})}$ induced by macrobends (B) and additional longitudinal and temporal fiber structure fluctuations (S). In addition, it should be remarked for future mathematical discussions that (we denote $\partial_z = \partial/\partial z$):

$$\partial_z\Phi_{mi} = \beta_{mi}^{(\mathrm{eq})} - j\tfrac{1}{2}\alpha;\quad \partial_z^2\Phi_{mi} = \partial_z\beta_{mi}^{(\mathrm{eq})};\quad \left(\partial_z\Phi_{mi}\right)^2 \simeq \left(\beta_{mi}^{(\mathrm{eq})}\right)^2 - j\alpha\beta_{mi}^{(\mathrm{eq})}. \tag{5}$$

Thus, the Fourier transform ($\mathcal{F}$) of Eqs. (1) and (2) is found to be:

$$\boldsymbol{E}\left(\boldsymbol{r},\omega;t\right) = \mathcal{F}\left[\boldsymbol{\mathcal{E}}\left(\boldsymbol{r},t\right)\right] = \frac{1}{2}\sum_{i=x,y}\left[\widetilde{E}_{i,\omega_0}\left(\boldsymbol{r},\omega-\omega_0;t\right) + \widetilde{E}_{i,-\omega_0}\left(\boldsymbol{r},\omega+\omega_0;t\right)\right]\hat{u}_i; \tag{6}$$

$$\begin{aligned}\widetilde{E}_{i,\omega_0}\left(\boldsymbol{r},\omega-\omega_0;t\right) &= \mathcal{F}\left[E_{i,\omega_0}\left(\boldsymbol{r},t\right)\exp\left(j\omega_0 t\right)\right] \simeq \sum_{m=1}^{N}\mathcal{F}\left[E_{mi,\omega_0}\left(\boldsymbol{r},t\right)\exp\left(j\omega_0 t\right)\right]\\ &= \sum_{m=1}^{N}\widetilde{\mathtt{A}}_{mi}\left(z,\omega-\omega_0;t\right)F_{mi}\left(x,y,\omega;z,t\right)\exp\left(-j\Phi_{mi}\left(z,\omega;t\right)\right)\\ &= \sum_{m=1}^{N}\widetilde{A}_{mi}\left(z,\omega-\omega_0;t\right)F_{mi}\left(x,y,\omega;z,t\right)\exp\left(-j\phi_{mi}\left(z,\omega_0;t\right) - \frac{1}{2}\alpha\left(\omega\right)z\right),\end{aligned} \tag{7}$$

with $\widetilde{E}_{i,\omega_0}$ the Fourier transform of the complex amplitude E_{i,ω_0}, and $\widetilde{A}_{mi}$ is the Fourier transform of the complex envelope of the optical pulses in the PCM mi comprising the slow longitudinal and temporal variations of the phase, that is, $\widetilde{A}_{mi}(z,\omega-\omega_0;t) = \widetilde{\mathtt{A}}_{mi}(z,\omega-\omega_0;t)\exp[-j\left(\phi_{mi}\left(z,\omega;t\right) - \phi_{mi}\left(z,\omega_0;t\right)\right)]$. Furthermore, in order to preserve the temporal fiber birefringence fluctuations in $\widetilde{A}_{mi}$, the Fourier transform is calculated in the baseband ($\Omega = \omega - \omega_0$) in a time interval of the order of the temporal pulse width T_{P}:

$$\widetilde{A}_{mi}\left(z,\Omega;t\right) := \int_{\langle T_{\mathrm{P}}\rangle} A_{mi}\left(z,t\right)\exp\left(-j\Omega t\right)\mathrm{d}t. \tag{8}$$

Equations (1)-(8) allow us to describe the longitudinal and temporal MCF perturbations in the electric field strength of the MCF. Along this line, the following considerations on the above equations should be remarked:

- The longitudinal and temporal MCF perturbations define the birefringent segments and the local modes in each PCM mi. These fiber perturbations are modelled in the complex envelope $\widetilde{A}_{mi}$, transversal local eigenfunctions F_{mi} and complex phase function Φ_{mi}. Assuming that the longitudinal and temporal MCF perturbations modify the ideal phase constant β_{mi}, the transversal function F_{mi} and the complex envelope $\widetilde{A}_{mi}$ should also be assumed longitudinal and temporal dependent in order to satisfy the Maxwell equations in the MCF segments. In this way, the Fourier transform of the optical pulses $\widetilde{A}_{mi}\left(z,\Omega;t\right)$ is also found to be time dependent due to the slowly-varying temporal fluctuation of the fiber.

- According to Ref. [1], the semicolon symbol is used to separate explicitly longitudinal and temporal changes induced by the slowly-varying MCF perturbations. The longitudinal and temporal changes induced by these perturbations in the optical medium are assumed to be slowly-varying in comparison with the spatial and temporal duration of $A_{mi}(z,t)$. As an example, the slowly-varying longitudinal and temporal changes of the transversal local eigenfunction $F_{mi}(x,y,\omega;z,t)$ are explicitly separated by the semicolon symbol from the transversal changes given by the well-known Bessel functions [6].

- The phase of the local modes is given by the complex function Φ_{mi} in each MCF segment. Therefore, considering that the local modes satisfy the Maxwell equations, F_{mi} should also be assumed a complex function. Nevertheless, taking into account that the imaginary part of Φ_{mi} (modelling the optical absorption) is much lower than the real part ϕ_{mi} (accounting for the ideal phase constant along with the fiber perturbations), we assume F_{mi} as a real function from now on. However, note that in the multi-mode regime, F_{mi} should be considered as a complex function including the azimuthal order of the corresponding LP mode group.

- In contrast with previous ultra-short pulse propagation models proposed for single-mode fibers (SMFs) and MCFs in [3],[7]-[17], the ideal phase constant $\beta_{mi}(\omega)$ is assumed to be modified by the aforementioned perturbations, which are included in the coupled local-mode theory with rigorous formalism from the Maxwell equations. Furthermore, note that the complex phase function $\Phi_{mi}(z,\omega;t)$ is not approximated to ω_0 in order to describe accurately the frequency dependence of the phase-mismatching between the local modes including the MCF birefringence. In this way, we will able to investigate the impact of the fiber perturbations when propagating ultra-short optical pulses in real MCFs.

1.2 Coupled-wave equations

In this subsection, the goal is to derive the coupled-wave equations of the complex amplitude E_{i,ω_0} in each polarization axis $i = x, y$ of the MCF. To this end, we start writing in the optical medium the first and second macroscopic Maxwell equations (Ampère's and Faraday's laws) in the time domain (we use the notation $\partial_t = \partial/\partial t$):

$$\nabla \times \boldsymbol{\mathcal{E}}(\boldsymbol{r},t) = -\partial_t \boldsymbol{\mathcal{B}}(\boldsymbol{r},t)\,; \quad \nabla \times \boldsymbol{\mathcal{H}}(\boldsymbol{r},t) = \partial_t \boldsymbol{\mathcal{D}}(\boldsymbol{r},t)\,, \tag{9}$$

with $\boldsymbol{\mathcal{E}}$ the real wave function of the electric field strength; $\boldsymbol{\mathcal{B}}$ the real wave function of the magnetic induction; $\boldsymbol{\mathcal{H}}$ the real wave function of the magnetic field strength; and $\boldsymbol{\mathcal{D}}$ the real wave function of the electric displacement. From the classical homogenization process [18], we can express the auxiliary field $\boldsymbol{\mathcal{D}}$ and $\boldsymbol{\mathcal{H}}$ in terms of the polarization and magnetization through the so-called constitutive relations, which account for the response of the medium bound charges and currents to the applied fields [all the fields appearing in Eq. (9) are volume-averaged quantities]. Thus, applying the curl operator in Eq. (9) and using the constitutive relations, the wave equation of the electric field strength is found to be:

$$\triangle \boldsymbol{\mathcal{E}}(\boldsymbol{r},t) - \frac{1}{c_0^2}\partial_t^2 \boldsymbol{\mathcal{E}}(\boldsymbol{r},t) = \mu_0 \partial_t^2 \boldsymbol{\mathcal{P}}^{(1)}(\boldsymbol{r},t) + \mu_0 \partial_t^2 \boldsymbol{\mathcal{P}}^{(3)}(\boldsymbol{r},t)\,, \tag{10}$$

where $\triangle$ is the Laplacian operator; c_0 and μ_0 are the speed of light and the magnetic permeability in vacuum, respectively; and $\boldsymbol{\mathcal{P}}^{(1)}$ and $\boldsymbol{\mathcal{P}}^{(3)}$ are the linear and nonlinear polarization of the MCF, respectively. Note that we have neglected the term $\nabla(\nabla \cdot (\boldsymbol{\mathcal{P}}^{(1)} + \boldsymbol{\mathcal{P}}^{(3)}))$ in Eq. (10) considering slowly-varying refractive index profiles and the low birefringent and nonlinear nature of silica MCFs, in line with the assumptions performed in step- and gradual-index SMFs [3],[4],[7],[8]. Now, applying the Fourier transform to Eq. (10) we obtain:

$$\triangle \boldsymbol{E}(\boldsymbol{r},\omega;t) + \frac{\omega^2}{c_0^2}\boldsymbol{E}(\boldsymbol{r},\omega;t) = -\omega^2 \mu_0 \boldsymbol{P}^{(1)}(\boldsymbol{r},\omega;t) - \omega^2 \mu_0 \boldsymbol{P}^{(3)}(\boldsymbol{r},\omega;t)\,, \tag{11}$$

Here, it should be noted that the temporal birefringence fluctuations of the MCF have been explicitly denoted with the semicolon symbol, as was previously defined. Moreover, the Fourier transform of the linear and

nonlinear polarization can be written in terms of their complex amplitudes in the time and frequency domain as:

$$\begin{aligned}\boldsymbol{P}^{(k)}\left(\boldsymbol{r},\omega;t\right) &= \mathcal{F}\left[\boldsymbol{\mathcal{P}}^{(k)}\left(\boldsymbol{r},t\right)\right] = \mathcal{F}\left[\frac{1}{2}\sum_{i=x,y}\left(P^{(k)}_{i,\omega_0}\left(\boldsymbol{r},t\right)\exp\left(j\omega_0 t\right) + P^{(k)}_{i,-\omega_0}\left(\boldsymbol{r},t\right)\exp\left(-j\omega_0 t\right)\right)\hat{u}_i\right] \\ &= \frac{1}{2}\sum_{i=x,y}\left[\widetilde{P}^{(k)}_{i,\omega_0}\left(\boldsymbol{r},\omega-\omega_0;t\right) + \widetilde{P}^{(k)}_{i,-\omega_0}\left(\boldsymbol{r},\omega+\omega_0;t\right)\right]\hat{u}_i; \quad k=1,3. \end{aligned} \tag{12}$$

where the nonlinear polarization in $3\omega_0$ was omitted taking into account that the phase-matching condition in this nonlinear term is not satisfied in silica fibers [7]. Therefore, using Eqs. (6) and (12) the nonlinear wave equation Eq. (11) becomes:

$$\triangle\widetilde{E}_{i,\omega_0}\left(\boldsymbol{r},\omega-\omega_0;t\right) + \frac{\omega^2}{c_0^2}\widetilde{E}_{i,\omega_0}\left(\boldsymbol{r},\omega-\omega_0;t\right) = -\omega^2\mu_0\widetilde{P}^{(1)}_{i,\omega_0}\left(\boldsymbol{r},\omega-\omega_0;t\right) - \omega^2\mu_0\widetilde{P}^{(3)}_{i,\omega_0}\left(\boldsymbol{r},\omega-\omega_0;t\right). \tag{13}$$

Now, let us discuss the expression of the complex amplitude of the linear polarization in the frequency domain $\widetilde{P}^{(1)}_{i,\omega_0}$ as a function of the MCF random perturbations. Considering the dispersive effects and the spatial and temporal random perturbations of the fiber, the linear polarization in the time domain $\boldsymbol{\mathcal{P}}^{(1)}$ can be expressed in the general form:

$$\boldsymbol{\mathcal{P}}^{(1)}\left(\boldsymbol{r},t\right) = \varepsilon_0\int_{-\infty}^{+\infty}\boldsymbol{\chi}^{(1)}\left(\boldsymbol{r},\tau;t\right)\boldsymbol{\mathcal{E}}\left(\boldsymbol{r},\tau\right)\mathrm{d}\tau \simeq \varepsilon_0\int_{\langle T_\mathrm{P}\rangle}\boldsymbol{\chi}^{(1)}\left(\boldsymbol{r},t-\tau\right)\boldsymbol{\mathcal{E}}\left(\boldsymbol{r},\tau\right)\mathrm{d}\tau, \tag{14}$$

with ε_0 the electrical permittivity in vacuum and $\boldsymbol{\chi}^{(1)}$ the first-order electrical susceptibility tensor accounting for the linear and circular birefringence of the MCF. Note that $\boldsymbol{\chi}^{(1)}$ is assumed longitudinal and temporal dependent due to the MCF perturbations and the dispersive nature of silica media. Rigorously, the linear polarization describes a linear and time-varying system. However, at this point let us assume the pulse width T_P much shorter than the temporal birefringence fluctuations of the MCF. Hence, the linear and time-varying system described by the linear polarization can be modelled as a linear and time-invariant system in a time interval T_P, as indicated in the approximation performed in Eq. (14). As a result, the constitutive relation of the linear polarization can be expressed in the frequency domain as:

$$\boldsymbol{P}^{(1)}\left(\boldsymbol{r},\omega;t\right) = \varepsilon_0\widetilde{\boldsymbol{\chi}}^{(1)}\left(\boldsymbol{r},\omega;t\right)\boldsymbol{E}\left(\boldsymbol{r},\omega;t\right), \tag{15}$$

where:

$$\widetilde{\boldsymbol{\chi}}^{(1)}\left(\boldsymbol{r},\omega;t\right) := \mathcal{F}\left[\boldsymbol{\chi}^{(1)}\left(\boldsymbol{r},t\right)\right] = \int_{\langle T_\mathrm{P}\rangle}\boldsymbol{\chi}^{(1)}\left(\boldsymbol{r},t\right)\exp\left(-j\omega t\right)\mathrm{d}t. \tag{16}$$

It should be taken into account that the first-order electrical susceptibility tensor in the frequency domain $\widetilde{\boldsymbol{\chi}}^{(1)}$ describes the material dispersion and the optical absorption induced by the resonant frequencies of silica media, located in the ultraviolet (68.4 nm and 116.2 nm) and infrared (9896.2 nm) bands [2]. Moreover, the optical absorption induced by the Rayleigh scattering is modelled in the attenuation coefficient α of Eq. (3). Thus, the complex amplitude of the linear polarization in the frequency domain is found to be (Einstein summation convention):

$$\widetilde{P}^{(1)}_{i,\omega_0}\left(\boldsymbol{r},\omega-\omega_0;t\right) = \varepsilon_0\widetilde{\chi}^{(1)}_{ij}\left(\boldsymbol{r},\omega;t\right)\widetilde{E}_{j,\omega_0}\left(\boldsymbol{r},\omega-\omega_0;t\right). \tag{17}$$

Now, we still need to investigate the nonlinear polarization term $\widetilde{P}^{(3)}_{i,\omega_0}$ of Eq. (13). Nevertheless, the constitutive relation of the nonlinear polarization with the electric field strength involves the convolution operation in the frequency domain, increasing the complexity of the mathematical discussion of the coupled

local-mode equations. Hence, we will maintain the nonlinear polarization $\widetilde{P}^{(3)}_{i,\omega_0}$ in the wave equation Eq. (13), which is reduced to:

$$\triangle\widetilde{E}_{i,\omega_0}\left(\boldsymbol{r},\omega-\omega_0;t\right)+\frac{\omega^2}{c_0^2}\left[\widetilde{E}_{i,\omega_0}\left(\boldsymbol{r},\omega-\omega_0;t\right)+\widetilde{\chi}^{(1)}_{ij}\left(\boldsymbol{r},\omega;t\right)\widetilde{E}_{j,\omega_0}\left(\boldsymbol{r},\omega-\omega_0;t\right)\right]=-\omega^2\mu_0\widetilde{P}^{(3)}_{i,\omega_0}\left(\boldsymbol{r},\omega-\omega_0;t\right). \tag{18}$$

Furthermore, considering that $\widetilde{\chi}^{(1)}_{yx}=\left(\widetilde{\chi}^{(1)}_{xy}\right)^*$ in dispersive dielectric media in order to satisfy the Poynting theorem [19] and defining:

$$\widetilde{\varepsilon}_{r,i}\left(\boldsymbol{r},\omega;t\right):=1+\widetilde{\chi}^{(1)}_{ii}\left(\boldsymbol{r},\omega;t\right); \tag{19}$$

$$\widetilde{\sigma}\left(\boldsymbol{r},\omega;t\right):=\widetilde{\chi}^{(1)}_{xy}\left(\boldsymbol{r},\omega;t\right), \tag{20}$$

the coupled-wave equations are finally derived (the independent variables are omitted for the sake of simplicity from now on):

$$\triangle\widetilde{E}_{x,\omega_0}+\frac{\omega^2}{c_0^2}\left(\widetilde{\varepsilon}_{r,x}\widetilde{E}_{x,\omega_0}+\widetilde{\sigma}\widetilde{E}_{y,\omega_0}\right)=-\omega^2\mu_0\widetilde{P}^{(3)}_{x,\omega_0}; \tag{21}$$

$$\triangle\widetilde{E}_{y,\omega_0}+\frac{\omega^2}{c_0^2}\left(\widetilde{\varepsilon}_{r,y}\widetilde{E}_{y,\omega_0}+\widetilde{\sigma}^*\widetilde{E}_{x,\omega_0}\right)=-\omega^2\mu_0\widetilde{P}^{(3)}_{y,\omega_0}. \tag{22}$$

Note that, in contrast to Ref. [1], the coupled-wave equations given by Eqs. (21) and (22) are written in the frequency domain when assuming non-monochromatic electric fields in Eq. (1). As a result, the coupled local-mode equations should also be discussed in the frequency domain, and later, we will apply the inverse Fourier transform to obtain the final expressions in the time domain.

1.3 Coupled local-mode equations

Once we derive the coupled-wave equations, we can obtain the coupled local-mode equations when using Eqs. (6) and (7) in Eqs. (21) and (22). However, we should previously discuss some questions related to the MCF birefringence and local modes.

First, let us assume without loss of generality a MCF comprising only two cores a and b. In order to describe different MCF designs[1], the relative electric permittivity given by Eq. (19) and accounting for the linear birefringence is found in each polarization axis as:

$$\begin{aligned}\widetilde{\varepsilon}_{r,i}\left(\boldsymbol{r},\omega;t\right)&=\widetilde{\varepsilon}_{r,ci}\left(\boldsymbol{r},\omega;t\right)+\Delta\widetilde{\varepsilon}_{r,ai}\left(\boldsymbol{r},\omega;t\right)+\Delta\widetilde{\varepsilon}_{r,bi}\left(\boldsymbol{r},\omega;t\right)\\&=\begin{cases}\boldsymbol{r}\equiv\text{core } a & \widetilde{\varepsilon}_{r,ai}\left(\boldsymbol{r},\omega;t\right)=\widetilde{\varepsilon}_{r,ci}\left(\boldsymbol{r},\omega;t\right)+\Delta\widetilde{\varepsilon}_{r,ai}\left(\boldsymbol{r},\omega;t\right)\\\boldsymbol{r}\equiv\text{cladding} & \widetilde{\varepsilon}_{r,ci}\left(\boldsymbol{r},\omega;t\right)\\\boldsymbol{r}\equiv\text{core } b & \widetilde{\varepsilon}_{r,bi}\left(\boldsymbol{r},\omega;t\right)=\widetilde{\varepsilon}_{r,ci}\left(\boldsymbol{r},\omega;t\right)+\Delta\widetilde{\varepsilon}_{r,bi}\left(\boldsymbol{r},\omega;t\right)\end{cases},\end{aligned} \tag{23}$$

where $\widetilde{\varepsilon}_{r,ai}$, $\widetilde{\varepsilon}_{r,bi}$ and $\widetilde{\varepsilon}_{r,ci}$ are the relative electric permittivity in the cores a, b and in the cladding, respectively; and $\Delta\widetilde{\varepsilon}_{r,ai}$ and $\Delta\widetilde{\varepsilon}_{r,bi}$ are the difference between the relative electric permittivity of the cladding and those of cores a and b, respectively. Moreover, the circular birefringence is given by:

$$\widetilde{\sigma}\left(\boldsymbol{r},\omega;t\right)=\begin{cases}\boldsymbol{r}\equiv\text{core } a & \widetilde{\sigma}_a\left(\boldsymbol{r},\omega;t\right)=\widetilde{\chi}^{(1)}_{a,xy}\left(\boldsymbol{r},\omega;t\right)\\\boldsymbol{r}\equiv\text{cladding} & \widetilde{\sigma}_c\left(\boldsymbol{r},\omega;t\right)=\widetilde{\chi}^{(1)}_{c,xy}\left(\boldsymbol{r},\omega;t\right)\\\boldsymbol{r}\equiv\text{core } b & \widetilde{\sigma}_b\left(\boldsymbol{r},\omega;t\right)=\widetilde{\chi}^{(1)}_{b,xy}\left(\boldsymbol{r},\omega;t\right)\end{cases}. \tag{24}$$

[1]The herein presented theory is a general model which can be applied to MCFs comprising: coupled or uncoupled cores, lowly- or highly-birefringent cores, trench- or hole-assisted cladding and gradual- or step-index profile. Note that in contrast to Ref. [1], Eqs. (23) and (24) are assumed to be longitudinal and transversal dependent.

Note that the real part of Eqs. (23) and (24) gives information about the material dispersion and the imaginary part accounts for the optical absorption induced by the resonant frequencies of silica media. Nevertheless, taking into account that the optical absorption is mainly induced by the Rayleigh scattering in the third transmission window [2],[7], the imaginary part of Eqs. (23) and (24) can be neglected. Thus, the fiber parameters $\widetilde{\varepsilon}_{r,i}$ and $\widetilde{\sigma}$ will be assumed real functions from now on.

Second, it is worth mentioning that the idea underneath local modes permits to describe the MCF perturbations with rigorous formalism when considering that the local mode $F_{mi}(x,y,\omega;z,t)\exp(-j\Phi_{mi}(z,\omega;t))$ should satisfy the Helmholtz equation in each birefringent segment of the core m:

$$\left(\triangle + \frac{\omega^2}{c_0^2}\widetilde{\varepsilon}_{r,mi}\right) F_{mi}\exp\left(-j\Phi_{mi}\right) = 0. \tag{25}$$

Equation (25) allows us to obtain two fundamental relations to describe the chromatic dispersion along with the MCF perturbations. It is straightforward to derive these relations performing the next two steps. First, the complex phase function Φ_{mi} should be written as:

$$\begin{aligned}\Phi_{mi}(z,\omega;t) &= \int_0^z \beta_{mi}^{(\mathrm{eq})}\left(\xi,\omega;t\right)\mathrm{d}\xi - j\frac{1}{2}\alpha\left(\omega\right)z \\ &= \int_0^z \sum_{k=0}^{\infty}\frac{1}{k!}\left(\omega-\omega_0\right)^k \beta_{mi,\omega_0}^{(\mathrm{eq})(k)}\left(\xi;t\right)\mathrm{d}\xi - j\frac{1}{2}\alpha\left(\omega\right)z,\end{aligned} \tag{26}$$

with $\beta_{mi,\omega_0}^{(\mathrm{eq})(k)}\left(z;t\right) := \partial_\omega^k \beta_{mi}^{(\mathrm{eq})}\left(z,\omega=\omega_0;t\right)$. Second, we should approximate[2]:

$$\begin{aligned}\left(\beta_{mi}^{(\mathrm{eq})}\right)^2 - \left(\beta_{mi,\omega_0}^{(\mathrm{eq})}\right)^2 &= \left(\beta_{mi}^{(\mathrm{eq})} + \beta_{mi,\omega_0}^{(\mathrm{eq})}\right)\left(\beta_{mi}^{(\mathrm{eq})} - \beta_{mi,\omega_0}^{(\mathrm{eq})}\right) \\ &\simeq 2\beta_{mi,\omega_0}^{(\mathrm{eq})}\left(\beta_{mi}^{(\mathrm{eq})} - \beta_{mi,\omega_0}^{(\mathrm{eq})}\right) \simeq 2\beta_{mi,\omega_0}^{(\mathrm{eq})}\sum_{k=1}^{\infty}\frac{1}{k!}\left(\omega-\omega_0\right)^k\beta_{mi,\omega_0}^{(\mathrm{eq})(k)}.\end{aligned} \tag{27}$$

Thus, assuming that $\partial_z F_{mi} \simeq \partial_z^2 F_{mi} \simeq 0$ in $\delta z \sim \lambda_0$, where λ_0 is the wavelength of the optical carrier at the vacuum[3], we directly obtain from Eq. (25):

$$\left(\triangle_{\mathrm{T}} + \frac{\omega^2}{c_0^2}\widetilde{\varepsilon}_{r,i}\right)F_{ai} = \left[\frac{\omega^2}{c_0^2}\Delta\widetilde{\varepsilon}_{r,bi} + \left(\beta_{ai,\omega_0}^{(\mathrm{eq})}\right)^2 + 2\beta_{ai,\omega_0}^{(\mathrm{eq})}\sum_{k=1}^{\infty}\frac{1}{k!}\left(\omega-\omega_0\right)^k\beta_{ai,\omega_0}^{(\mathrm{eq})(k)} + j\left(\partial_z-\alpha\right)\beta_{ai}^{(\mathrm{eq})}\right]F_{ai}; \tag{28}$$

$$\left(\triangle_{\mathrm{T}} + \frac{\omega^2}{c_0^2}\widetilde{\varepsilon}_{r,i}\right)F_{bi} = \left[\frac{\omega^2}{c_0^2}\Delta\widetilde{\varepsilon}_{r,ai} + \left(\beta_{bi,\omega_0}^{(\mathrm{eq})}\right)^2 + 2\beta_{bi,\omega_0}^{(\mathrm{eq})}\sum_{k=1}^{\infty}\frac{1}{k!}\left(\omega-\omega_0\right)^k\beta_{bi,\omega_0}^{(\mathrm{eq})(k)} + j\left(\partial_z-\alpha\right)\beta_{bi}^{(\mathrm{eq})}\right]F_{bi}, \tag{29}$$

with $\triangle_{\mathrm{T}} = \partial_x^2 + \partial_y^2$ the transversal Laplacian operator.

Now, at this point we can start with the derivation of the coupled local-mode equations by combining Eqs. (7) and Eqs. (21) and (22). After some algebraic work, retaining the second derivatives of the complex envelopes considering that $\partial_z^2\widetilde{A}_{mi} \neq 0$ in $\delta z \sim \lambda_0$ for ultra-short optical pulses [2] and using Eqs. (5), we obtain for the x-polarization axis ($i = x$):

[2]From now on, the subindex ω_0 is used to indicate explicitly the value of the functions at $\omega = \omega_0$.

[3]In fact, the omission of the first and second derivative of F_{mi} only requires to consider longitudinal distances of the order of $\lambda_{mi} = \lambda_0/n_{mi}$, where n_{mi} is the refractive index of the PCM mi at the centre of the pulse bandwidth. As we have considered longitudinal changes of F_{mi} slowly-varying in comparison with the spatial and temporal duration of the pulse envelope, the condition $\partial_z F_{mi} \simeq \partial_z^2 F_{mi} \simeq 0$ is fulfilled not only in $\delta z \sim \lambda_{mi}$, but also in $\delta z \sim \lambda_0$.

$$
\begin{aligned}
&\left[\frac{\omega^2}{c_0^2}\Delta\widetilde{\varepsilon}_{r,bx} - \left(j2\beta^{(\mathrm{eq})}_{ax,\omega_0} + \alpha - \partial_z\right)\mathfrak{D}^{(\mathrm{eq})}_{ax}\right] F_{ax}\exp\left(-j\phi_{ax,\omega_0}\right)\widetilde{A}_{ax} + F_{ax}\exp\left(-j\phi_{ax,\omega_0}\right)\partial_z^2\widetilde{A}_{ax} \\
&+\left[\frac{\omega^2}{c_0^2}\Delta\widetilde{\varepsilon}_{r,ax} - \left(j2\beta^{(\mathrm{eq})}_{bx,\omega_0} + \alpha - \partial_z\right)\mathfrak{D}^{(\mathrm{eq})}_{bx}\right] F_{bx}\exp\left(-j\phi_{bx,\omega_0}\right)\widetilde{A}_{bx} + F_{bx}\exp\left(-j\phi_{bx,\omega_0}\right)\partial_z^2\widetilde{A}_{bx} \\
&\quad-\left(j2\beta^{(\mathrm{eq})}_{ax,\omega_0} + \alpha\right)F_{ax}\exp\left(-j\phi_{ax,\omega_0}\right)\partial_z\widetilde{A}_{ax} - \left(j2\beta^{(\mathrm{eq})}_{bx,\omega_0} + \alpha\right)F_{bx}\exp\left(-j\phi_{bx,\omega_0}\right)\partial_z\widetilde{A}_{bx} \\
&\quad+\frac{\omega^2}{c_0^2}\widetilde{\sigma}\left[\widetilde{A}_{ay}F_{ay}\exp\left(-j\phi_{ay,\omega_0}\right) + \widetilde{A}_{by}F_{by}\exp\left(-j\phi_{by,\omega_0}\right)\right] = -\omega^2\mu_0\widetilde{P}^{(3)}_{x,\omega_0}\exp\left(\frac{1}{2}\alpha z\right),
\end{aligned}
\tag{30}
$$

where $\mathfrak{D}^{(\mathrm{eq})}_{mi}$ is the complex function defined as:

$$
\mathfrak{D}^{(\mathrm{eq})}_{mi} := \sum_{k=1}^{\infty}\frac{j}{k!}\left(\omega-\omega_0\right)^k\beta^{(\mathrm{eq})(k)}_{mi,\omega_0}. \tag{31}
$$

A similar expression as Eq. (30) is obtained for the y-polarization axis by exchanging the subindexes x and y. As can be seen, the left-hand side (LHS) of Eq. (30) describes the linear propagation and the right-hand side (RHS) accounts for the nonlinear effects. Thus, the derivation of the coupled-local mode equations requires to investigate the constitutive relation between the complex amplitudes of the nonlinear polarization and the electric field strength in the frequency domain.

To this end, we should start from the constitutive relation in the time domain. Therefore, at this point let us remember the expressions of the nonlinear polarization vector in the time and frequency domain as a function of the corresponding complex amplitudes:

$$
\boldsymbol{\mathcal{P}}^{(3)}\left(\boldsymbol{r},t\right) = \mathrm{Re}\left\{\sum_{i=x,y}P^{(3)}_{i,\omega_0}\left(\boldsymbol{r},t\right)\exp\left(j\omega_0 t\right)\hat{u}_i\right\}; \tag{32}
$$

$$
\boldsymbol{P}^{(3)}\left(\boldsymbol{r},\omega;t\right) = \mathcal{F}\left[\boldsymbol{\mathcal{P}}^{(3)}\left(\boldsymbol{r},t\right)\right] = \mathrm{Re}\left\{\sum_{i=x,y}\widetilde{P}^{(k)}_{i,\omega_0}\left(\boldsymbol{r},\omega-\omega_0;t\right)\hat{u}_i\right\}, \tag{33}
$$

with:

$$
\widetilde{P}^{(k)}_{i,\omega_0}\left(\boldsymbol{r},\omega-\omega_0;t\right) = \mathcal{F}\left[P^{(3)}_{i,\omega_0}\left(\boldsymbol{r},t\right)\exp\left(j\omega_0 t\right)\right], \tag{34}
$$

and omitting the nonlinear polarization in $3\omega_0$, as was pointed out previously in Eq. (12). Hence, the nonlinear terms of Eq. (30) can be found analyzing the constitutive relation P-E in the time domain and performing the Fourier transform of the complex amplitudes, as indicated in Eq. (34).

In ultra-short optical pulses, the constitutive relation between the nonlinear polarization and the electric field strength should include the delay response of the electronic and nuclei structure of silica atoms when an electric field stimulates the optical medium. The most general expression is given by [3],[4],[7] (Einstein summation convention):

$$
\mathcal{P}^{(3)}_i\left(\boldsymbol{r},t\right) = \varepsilon_0\iiint\chi^{(3)}_{ijkl}\left(t,\tau_1,\tau_2,\tau_3\right)\mathcal{E}_j\left(\boldsymbol{r},\tau_1\right)\mathcal{E}_k\left(\boldsymbol{r},\tau_2\right)\mathcal{E}_l\left(\boldsymbol{r},\tau_3\right)\mathrm{d}\tau_1\mathrm{d}\tau_2\mathrm{d}\tau_3. \tag{35}
$$

For optical frequencies well below the electronic transitions, the electronic contribution to the nonlinear polarization can be considered instantaneous. However, since nucleons (protons and neutrons) are considerably heavier than electrons, the nuclei motions have resonant frequencies much lower than the electronic transitions and, consequently, they should be retained in the constitutive relation. Specifically, Raman scattering is a well-known effect arising from the nuclear contribution to the nonlinear polarization. Therefore, considering

ultra-short optical pulses wider than 1 fs, the electronic response can be assumed instantaneous and Eq. (35) can be approximated to [3]:

$$\begin{aligned}\mathcal{P}_i^{(3)}(\boldsymbol{r},t) &\simeq \mathcal{P}_i^{(3\mathrm{I})}(\boldsymbol{r},t)+\mathcal{P}_i^{(3\mathrm{R})}(\boldsymbol{r},t)\\ &=\varepsilon_0\chi_{ijkl}^{(3\mathrm{I})}(t)\,\mathcal{E}_j(\boldsymbol{r},t)\,\mathcal{E}_k(\boldsymbol{r},t)\,\mathcal{E}_l(\boldsymbol{r},t)+\varepsilon_0\mathcal{E}_j(\boldsymbol{r},t)\int_{-\infty}^{+\infty}\chi_{ijkl}^{(3\mathrm{R})}(t-\tau)\,\mathcal{E}_k(\boldsymbol{r},\tau)\,\mathcal{E}_l(\boldsymbol{r},\tau)\,\mathrm{d}\tau,\end{aligned} \tag{36}$$

where the first term of the RHS describes the instantaneous response (3I) accounting for the electronic resonances, and the second term describes the nuclei motions inducing the intrapulse stimulated Raman scattering effect (3R). The third-order electrical susceptibility tensors of Eq. (36), which are assumed spatial invariant due to the low nonlinear nature of silica MCFs, can be expressed as [20]:

$$\chi_{ijkl}^{(3\mathrm{I})}(t)=\chi_{\mathrm{NL}}\left(\frac{1-f_{\mathrm{R}}}{3}\right)(\delta_{ij}\delta_{kl}+\delta_{ik}\delta_{jl}+\delta_{il}\delta_{jk})\,\delta(t)\,; \tag{37}$$

$$\chi_{ijkl}^{(3\mathrm{R})}(t)=\chi_{\mathrm{NL}}f_{\mathrm{R}}\left[h(t)\,\delta_{ij}\delta_{kl}+\frac{1}{2}u(t)\,(\delta_{ik}\delta_{jl}+\delta_{il}\delta_{jk})\right], \tag{38}$$

where δ_{ij} is the Kronecker delta function; $\chi_{\mathrm{NL}}=1.33\cdot10^{-19}\mathrm{m}^2/\mathrm{W}$ at the wavelength of 1550 nm; $f_{\mathrm{R}}=0.245$ represents the fractional contribution of the delayed Raman response to the nonlinear polarization; and h and u functions describe the isotropic and anisotropic Raman response, respectively [20]:

$$h(t)=f_1\tau_1\left(\tau_1^{-2}+\tau_2^{-2}\right)\exp(-t/\tau_2)\sin(t/\tau_1)\,; \tag{39}$$

$$u(t)=f_2\left(\frac{2\tau_3-t}{\tau_3^2}\right)\exp(-t/\tau_3)+\frac{f_3}{f_1}h(t)\,, \tag{40}$$

with f_1, f_2, f_3, τ_1, τ_2 and τ_3 constants of the nonlinear medium satisfying the following relations:

$$\chi_{\mathrm{NL}}(1-f_{\mathrm{R}})=\tfrac{8}{3}n_{\mathrm{NL}}n_{\mathrm{MCF}};\quad \textstyle\sum_{i=1}^{3}f_i=1;\quad \int_{-\infty}^{+\infty}\left(\chi_{ijkl}^{(3\mathrm{I})}(t)+\chi_{ijkl}^{(3\mathrm{R})}(t)\right)\mathrm{d}t\ =\chi_{\mathrm{NL}}, \tag{41}$$

with $n_{\mathrm{MCF}}\simeq1.45$ the average value of the material refractive index of the MCF; and $n_{\mathrm{NL}}=2.6\cdot10^{-20}$ m^2/W the nonlinear refractive index modelling the changes induced in n_{MCF} by the nonlinear polarization. From Eq. (41) and Ref. [20] we found: $f_1=0.75$, $f_2=0.21$, $f_3=0.04$, $\tau_1=12.2$ fs, $\tau_2=32$ fs,$\tau_3=96$ fs. Figure S1 depicts the isotropous and anisotropous Raman response functions considering $i=j=k=l=x$ in the third-order susceptibility tensor. As can be seen, the delay of the nonlinear polarization induced by the nuclei motions (Raman effect) should be included in the constitutive relation P-E for optical pulses shorter than 200 fs, in line with Eq. (36). In addition, we can note that the isotropous Raman response predominates over the anisotropous response due to the molecular symmetry of the SiO_2.

From Eqs. (32), (36) and (37), it is straightforward to obtain the complex amplitude of the instantaneous nonlinear polarization in the time domain for each polarization axis:

$$P_{x,\omega_0}^{(3\mathrm{I})}=\varepsilon_0\gamma_{\mathrm{I}}\left[\left(|E_{x,\omega_0}|^2+\frac{2}{3}|E_{y,\omega_0}|^2\right)E_{x,\omega_0}+\frac{1}{3}E_{y,\omega_0}^2E_{x,-\omega_0}\right]; \tag{42}$$

$$P_{y,\omega_0}^{(3\mathrm{I})}=\varepsilon_0\gamma_{\mathrm{I}}\left[\left(|E_{y,\omega_0}|^2+\frac{2}{3}|E_{x,\omega_0}|^2\right)E_{y,\omega_0}+\frac{1}{3}E_{x,\omega_0}^2E_{y,-\omega_0}\right], \tag{43}$$

where $\gamma_{\mathrm{I}}:=(3/4)\,\chi_{\mathrm{NL}}(1-f_{\mathrm{R}})=7.5\cdot10^{-20}$ m^2/W at the wavelength of 1550 nm.

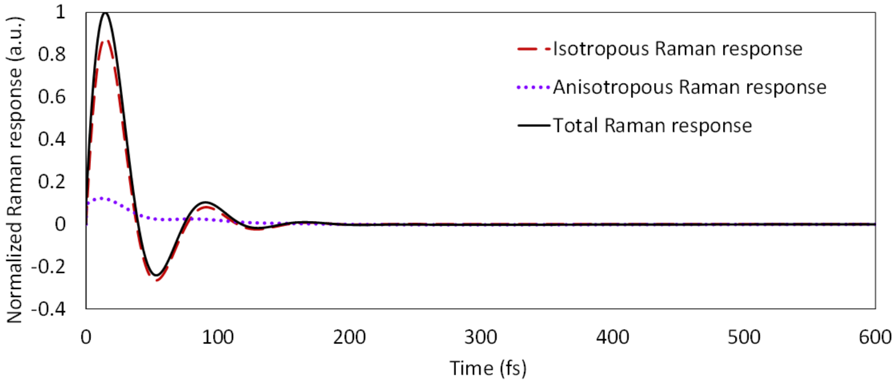

Supplementary Figure S1. Normalized impulse response of the stimulated Raman scattering effect. Dashed line: isotropous response. Dotted line: anisotropous response. Solid line: total Raman response.

Furthermore, the complex amplitude of the nonlinear polarization modelling the nuclei motions (Raman response) can also be found using Eqs. (32), (36) and (38). As an example, the complex amplitude for the x-polarization is derived from these equations as:

$$\begin{aligned} P_{x,\omega_0}^{(3\mathrm{R})}(\boldsymbol{r},t) \;=\;& \frac{1}{4}\varepsilon_0\chi_{\mathrm{NL}}f_{\mathrm{R}}\left\{2E_{x,\omega_0}(\boldsymbol{r},t)\int_{-\infty}^{+\infty}(h+u)(t-\tau)\left|E_{x,\omega_0}(\boldsymbol{r},\tau)\right|^2\mathrm{d}\tau\right.\\ &+2E_{x,\omega_0}(\boldsymbol{r},t)\int_{-\infty}^{+\infty}h(t-\tau)\left|E_{y,\omega_0}(\boldsymbol{r},\tau)\right|^2\mathrm{d}\tau\\ &+2E_{y,\omega_0}(\boldsymbol{r},t)\int_{-\infty}^{+\infty}u(t-\tau)\,\mathrm{Re}\left[E_{x,\omega_0}(\boldsymbol{r},\tau)\,E_{y,-\omega_0}(\boldsymbol{r},\tau)\right]\mathrm{d}\tau\\ &+E_{x,-\omega_0}(\boldsymbol{r},t)\exp(-j2\omega_0 t)\int_{-\infty}^{+\infty}(h+u)(t-\tau)\,E_{x,\omega_0}^2(\boldsymbol{r},\tau)\exp(j2\omega_0\tau)\,\mathrm{d}\tau\\ &+E_{x,-\omega_0}(\boldsymbol{r},t)\exp(-j2\omega_0 t)\int_{-\infty}^{+\infty}h(t-\tau)\,E_{y,\omega_0}^2(\boldsymbol{r},\tau)\exp(j2\omega_0\tau)\,\mathrm{d}\tau\\ &\left.+E_{y,-\omega_0}(\boldsymbol{r},t)\exp(-j2\omega_0 t)\int_{-\infty}^{+\infty}u(t-\tau)\,E_{x,\omega_0}(\boldsymbol{r},\tau)\,E_{y,\omega_0}(\boldsymbol{r},\tau)\exp(j2\omega_0\tau)\,\mathrm{d}\tau\right\}, \end{aligned} \tag{44}$$

including in this case the independent variables to clarify the mathematical discussion. In order to simplify Eq. (44), note that the last three terms of the RHS can be expressed in the general form:

$$E_{j,-\omega_0}(\boldsymbol{r},t)\exp(-j2\omega_0 t)\int_{-\infty}^{+\infty}p(t-\tau)\,E_{k,\omega_0}(\boldsymbol{r},\tau)\,E_{l,\omega_0}(\boldsymbol{r},\tau)\exp(j2\omega_0\tau)\,\mathrm{d}\tau, \tag{45}$$

with $j,k,l=x,y$ and the p function accounting for the h, u and $h+u$ functions. Now, performing a simple algebraic transformation of the convolution integral, Eq. (45) becomes:

$$\begin{aligned} &E_{j,-\omega_0}(\boldsymbol{r},t)\exp(-j2\omega_0 t)\int_{-\infty}^{+\infty}p(t-\tau)\,E_{k,\omega_0}(\boldsymbol{r},\tau)\,E_{l,\omega_0}(\boldsymbol{r},\tau)\exp(j2\omega_0\tau)\,\mathrm{d}\tau\\ &=E_{j,-\omega_0}(\boldsymbol{r},t)\exp(-j2\omega_0 t)\int_{-\infty}^{+\infty}p(\tau)\,E_{k,\omega_0}(\boldsymbol{r},t-\tau)\,E_{l,\omega_0}(\boldsymbol{r},t-\tau)\exp(j2\omega_0(t-\tau))\,\mathrm{d}\tau\\ &=E_{j,-\omega_0}(\boldsymbol{r},t)\int_{-\infty}^{+\infty}p(\tau)\exp(-j2\omega_0\tau)\,E_{k,\omega_0}(\boldsymbol{r},t-\tau)\,E_{l,\omega_0}(\boldsymbol{r},t-\tau)\,\mathrm{d}\tau\\ &=E_{j,-\omega_0}(\boldsymbol{r},t)\left[\left(p(t)\exp(-j2\omega_0 t)\right)*\left(E_{k,\omega_0}(\boldsymbol{r},t)\,E_{l,\omega_0}(\boldsymbol{r},t)\right)\right]. \end{aligned} \tag{46}$$

As can be noted, Eq. (46) involves a convolution of the modulated Raman response with the complex amplitudes of the electric field strength. Considering that the bandwidth of the Raman response (given by $p = h$, u or $h + u$) is around 15 THz [20], centred at $-2\omega_0 \approx -380$ THz, and the bandwidth of the complex amplitudes is lower than 100 THz for ultra-short pulses wider than 10 fs (centred at $\omega = 0$), the Fourier transform of the convolution is found to be null:

$$\mathcal{F}\left[\left(p\left(t\right)\exp\left(-j2\omega_0 t\right)\right) * \left(E_{k,\omega_0}\left(\boldsymbol{r},t\right)E_{l,\omega_0}\left(\boldsymbol{r},t\right)\right)\right] = \frac{1}{2\pi}\widetilde{P}\left(\omega+2\omega_0\right)\left[\widetilde{E}_{k,\omega_0}\left(\boldsymbol{r},\omega;t\right) * \widetilde{E}_{l,\omega_0}\left(\boldsymbol{r},\omega;t\right)\right] = 0, \quad (47)$$

and therefore, the last three terms of the RHS of Eq. (44) can be neglected. Finally, defining the f function as $f := h + u$ and the nonlinear constant $\gamma_{\mathrm{R}} := 0.5\chi_{\mathrm{NL}} f_{\mathrm{R}} = 1.63 \cdot 10^{-20}$ m^2/W at the wavelength of 1550 nm, the complex amplitude of the nonlinear polarization modelling both electronic and nuclei responses is found at the x-polarization axis as:

$$\begin{aligned} P^{(3)}_{x,\omega_0}\left(\boldsymbol{r},t\right) = \varepsilon_0\gamma_{\mathrm{I}}&\left\{\left[\left|E_{x,\omega_0}\left(\boldsymbol{r},t\right)\right|^2 + \frac{2}{3}\left|E_{y,\omega_0}\left(\boldsymbol{r},t\right)\right|^2\right]E_{x,\omega_0}\left(\boldsymbol{r},t\right) + \frac{1}{3}E^2_{y,\omega_0}\left(\boldsymbol{r},t\right)E_{x,-\omega_0}\left(\boldsymbol{r},t\right)\right\} \\ &+ \varepsilon_0\gamma_{\mathrm{R}}\left\{\begin{array}{l} E_{x,\omega_0}\left(\boldsymbol{r},t\right)\left[f\left(t\right) * \left|E_{x,\omega_0}\left(\boldsymbol{r},t\right)\right|^2 + h\left(t\right) * \left|E_{y,\omega_0}\left(\boldsymbol{r},t\right)\right|^2\right] \\ +E_{y,\omega_0}\left(\boldsymbol{r},t\right)\left[u\left(t\right) * \mathrm{Re}\left(E_{x,\omega_0}\left(\boldsymbol{r},\tau\right)E_{y,-\omega_0}\left(\boldsymbol{r},\tau\right)\right)\right] \end{array}\right\}; \end{aligned} \quad (48)$$

and similar for the y-polarization.

Once we know the complex amplitude of the nonlinear polarization in the time domain, Eq. (30) can be completed using Eqs. (34) and (48). To this end, note that the nonlinear constants γ_{I} and γ_{R} are also frequency dependent when operating with ultra-short optical pulses in the femtosecond regime. However, we assume that the frequency variation of these parameters is much lower than their average value in the pulse bandwidth, as same as the frequency changes of F_{mi} and ϕ_{mi}. Hence, Eq. (30) becomes (for the sake of simplicity, the independent variables are only included in the convolution operations of the nonlinear terms):

$$\begin{aligned} \sum_{m=a,b}&\left[\frac{\omega^2}{c_0^2}\left(\widetilde{\varepsilon}_{r,x} - \widetilde{\varepsilon}_{r,mx}\right) - \left(j2\beta^{(\mathrm{eq})}_{mx,\omega_0} + \alpha - \partial_z\right)\mathfrak{D}^{(\mathrm{eq})}_{mx}\right]F_{mx}\exp\left(-j\phi_{mx,\omega_0}\right)\widetilde{A}_{mx} \\ &+F_{mx}\exp\left(-j\phi_{mx,\omega_0}\right)\partial_z^2\widetilde{A}_{mx} - \left(j2\beta^{(\mathrm{eq})}_{mx,\omega_0} + \alpha\right)F_{mx}\exp\left(-j\phi_{mx,\omega_0}\right)\partial_z\widetilde{A}_{mx} \\ &+\frac{\omega^2}{c_0^2}\widetilde{\sigma}F_{my}\exp\left(-j\phi_{my,\omega_0}\right)\widetilde{A}_{my} \\ &+\frac{\omega^2}{c_0^2}\gamma_{\mathrm{I}}\exp\left(-\alpha z\right)\left[\begin{array}{l} F^3_{mx}\exp\left(-j\phi_{mx,\omega_0}\right)\mathcal{F}\left(A_{mx}\left|A_{mx}\right|^2\exp\left(j\omega_0 t\right)\right) \\ +\frac{2}{3}F_{mx}F^2_{my}\exp\left(-j\phi_{mx,\omega_0}\right)\mathcal{F}\left(A_{mx}\left|A_{my}\right|^2\exp\left(j\omega_0 t\right)\right) \\ +\frac{1}{3}F_{mx}F^2_{my}\exp\left(-j\left(2\phi_{my,\omega_0} - \phi_{mx,\omega_0}\right)\right)\mathcal{F}\left(A^*_{mx}A^2_{my}\exp\left(j\omega_0 t\right)\right) + \ldots \end{array}\right] \\ &+\frac{\omega^2}{c_0^2}\gamma_{\mathrm{R}}\exp\left(-\alpha z\right)\left[\begin{array}{l} F^3_{mx}\exp\left(-j\phi_{mx,\omega_0}\right)\mathcal{F}\left(A_{mx}\left[f\left(t\right) * \left|A_{mx}\left(z,t\right)\right|^2\right]\exp\left(j\omega_0 t\right)\right) \\ +F_{mx}F^2_{my}\exp\left(-j\phi_{mx,\omega_0}\right)\mathcal{F}\left(A_{mx}\left[h\left(t\right) * \left|A_{my}\left(z,t\right)\right|^2\right]\exp\left(j\omega_0 t\right)\right) \\ +\frac{1}{2}F_{mx}F^2_{my}\exp\left(-j\phi_{mx,\omega_0}\right) \\ \qquad \times\mathcal{F}\left(A_{my}\left[u\left(t\right) * \left(A_{mx}\left(z,t\right)A^*_{my}\left(z,t\right)\right)\right]\exp\left(j\omega_0 t\right)\right) \\ +\frac{1}{2}F_{mx}F^2_{my}\exp\left(-j\left(2\phi_{my,\omega_0} - \phi_{mx,\omega_0}\right)\right) \\ \qquad \times\mathcal{F}\left(A_{my}\left[u\left(t\right) * \left(A^*_{mx}\left(z,t\right)A_{my}\left(z,t\right)\right)\right]\exp\left(j\omega_0 t\right)\right) + \ldots \end{array}\right] = 0, \end{aligned} \quad (49)$$

and a similar expression is found for the y-polarization axis by exchanging the subindexes x and y in Eq. (49). It should be noted that the nonlinear terms involving cross-coupling effect among the PCMs of different cores have been omitted assuming a MCF with a core-to-core distance higher than three times the core radius with $d_{ab} > 3R_0$ [21]. From Eq. (49), the coupled local-mode equation of each PCM mi in the frequency domain can be found multiplying by $F_{mi}\exp(+j\phi_{mi,\omega_0})$ and integrating in an infinite cross-sectional area of the MCF. As an example, the coupled local-mode equation of the PCM ax is found to be:

$$\begin{aligned} j\left(\partial_z + \mathfrak{D}_{ax}^{(\mathrm{eq})}\right)\widetilde{A}_{ax} = {} & \exp\left(-j\Delta\phi_{ay,ax}^{(0)}\right)\widetilde{m}_{ax,ay}\widetilde{A}_{ay} + \exp\left(-j\Delta\phi_{bx,ax}^{(0)}\right)\widetilde{k}_{ax,bx}\widetilde{A}_{bx} \\ & + \exp\left(-\alpha z\right)\left\{\widetilde{q}_{ax}^{(\mathrm{I})}\mathcal{F}\left(A_{ax}\left|A_{ax}\right|^2\exp\left(j\omega_0 t\right)\right) + \frac{2}{3}\widetilde{g}_{ax,ay}^{(\mathrm{I})}\mathcal{F}\left(A_{ax}\left|A_{ay}\right|^2\exp\left(j\omega_0 t\right)\right)\right. \\ & + \frac{1}{3}\exp\left(-j2\Delta\phi_{ay,ax}^{(0)}\right)\widetilde{g}_{ax,ay}^{(\mathrm{I})}\mathcal{F}\left(A_{ax}^{*}A_{ay}^{2}\exp\left(j\omega_0 t\right)\right) \\ & + \widetilde{q}_{ax}^{(\mathrm{R})}\mathcal{F}\left(A_{ax}\left[f\left(t\right)*\left|A_{ax}\left(z,t\right)\right|^2\right]\exp\left(j\omega_0 t\right)\right) \\ & + \widetilde{g}_{ax,ay}^{(\mathrm{R})}\mathcal{F}\left(A_{ax}\left[h\left(t\right)*\left|A_{ay}\left(z,t\right)\right|^2\right]\exp\left(j\omega_0 t\right)\right) \\ & + \frac{1}{2}\widetilde{g}_{ax,ay}^{(\mathrm{R})}\mathcal{F}\left(A_{ay}\left[u\left(t\right)*\left(A_{ax}\left(z,t\right)A_{ay}^{*}\left(z,t\right)\right)\right]\exp\left(j\omega_0 t\right)\right) \\ & \left. + \frac{1}{2}\exp\left(-j2\Delta\phi_{ay,ax}^{(0)}\right)\widetilde{g}_{ax,ay}^{(\mathrm{R})}\mathcal{F}\left(A_{ay}\left[u\left(t\right)*\left(A_{ax}^{*}\left(z,t\right)A_{ay}\left(z,t\right)\right)\right]\exp\left(j\omega_0 t\right)\right)\right\}, \end{aligned} \tag{50}$$

where $\Delta\phi_{mi,nj}^{(0)} := \phi_{mi,\omega_0} - \phi_{nj,\omega_0}$, $\forall\left(mi,nj\right) \in \{ax,ay,bx,by\}^2$; and the linear and nonlinear coupling coefficients are defined in the frequency domain as:

$$\widetilde{m}_{ax,ay}\left(z,\omega;t\right) := \frac{\omega^2}{2c_0^2\beta_{ax,\omega_0}^{(\mathrm{eq})}N_{ax}}\iint\widetilde{\sigma}F_{ay}F_{ax}\mathrm{d}x\mathrm{d}y; \tag{51}$$

$$\widetilde{k}_{ax,bx}\left(z,\omega;t\right) := \frac{\omega^2}{2c_0^2\beta_{ax,\omega_0}^{(\mathrm{eq})}N_{ax}}\iint\Delta\widetilde{\varepsilon}_{r,ax}F_{bx}F_{ax}\mathrm{d}x\mathrm{d}y; \tag{52}$$

$$\widetilde{q}_{ax}^{(\mathrm{S})}\left(z,\omega;t\right) := \frac{\omega^2\gamma_{\mathrm{S}}\left(\omega\right)}{2c_0^2\beta_{ax,\omega_0}^{(\mathrm{eq})}N_{ax}}\iint F_{ax}^4\mathrm{d}x\mathrm{d}y; \tag{53}$$

$$\widetilde{g}_{ax,ay}^{(\mathrm{S})}\left(z,\omega;t\right) := \frac{\omega^2\gamma_{\mathrm{S}}\left(\omega\right)}{2c_0^2\beta_{ax,\omega_0}^{(\mathrm{eq})}N_{ax}}\iint F_{ay}^2F_{ax}^2\mathrm{d}x\mathrm{d}y; \quad \mathrm{S} \in \{\mathrm{I},\mathrm{R}\}. \tag{54}$$

with $N_{ax}\left(z,\omega;t\right) := \iint F_{ax}^2\mathrm{d}x\mathrm{d}y$. Note that the frequency dependence of the nonlinear coupling coefficients also involves the nonlinear dispersion of γ_{I} and γ_{R}, induced by the frequency dependence of n_{NL}. Specifically, the nonlinear refractive index can be approximated by a first-order Taylor series expansion as $n_{\mathrm{NL}}\left(\omega\right) \simeq n_{\mathrm{NL}}^{(0)} + \left(\omega-\omega_0\right)n_{\mathrm{NL}}^{(1)}$, where $n_{\mathrm{NL}}^{(0)} = 2.6\cdot10^{-20}$ m^2/W and $n_{\mathrm{NL}}^{(1)} = 8.3\cdot10^{-24}$ ps·m^2/W in silica fibers [22].

In general, additional coupling coefficients appear in Eq. (50) modelling linear coupling between the PCMs. Nevertheless, these coupling coefficients can be neglected, as investigated in [1],[21] for the monochromatic case. Now, redefining the complex envelopes in the frequency domain as $\widetilde{A}_{mi} := \widetilde{\mathcal{A}}_{mi}\exp\left(\alpha z/2\right)$, assuming the attenuation coefficient α with low frequency dependence satisfying $\alpha\left(\omega_0\right) \gg \mathrm{d}\alpha\left(\omega=\omega_0\right)/\mathrm{d}\omega$, and defining the equivalent intra- and inter-core mode-coupling functions as:

$$\widetilde{M}_{ax,ay}^{(\mathrm{eq})}\left(z,\omega;t\right) := \exp\left(-j\Delta\phi_{ay,ax}\left(z,\omega_0;t\right)\right)\widetilde{m}_{ax,ay}\left(z,\omega;t\right); \tag{55}$$

$$\widetilde{K}_{ax,bx}^{(\mathrm{eq})}\left(z,\omega;t\right) := \exp\left(-j\Delta\phi_{bx,ax}\left(z,\omega_0;t\right)\right)\widetilde{k}_{ax,bx}\left(z,\omega;t\right), \tag{56}$$

comprising the frequency dependence of the coupling coefficients and the phase-mismatching functions written at $\omega = \omega_0$, Eq. (50) is reduced to:

$$
\begin{aligned}
j\left(\partial_z + \mathfrak{D}_{ax}^{(\mathrm{eq})} + \frac{\alpha}{2}\right)\widetilde{\mathcal{A}}_{ax} = {} & \widetilde{M}_{ax,ay}^{(\mathrm{eq})}\widetilde{\mathcal{A}}_{ay} + \widetilde{K}_{ax,bx}^{(\mathrm{eq})}\widetilde{\mathcal{A}}_{bx} + \widetilde{q}_{ax}^{(\mathrm{I})}\mathcal{F}\left(\mathcal{A}_{ax}\left|\mathcal{A}_{ax}\right|^2\exp\left(j\omega_0 t\right)\right) \\
& + \frac{2}{3}\widetilde{g}_{ax,ay}^{(\mathrm{I})}\mathcal{F}\left(\mathcal{A}_{ax}\left|\mathcal{A}_{ay}\right|^2\exp\left(j\omega_0 t\right)\right) \\
& + \frac{1}{3}\exp(-j2\Delta\phi_{ay,ax}^{(0)})\widetilde{g}_{ax,ay}^{(\mathrm{I})}\mathcal{F}\left(\mathcal{A}_{ax}^{*}\mathcal{A}_{ay}^{2}\exp\left(j\omega_0 t\right)\right) \\
& + \widetilde{q}_{ax}^{(\mathrm{R})}\mathcal{F}\left(\mathcal{A}_{ax}\left[f\left(t\right) * \left|\mathcal{A}_{ax}\left(z,t\right)\right|^2\right]\exp\left(j\omega_0 t\right)\right) \\
& + \widetilde{g}_{ax,ay}^{(\mathrm{R})}\mathcal{F}\left(\mathcal{A}_{ax}\left[h\left(t\right) * \left|\mathcal{A}_{ay}\left(z,t\right)\right|^2\right]\exp\left(j\omega_0 t\right)\right) \\
& + \frac{1}{2}\widetilde{g}_{ax,ay}^{(\mathrm{R})}\mathcal{F}\left(\mathcal{A}_{ay}\left[u\left(t\right) * \left(\mathcal{A}_{ax}\left(z,t\right)\mathcal{A}_{ay}^{*}\left(z,t\right)\right)\right]\exp\left(j\omega_0 t\right)\right) \\
& + \frac{1}{2}\exp(-j2\Delta\phi_{ay,ax}^{(0)})\widetilde{g}_{ax,ay}^{(\mathrm{R})}\mathcal{F}\left(\mathcal{A}_{ay}\left[u\left(t\right) * \left(\mathcal{A}_{ax}^{*}\left(z,t\right)\mathcal{A}_{ay}\left(z,t\right)\right)\right]\exp\left(j\omega_0 t\right)\right).
\end{aligned} \tag{57}
$$

In order to derive the final expression of the coupled-local mode equation for the PCM ax in the time domain, we should perform the following Taylor series expansion at $\omega = \omega_0$ of the holomorphic functions:

$$\widetilde{M}_{ax,ay}^{(\mathrm{eq})}\left(z,\omega;t\right) = \sum_{n=0}^{\infty}\frac{1}{n!}\left(\omega-\omega_0\right)^n\partial_\omega^n\widetilde{M}_{ax,ay}^{(\mathrm{eq})}\left(z,\omega_0;t\right) = \sum_{n=0}^{\infty}\frac{1}{n!}\left(\omega-\omega_0\right)^n\widetilde{M}_{ax,ay}^{(\mathrm{eq})(n)}; \tag{58}$$

$$\widetilde{K}_{ax,bx}^{(\mathrm{eq})}\left(z,\omega;t\right) = \sum_{n=0}^{\infty}\frac{1}{n!}\left(\omega-\omega_0\right)^n\partial_\omega^n\widetilde{K}_{ax,bx}^{(\mathrm{eq})}\left(z,\omega_0;t\right) = \sum_{n=0}^{\infty}\frac{1}{n!}\left(\omega-\omega_0\right)^n\widetilde{K}_{ax,bx}^{(\mathrm{eq})(n)}; \tag{59}$$

$$\widetilde{q}_{ax}^{(\mathrm{S})}\left(z,\omega;t\right) = \sum_{n=0}^{\infty}\frac{1}{n!}\left(\omega-\omega_0\right)^n\partial_\omega^n\widetilde{q}_{ax}^{(\mathrm{S})}\left(z,\omega_0;t\right) = \sum_{n=0}^{\infty}\frac{1}{n!}\left(\omega-\omega_0\right)^n\widetilde{q}_{ax}^{(\mathrm{S})(n)}; \tag{60}$$

$$\widetilde{g}_{ax,ay}^{(\mathrm{S})}\left(z,\omega;t\right) = \sum_{n=0}^{\infty}\frac{1}{n!}\left(\omega-\omega_0\right)^n\partial_\omega^n\widetilde{g}_{ax,ay}^{(\mathrm{S})}\left(z,\omega_0;t\right) = \sum_{n=0}^{\infty}\frac{1}{n!}\left(\omega-\omega_0\right)^n\widetilde{g}_{ax,ay}^{(\mathrm{S})(n)}; \quad \mathrm{S} \in \{\mathrm{I},\mathrm{R}\}; \tag{61}$$

$$\alpha\left(\omega\right) \simeq \alpha\left(\omega_0\right) + \left(\omega-\omega_0\right)\left.\frac{\mathrm{d}\alpha\left(\omega\right)}{\mathrm{d}\omega}\right]_{\omega_0} = \alpha^{(0)} + \left(\omega-\omega_0\right)\alpha^{(1)}, \tag{62}$$

which can be expressed in the time domain by the next linear operators:

$$
\begin{aligned}
\widehat{\mathrm{M}}_{ax,ay}^{(\mathrm{eq})} &:= \sum_{n=0}^{\infty}\frac{\left(-j\right)^n}{n!}\widetilde{M}_{ax,ay}^{(\mathrm{eq})(n)}\partial_t^n; \quad \widehat{\mathrm{K}}_{ax,bx}^{(\mathrm{eq})} := \sum_{n=0}^{\infty}\frac{\left(-j\right)^n}{n!}\widetilde{K}_{ax,bx}^{(\mathrm{eq})(n)}\partial_t^n; \\
\hat{\mathrm{q}}_{ax}^{(\mathrm{S})} &:= \sum_{n=0}^{\infty}\frac{\left(-j\right)^n}{n!}\widetilde{q}_{ax}^{(\mathrm{S})(n)}\partial_t^n; \quad \hat{\mathrm{g}}_{ax,ay}^{(\mathrm{S})} := \sum_{n=0}^{\infty}\frac{\left(-j\right)^n}{n!}\widetilde{g}_{ax,ay}^{(\mathrm{S})(n)}\partial_t^n; \quad \widehat{\alpha} := \alpha^{(0)} - j\alpha^{(1)}\partial_t,
\end{aligned} \tag{63}
$$

and the complex function $\mathfrak{D}_{mi}^{(\mathrm{eq})}$ of the PCM mi is expressed in the time domain by the equivalent dispersion operator, which accounts for the frequency dependence of the ideal phase constant $\beta_{mi}\left(\omega\right)$ and the MCF perturbations $\beta_{mi}^{(\mathrm{B+S})}\left(z,\omega;t\right)$:

$$\widehat{\mathrm{D}}_{mi}^{(\mathrm{eq})} := \sum_{n=1}^{\infty}\frac{\left(-j\right)^{n-1}}{n!}\beta_{mi,\omega_0}^{(\mathrm{eq})(n)}\partial_t^n. \tag{64}$$

Furthermore, in order to reduce the computational complexity of the nonlinear terms in the time domain when using the split-step Fourier method (see Section 4), we can approximate the nonlinear coupling coefficients by a first-order Taylor series expansion. In this way we omit the higher-order effects of the nonlinear polarization-mode dispersion (PMD) in Eq. (57). However, the first-order nonlinear PMD seems to be sufficiently accurate in very-high data rate transmission systems and particularly in those that employ solitons [23]. Finally, applying the inverse Fourier transform to Eq. (57) and considering a MCF comprising N cores, the final expression of the coupled local-mode equation for the PCM ax is found to be in the time domain:

$$\begin{aligned} j\left(\partial_z + \widehat{\mathrm{D}}_{ax}^{(\mathrm{eq})} + \frac{1}{2}\widehat{\alpha}\right)\mathcal{A}_{ax}(z,t) = {} & \widehat{\mathrm{M}}_{ax,ay}^{(\mathrm{eq})}\mathcal{A}_{ay}(z,t) + \sum_{m=b}^{N}\widehat{\mathrm{K}}_{ax,mx}^{(\mathrm{eq})}\mathcal{A}_{mx}(z,t) \\ & + \hat{\mathrm{q}}_{ax}^{(\mathrm{I})}\left(\left|\mathcal{A}_{ax}(z,t)\right|^2\mathcal{A}_{ax}(z,t)\right) + \frac{2}{3}\hat{\mathrm{g}}_{ax,ay}^{(\mathrm{I})}\left(\left|\mathcal{A}_{ay}(z,t)\right|^2\mathcal{A}_{ax}(z,t)\right) \\ & + \frac{1}{3}\exp\left(-j2\Delta\phi_{ay,ax}^{(0)}(z;t)\right)\hat{\mathrm{g}}_{ax,ay}^{(\mathrm{I})}\left(\mathcal{A}_{ax}^{*}(z,t)\,\mathcal{A}_{ay}^{2}(z,t)\right) \\ & + \hat{\mathrm{q}}_{ax}^{(\mathrm{R})}\left[\left(f(t) * \left|\mathcal{A}_{ax}(z,t)\right|^2\right)\mathcal{A}_{ax}(z,t)\right] \\ & + \hat{\mathrm{g}}_{ax,ay}^{(\mathrm{R})}\left[\left(h(t) * \left|\mathcal{A}_{ay}(z,t)\right|^2\right)\mathcal{A}_{ax}(z,t)\right] \\ & + \frac{1}{2}\hat{\mathrm{g}}_{ax,ay}^{(\mathrm{R})}\left\{\left[u(t) * \left(\mathcal{A}_{ax}(z,t)\,\mathcal{A}_{ay}^{*}(z,t)\right)\right]\mathcal{A}_{ay}(z,t)\right\} \\ & + \frac{1}{2}\exp\left(-j2\Delta\phi_{ay,ax}^{(0)}(z;t)\right)\hat{\mathrm{g}}_{ax,ay}^{(\mathrm{R})}\left\{\left[u(t) * \left(\mathcal{A}_{ax}^{*}(z,t)\,\mathcal{A}_{ay}(z,t)\right)\right]\mathcal{A}_{ay}(z,t)\right\}. \end{aligned} \tag{65}$$

The theoretical model is completed by $2N-1$ additional coupled equations for the PCMs $mi \neq ax$, which can be obtained just by exchanging the corresponding subindexes in the last equation. Remarkably, the coupled local-mode equations allow us to describe accurately the linear and nonlinear propagation of each PCM and the linear and nonlinear mode-coupling dispersion (MCD), i.e. the intermodal dispersion between different $\mathrm{LP}_{01,mi}$ modes, including the longitudinal and temporal MCF birefringent effects. A detailed discussion of Eq. (65) can be found in the main text. Now, in the next section, we will take a closer look at the behaviour of the inter-core mode-coupling dispersion (IMCD) in ideal and real MCFs.

2 Theoretical analysis of the IMCD in ideal and real MCFs

In order to gain physical insight, let us describe in more detail the behaviour of the IMCD when operating in the linear regime. Specifically, we perform a theoretical discussion of the IMCD induced by the coupling-coefficient dispersion (CCD) and the phase-mismatching dispersion (PhMD) from Eq. (50) by omitting the optical power attenuation, the PMD (intra-core MCD) and the nonlinear effects. First, we investigate the IMCD in ideal homogeneous MCFs without fiber perturbations, and later, we analyse the effects of the IMCD in real MCFs comprising both homogeneous and heterogeneous cores with random birefringent effects.

2.1 Ideal homogeneous MCFs

As a first example, consider an ideal homogeneous 2-core MCF (cores a and b) without longitudinal and temporal birefringent effects. In this scenario, if we omit the power attenuation, the PMD, the nonlinear effects and perform the transformation[4] $\widetilde{A}_{mi}(z,\omega-\omega_0;t) = \widetilde{\mathtt{A}}_{mi}(z,\omega-\omega_0;t)\exp[-j\left(\phi_{mi}(z,\omega;t) - \phi_{mi}(z,\omega_0;t)\right)]$ in the coupled local-mode equations of the PCMs ax and bx in the frequency domain [i.e. Eq. (50) for the PCM ax and the equivalent equation for the PCM bx], the following expressions are found:

[4] The aforementioned transformation allows us to discuss the IMCD from the complex Eq. (50) with a simple and intuitive physical interpretation in ideal and real MCFs.

$$j\partial_z \widetilde{\mathtt{A}}_{ax}(z,\omega-\omega_0) = \exp\left(-j\Delta\beta_{bx,ax}(\omega)\, z\right) \widetilde{k}_{ax,bx}(\omega)\, \widetilde{\mathtt{A}}_{bx}(z,\omega-\omega_0)\,; \tag{66}$$

$$j\partial_z \widetilde{\mathtt{A}}_{bx}(z,\omega-\omega_0) = \exp\left(+j\Delta\beta_{bx,ax}(\omega)\, z\right) \widetilde{k}_{bx,ax}(\omega)\, \widetilde{\mathtt{A}}_{ax}(z,\omega-\omega_0)\,. \tag{67}$$

It should be noted from the above equations that, in ideal homogeneous MCFs, the phase-mismatching $\Delta\beta_{bx,ax}(\omega)$ between both PCMs becomes null considering that $\beta_{ax} = \beta_{bx}$, and the coupling coefficients are found to be identical with $\widetilde{k}_{ax,bx} = \widetilde{k}_{bx,ax}$. In this way, Eqs. (66) and (67) indicate that an ideal homogeneous MCF is analogous to a symmetric optical coupler. Hence, assuming that only the PCM ax is excited at the MCF input, the solution of the previous equations is given by the expressions ($\Omega = \omega - \omega_0$):

$$\widetilde{\mathtt{A}}_{ax}(z,\Omega) = \cos\left(\widetilde{k}(\Omega)\, z\right) \widetilde{\mathtt{A}}_{ax}(0,\Omega)\,; \qquad \widetilde{\mathtt{A}}_{bx}(z,\Omega) = -j\sin\left(\widetilde{k}(\Omega)\, z\right) \widetilde{\mathtt{A}}_{ax}(0,\Omega)\,, \tag{68}$$

where the coupling coefficient $\widetilde{k}$ is defined as $\widetilde{k}(\Omega) := \widetilde{k}_{ax,bx}(\Omega + \omega_0)$. The previous equations define two different linear and time-invariant (LTI) systems with the transfer functions H_{ax} and H_{bx} modelling the propagation of the complex envelopes $\widetilde{\mathtt{A}}_{mi}$ in the PCMs ax and bx:

$$H_{ax}(z,\Omega) := \frac{\widetilde{\mathtt{A}}_{ax}(z,\Omega)}{\widetilde{\mathtt{A}}_{ax}(0,\Omega)} = \cos\left(\widetilde{k}(\Omega)\, z\right); \qquad H_{bx}(z,\Omega) := \frac{\widetilde{\mathtt{A}}_{bx}(z,\Omega)}{\widetilde{\mathtt{A}}_{ax}(0,\Omega)} = -j\sin\left(\widetilde{k}(\Omega)\, z\right). \tag{69}$$

Now, in order to analyse the first-order CCD, let us perform a first-order Taylor series expansion of the coupling coefficient:

$$\widetilde{k}(\Omega) \simeq \widetilde{k}(\Omega = 0) + \Omega \left.\frac{\mathrm{d}\widetilde{k}(\Omega)}{\mathrm{d}\Omega}\right|_{\Omega=0} = \widetilde{k}^{(0)} + \Omega \widetilde{k}^{(1)}. \tag{70}$$

From Eqs. (69) and (70) it is straightforward to calculate the impulse response of each LTI system:

$$h_{ax}(z,t) = \mathcal{F}^{-1}\left[H_{ax}(z,\Omega)\right] = \frac{1}{2}\delta\left(t - \widetilde{k}^{(1)} z\right) \exp\left(-j\widetilde{k}^{(0)} z\right) + \frac{1}{2}\delta\left(t + \widetilde{k}^{(1)} z\right) \exp\left(+j\widetilde{k}^{(0)} z\right); \tag{71}$$

$$h_{bx}(z,t) = \mathcal{F}^{-1}\left[H_{bx}(z,\Omega)\right] = \frac{1}{2}\delta\left(t - \widetilde{k}^{(1)} z\right) \exp\left(-j\widetilde{k}^{(0)} z\right) - \frac{1}{2}\delta\left(t + \widetilde{k}^{(1)} z\right) \exp\left(+j\widetilde{k}^{(0)} z\right). \tag{72}$$

Consequently, we can note that the linear frequency dependence of the coupling coefficient (first-order CCD) induces the temporal splitting of the optical pulse in each PCM, according to the observations reported in [9]-[17]. Therefore, the first-order CCD can be observed in optical pulses with a temporal width T_{P} satisfying the condition $2\widetilde{k}^{(1)} z \geq T_{\mathrm{P}}$. More specifically, the longitudinal evolution of an optical pulse launched into the PCM ax in $z = 0$ can be easily calculated using Eqs. (71) and (72):

$$\begin{aligned}\mathtt{A}_{ax}(z,t) = \mathtt{A}_{ax}(0,t) * h_{ax}(z,t) &= \frac{1}{2}\mathtt{A}_{ax}\left(0, t - \widetilde{k}^{(1)} z\right) \exp\left(-j\widetilde{k}^{(0)} z\right) + \frac{1}{2}\mathtt{A}_{ax}\left(0, t + \widetilde{k}^{(1)} z\right) \exp\left(+j\widetilde{k}^{(0)} z\right) \\ &\equiv \frac{1}{2}\mathtt{A}_{ax}^{-} \exp(-j\theta) + \frac{1}{2}\mathtt{A}_{ax}^{+} \exp(+j\theta)\,; \end{aligned} \tag{73}$$

$$\begin{aligned}\mathtt{A}_{bx}(z,t) = \mathtt{A}_{ax}(0,t) * h_{bx}(z,t) &= \frac{1}{2}\mathtt{A}_{ax}\left(0, t - \widetilde{k}^{(1)} z\right) \exp\left(-j\widetilde{k}^{(0)} z\right) - \frac{1}{2}\mathtt{A}_{ax}\left(0, t + \widetilde{k}^{(1)} z\right) \exp\left(+j\widetilde{k}^{(0)} z\right) \\ &\equiv \frac{1}{2}\mathtt{A}_{ax}^{-} \exp(-j\theta) - \frac{1}{2}\mathtt{A}_{ax}^{+} \exp(+j\theta)\,, \end{aligned} \tag{74}$$

and in terms of optical intensity (with $P_{mi} = |\mathtt{A}_{mi}|^2$):

$$P_{ax}(z,t) = \frac{1}{4}\left|\mathtt{A}_{ax}^{-}\right|^2 + \frac{1}{4}\left|\mathtt{A}_{ax}^{+}\right|^2 + \frac{1}{4}\mathtt{A}_{ax}^{-}\left(\mathtt{A}_{ax}^{+}\right)^* \exp(-j2\theta) + \frac{1}{4}\left(\mathtt{A}_{ax}^{-}\right)^* \mathtt{A}_{ax}^{+} \exp(+j2\theta)\,; \tag{75}$$

$$P_{bx}(z,t) = \frac{1}{4}\left|\mathtt{A}_{ax}^{-}\right|^2 + \frac{1}{4}\left|\mathtt{A}_{ax}^{+}\right|^2 - \frac{1}{4}\mathtt{A}_{ax}^{-}\left(\mathtt{A}_{ax}^{+}\right)^* \exp(-j2\theta) - \frac{1}{4}\left(\mathtt{A}_{ax}^{-}\right)^* \mathtt{A}_{ax}^{+} \exp(+j2\theta)\,. \tag{76}$$

If we assume MCF distances satisfying that $z \geq T_\text{P}/2\widetilde{k}^{(1)}$, thus $\mathtt{A}_{ax}^{-}\mathtt{A}_{ax}^{+} = 0$ and the third and fourth terms of Eqs. (75) and (76) become null obtaining a similar solution in each PCM ax and bx. The original pulse splits in four identical pulses with the 25% of the initial peak power:

$$P_{ax}(z,t) = P_{bx}(z,t) = \frac{1}{4}P_{ax}\left(0, t - \widetilde{k}^{(1)}z\right) + \frac{1}{4}P_{ax}\left(0, t + \widetilde{k}^{(1)}z\right). \tag{77}$$

Higher-order effects of the CCD induce an additional chirp in the complex envelope of the original pulse, as verified in the next section by performing numerical calculations of Eq. (65) [see Fig. S2(c)]. The CCD was previously investigated in symmetric optical couplers and ideal MCFs in [9]-[16]. Therefore, our model is in line with previous works when considering ideal conditions in homogeneous MCFs without including longitudinal and temporal birefringence perturbations or heterogeneous cores. In [17] the heterogeneous case was analysed with a similar approach as in [9]-[16], modelling a two-core fiber as an ideal optical coupler, i.e., omitting the realistic perturbations of the medium. The power of our model and the original contribution of this work reveals itself when these realistic fiber conditions are considered.

On the other hand, an additional consideration should be remarked from the above equations. Note that the same analysis can be performed to investigate the mode-coupling between two orthogonal PCMs of a given core (e.g. core a) assuming constant twisting conditions and omitting the intrinsic linear birefringence of the fiber. Specifically, the mode-coupling between the PCMs ax-ay can also be modelled by Eqs. (66) and (67) replacing the subindex bx by ay and the coupling coefficients $\widetilde{k}_{ax,bx}$ and $\widetilde{k}_{bx,ax}$ by $\widetilde{m}_{ax,ay}$ and $\widetilde{m}_{ay,ax}$, respectively. Therefore, as can be deduced from the above considerations, the pulse splitting should also be observed between orthogonal PCMs of a given core. However, it is straightforward to verify using Eq. (51) and the equivalent refractive index model detailed in Section 4 that $\partial_\omega \widetilde{m}_{ax,ay} \ll \widetilde{k}^{(1)}$. Consequently, it is difficult to observe the CCD and the pulse splitting effect between orthogonal PCMs of a given core. The intra-core MCD is mainly induced in the linear fiber regime by the frequency dependence of the phase-mismatching $\Delta\phi_{ay,ax}(z,\omega;t)$, termed in the literature as the linear PMD.

2.2 Real homogeneous and heterogeneous MCFs

Now, consider a short MCF segment in a time interval where the longitudinal and temporal fiber perturbations can be assumed to be constant[5]. In addition, in order to gain physical interpretation into the IMCD effects when the fiber birefringence is involved, let us assume once again only two PCMs ax and bx and perform the transformation $\widetilde{A}_{mi}(z,\omega-\omega_0;t) = \widetilde{\mathtt{A}}_{mi}(z,\omega-\omega_0;t)\exp[-j\left(\phi_{mi}(z,\omega;t) - \phi_{mi}(z,\omega_0;t)\right)]$ in the corresponding coupled local-mode equations of the PCMs ax and bx in the frequency domain, that is Eq. (50) for the PCM ax and the equivalent equation for the PCM bx. As a result, the following expressions are found:

$$j\partial_z \widetilde{\mathtt{A}}_{ax}(z,\omega-\omega_0) = \exp\left(-j\Delta\phi_{bx,ax}(z,\omega)\right)\widetilde{k}_{ax,bx}(\omega)\,\widetilde{\mathtt{A}}_{bx}(z,\omega-\omega_0); \tag{78}$$

$$j\partial_z \widetilde{\mathtt{A}}_{bx}(z,\omega-\omega_0) = \exp\left(+j\Delta\phi_{bx,ax}(z,\omega)\right)\widetilde{k}_{bx,ax}(\omega)\,\widetilde{\mathtt{A}}_{ax}(z,\omega-\omega_0), \tag{79}$$

where the frequency dependence of $\mathfrak{D}_{ax}^{(\text{eq})}$ and $\mathfrak{D}_{bx}^{(\text{eq})}$ is now described by the exponential terms, and the time dependence of the complex envelopes and the coupling coefficients disappears in the frequency domain when assuming time-invariant birefringent effects. Furthermore, the phase-mismatching function can be approximated in the short MCF segment to $\Delta\phi_{bx,ax}(z,\omega) \simeq \Delta\beta_{bx,ax}^{(\text{eq})}(\omega)\,z$ if we assume that $f_\text{T}\cdot L_\text{seg} \to 0$, where f_T is the twist rate and L_seg is the segment length. Hence, solving Eqs. (78) and (79) assuming that only the PCM ax is excited at the MCF input, we conclude that a real MCF with longitudinal birefringent effects can be modelled as two different LTI systems with the following transfer functions in baseband ($\Omega = \omega - \omega_0$):

[5] These initial assumptions allow us to investigate theoretically the IMCD in real MCFs comprising cores of different types: heterogeneous, homogeneous, coupled, uncoupled, lowly or highly-birefringent, trench- or hole-assisted, and with step- or gradual-index profile.

$$H_{ax}(z,\Omega) := \frac{\widetilde{\mathtt{A}}_{ax}(z,\Omega)}{\widetilde{\mathtt{A}}_{ax}(0,\Omega)} = \exp\left(-j\frac{\Delta\beta^{(\mathrm{eq})}_{bx,ax}(\Omega+\omega_0)}{2}z\right)\left[\cos\left(\widetilde{\eta}(\Omega)z\right) + j\frac{\Delta\beta^{(\mathrm{eq})}_{bx,ax}(\Omega+\omega_0)}{2\widetilde{\eta}(\Omega)}\sin\left(\widetilde{\eta}(\Omega)z\right)\right]; \tag{80}$$

$$H_{bx}(z,\Omega) := \frac{\widetilde{\mathtt{A}}_{bx}(z,\Omega)}{\widetilde{\mathtt{A}}_{ax}(0,\Omega)} = -j\frac{\widetilde{k}_{bx,ax}(\Omega+\omega_0)}{\widetilde{\eta}(\Omega)}\exp\left(+j\frac{\Delta\beta^{(\mathrm{eq})}_{bx,ax}(\Omega+\omega_0)}{2}z\right)\sin\left(\widetilde{\eta}(\Omega)z\right), \tag{81}$$

where $\Delta\beta^{(\mathrm{eq})}_{bx,ax}$ is the mismatching of the equivalent phase constants between the PCMs ax and bx in the MCF segment, which includes the fiber perturbations and the intrinsic index-mismatching when the MCF comprises heterogeneous cores; and $\widetilde{\eta}(\Omega)$ is the complex function defined as:

$$\widetilde{\eta}(\Omega) := \left[\widetilde{k}_{ax,bx}(\Omega+\omega_0)\,\widetilde{k}_{bx,ax}(\Omega+\omega_0) + \left(\Delta\beta^{(\mathrm{eq})}_{bx,ax}(\Omega+\omega_0)\right)^2/4\right]^{1/2}. \tag{82}$$

In the same way as in ideal homogeneous MCFs, performing the following first-order Taylor series approximation $\Delta\beta^{(\mathrm{eq})}_{bx,ax} \simeq \Delta\beta^{(\mathrm{eq})(0)}_{bx,ax} + \Delta\beta^{(\mathrm{eq})(1)}_{bx,ax}\Omega$ and $\widetilde{\eta}(\Omega) \simeq \widetilde{\eta}^{(0)} + \widetilde{\eta}^{(1)}\Omega$ (with $\Delta\beta^{(\mathrm{eq})(n)}_{bx,ax} := \mathrm{d}^n\Delta\beta^{(\mathrm{eq})}_{bx,ax}(\Omega=0)/\mathrm{d}\Omega^n$ and $\widetilde{\eta}^{(n)} := \mathrm{d}^n\widetilde{\eta}(\Omega=0)/\mathrm{d}\Omega^n$), the transfer functions of the LTI systems are found to be proportional to:

$$H_{ax}(z,\Omega) \propto \exp\left(-j\frac{\Delta\beta^{(\mathrm{eq})(1)}_{bx,ax}}{2}z\Omega\right)\left[\cos\left(\widetilde{\eta}^{(0)}z + \widetilde{\eta}^{(1)}z\Omega\right) + j\frac{\Delta\beta^{(\mathrm{eq})}_{bx,ax}(\Omega+\omega_0)}{2\widetilde{\eta}(\Omega)}\sin\left(\widetilde{\eta}^{(0)}z + \widetilde{\eta}^{(1)}z\Omega\right)\right]; \tag{83}$$

$$H_{bx}(z,\Omega) \propto -j\frac{\widetilde{k}_{bx,ax}(\Omega+\omega_0)}{\widetilde{\eta}(\Omega)}\exp\left(+j\frac{\Delta\beta^{(\mathrm{eq})(1)}_{bx,ax}}{2}z\Omega\right)\sin\left(\widetilde{\eta}^{(0)}z + \widetilde{\eta}^{(1)}z\Omega\right). \tag{84}$$

Equations (83) and (84) allow us to discuss the main implications of the IMCD without performing numerical simulations of the coupled local-mode equations. These conclusions are detailed in the main text and verified via numerical calculations of the coupled local-mode equations.

3 Numerical examples

In this section we present some numerical examples of the IMCD effects. In order to achieve a better comprehension of the IMCD, we omit the optical power attenuation and the nonlinear effects. However, the chromatic dispersion [also known as the group-velocity dispersion (GVD)] and the PMD (induced by the circular birefringence along with the intrinsic linear birefringence of the fiber) will only be considered when is specified. In all the analysed cases, the MCF comprises a fiber length of $L = 40$ m and two cores a and b distributed in a square lattice with a core-to-core distance $d_{ab} = 26$ μm and a core radius $R_0 = 4$ μm, as depicted in Fig. 1 of the manuscript. Moreover, the wavelength of the optical carrier (λ_0) was selected in the third transmission window at $\lambda_0 = 1550$ nm, the peak power of the optical pulses was taken to be 0 dBm, and the time domain was normalized using the group delay of the PCM ax as a reference with $t_\mathrm{N} = (t - \beta^{(1)}_{ax}z)/T_\mathrm{P}$, where T_P is the full-width at $1/2e$ ($\sim 18\%$) of the peak power. The specific parameters of each simulation are detailed in Tables S1 and S2 at the end of this document (see Section 4).

3.1 Ideal homogeneous multi-core fiber: first- and higher-order CCD

As a first investigation of the IMCD, let us now analyse numerically the effects of the CCD when considering an ideal homogeneous MCF without fiber perturbations. The refractive index in the cores and the cladding were fixed to 1.452 and 1.444, respectively. We compare the propagation of an optical pulse with three

different temporal pulse widths omitting the longitudinal and temporal fiber perturbations. Figure S2 shows the simulation results.

Figure S2(a) depicts the longitudinal evolution in the time domain of a 350-fs Gaussian optical pulse along the MCF length in the PCMs ax and bx. It can be noted that the initial pulse launched into the PCM ax splits in four identical optical pulses (25% of the initial peak power) propagated by the PCMs ax and bx of the fiber. These results are in line with Eq. (77) and the observations reported in [9]-[17]. Moreover, Fig. S2(b) shows the longitudinal evolution of the optical spectrum as a function of the normalized frequency. From these results, we conclude that each spectral component presents a different coupling length induced by the first-order CCD (as a direct consequence of the frequency dependence of the power confinement ratio of the LP_{01} mode in each core). Finally, we compare in Fig. S2(c) the pulse splitting in the PCM ax after the MCF propagation of three different Gaussian optical pulses of $T_P = 2$ ps, 350 fs and 200 fs. Note that the pulse splitting increases when the temporal pulse width is reduced, and remarkably, the complex envelope of the 200-fs optical pulse is additionally distorted by the second and higher-order CCD (inducing a linear and nonlinear chirp, respectively).

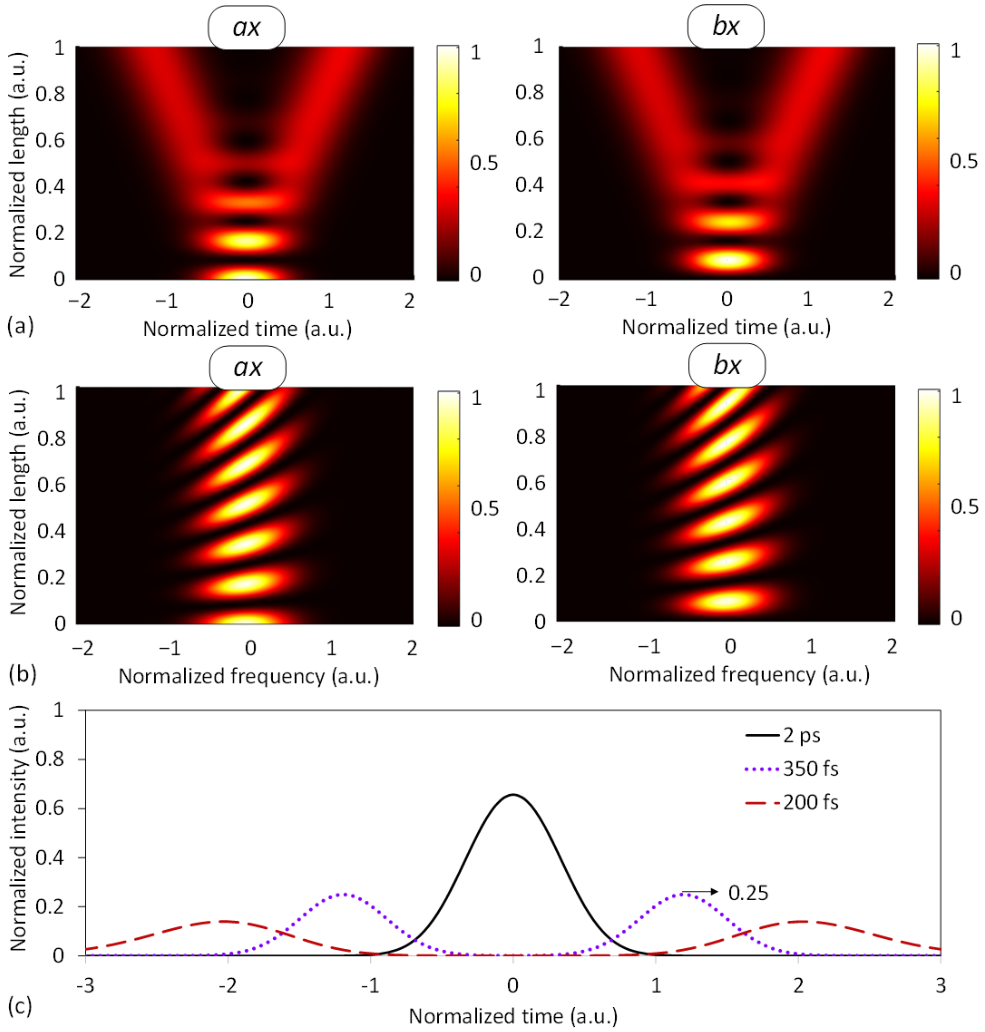

Supplementary Figure S2. Coupling-coefficient dispersion in ideal homogeneous MCFs. Simulation results of ultra-short optical pulses through a 40-m ideal homogeneous 2-core MCF. Longitudinal evolution of a 350-fs Gaussian optical pulse propagated through the polarized core modes (PCMs) ax and bx in: (a) time domain, and (b) frequency domain. (c) Pulse splitting comparison at the MCF output in the PCM ax considering three different pulse widths of 2 ps, 350 fs and 200 fs. (Colorbar: normalized intensity).

3.2 Linear and circular random birefringence perturbations: first-order IMCD

A very interesting example is to evaluate a real MCF perturbed by random linear and circular birefringence fluctuations. As discussed in the paper, we know that the random fiber perturbations induce a stochastic group delay in the optical pulse due to the first-order IMCD. In this case, the effect of the first-order PhMD along with the CCD can also be observed when considering a high number of MCF birefringent segments, where the bending radius and the twist rate fluctuate between adjacent segments with a Normal distribution of $R_{\mathrm{B}} = \mathrm{N}(\mu = 100, \sigma^2 = 40)$ cm and $f_{\mathrm{T}} = \mathrm{N}(\mu = 0.1, \sigma^2 = 0.01)$ turns/m. We simulate the homogeneous MCF of the first example considering a 250-fs Gaussian optical pulse and 50 birefringent segments.

It should be noted from Fig. S3(a) that the pulse splitting induced by the CCD is reduced in both PCMs as a direct consequence of the average value of $R_{\mathrm{B}} = 100$ cm reducing the mode-coupling between the cores a and b. Now, the group delay and the pulse width present a random evolution in each PCM due to the random nature of the MCF perturbations inducing a stochastic group velocity. Therefore, the effects of the IMCD when considering multiple random MCF segments can be observed as a distortion of the complex envelope. Moreover, the circular birefringence only induces power exchange between orthogonal polarizations. Remarkably, we cannot observe an additional pulse distortion induced by the PMD considering that the differential group delay between orthogonal polarizations can be neglected in both cores when the intrinsic linear birefringence is omitted in the numerical simulation. Here, the linear birefringence is only induced by the fiber bending conditions, which modify the equivalent refractive index of each PCM (see Section 4 for more details) without significantly changing the differential group delay between the PCMs ax-ay and bx-by in this case.

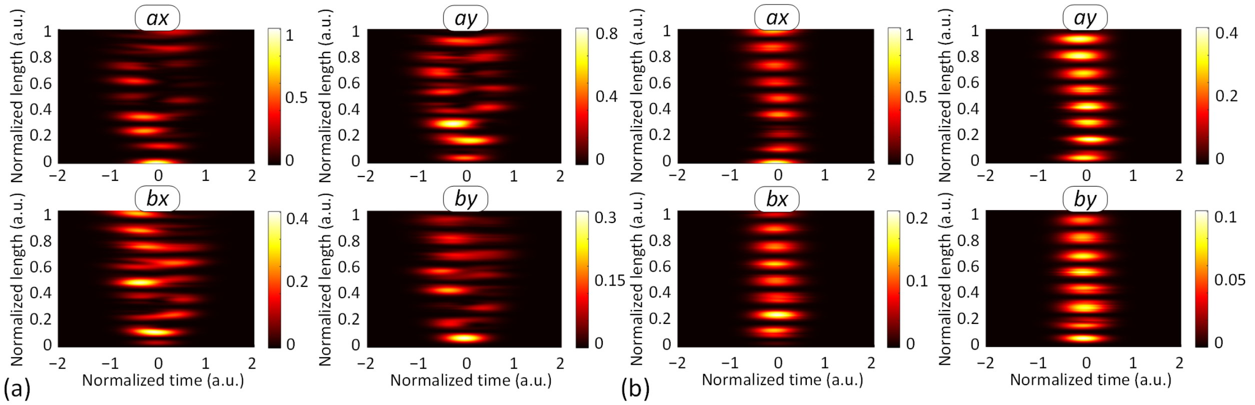

Supplementary Figure S3. First-order IMCD with random bending and twisting conditions. Simulation results of a 250-fs Gaussian optical pulse considering a homogeneous 2-core MCF with a random twist rate (f_{T}) and a random bending radius (R_{B}) which fluctuate along the MCF length comprising 50 birefringent segments with Normal distributions $f_{\mathrm{T}} = \mathrm{N}(\mu = 0.1, \sigma^2 = 0.01)$ turns/m and: (a) $R_{\mathrm{B}} = \mathrm{N}(\mu = 100, \sigma^2 = 40)$ cm, and (b) $R_{\mathrm{B}} = \mathrm{N}(\mu = 10, \sigma^2 = 2)$ cm. (Colorbar: normalized intensity).

In order to minimize the effects of the IMCD in homogeneous MCFs, we can reduce the average value of the bending radius. To verify this statement, we compare the propagation of the same 250-fs optical pulse considering a bending radius given by the Normal distribution $R_{\mathrm{B}} = \mathrm{N}(\mu = 10, \sigma^2 = 2)$ cm. We can note from Fig. S3(b) that the lower the average value of the bending radius, the lower is the pulse distortion induced by the IMCD as a direct consequence of the mode-coupling reduction between the PCMs. In general, we can also reduce the mode-coupling inducing external stress in different points along the MCF length or manufacturing MCFs with: trench- or hole-assisted cladding, heterogeneous cores, higher core-to-core distances or elliptical cores with random orientation of the ellipse. In the same way, the IMCD can also be reduced using disordered MCFs based on the transverse Anderson localization, allowing for the absence of diffusive wave propagation [24],[25].

3.3 Higher-order IMCD

In this section, we analyse the higher-order effects of the IMCD. We also include the intrinsic linear birefringence of the medium along with the linear and circular birefringence induced by the fiber bending and twisting conditions. To this end, two different 40-m 2-core MCFs are compared comprising homogeneous and heterogeneous cores. In the homogeneous case, higher-order PhMD effects are induced considering $\Delta\beta^{(1)}_{bx,ax} = 0.28$ ps/km, $\Delta\beta^{(2)}_{bx,ax} = 0.2$ ps^2/km, $\Delta\beta^{(3)}_{bx,ax} = 0$ ps^3/km; and in the heterogeneous case we assume $\Delta n = n_a - n_b = 0.002$, $\Delta\beta^{(1)}_{bx,ax} = 6.5$ ps/km, $\Delta\beta^{(2)}_{bx,ax} = 1$ ps^2/km and $\Delta\beta^{(3)}_{bx,ax} = 0.1$ ps^3/km. In order to illustrate the effects of the higher-order IMCD, the GVD is compensated in each PCM along the MCF propagation using the dispersive parameters of a given PCM as a reference, in this case the PCM ax (see Table S2 in Section 4 for more details). Furthermore, we also consider 50 birefringent segments along the MCF length, where the linear and circular birefringence fluctuate between adjacent segments. The circular birefringence is induced by a random twist rate f_{T} given by the Normal distribution $f_{\mathrm{T}} = \mathrm{N}(\mu = 0.1, \sigma^2 = 0.01)$ turns/m. The linear birefringence is induced by two different effects: (i) the random bending conditions with $R_{\mathrm{B}} = \mathrm{N}(\mu = 100, \sigma^2 = 40)$ cm; and (ii) the intrinsic linear birefringence of each core, fixed to $4{\cdot}10^{-7}$ and $2{\cdot}10^{-6}$ in the cores a and b, respectively. In this case, the intrinsic linear birefringence will induce a differential group delay between the principal axes of each birefringent segment.

Figures S4 and S5 depict the numerical calculations of the coupled local-mode equations when a 200-fs Gaussian optical pulse is launched into the PCM ax of the homogeneous and heterogeneous MCF, respectively. In the homogeneous case (Fig. S4), we can observe the additional chirp induced in the complex envelope of the optical pulse by the second-order PhMD along with the PMD. As expected, we can also observe a higher pulse distortion than omitting the PMD when comparing these results with Fig. 4(a) of the paper.

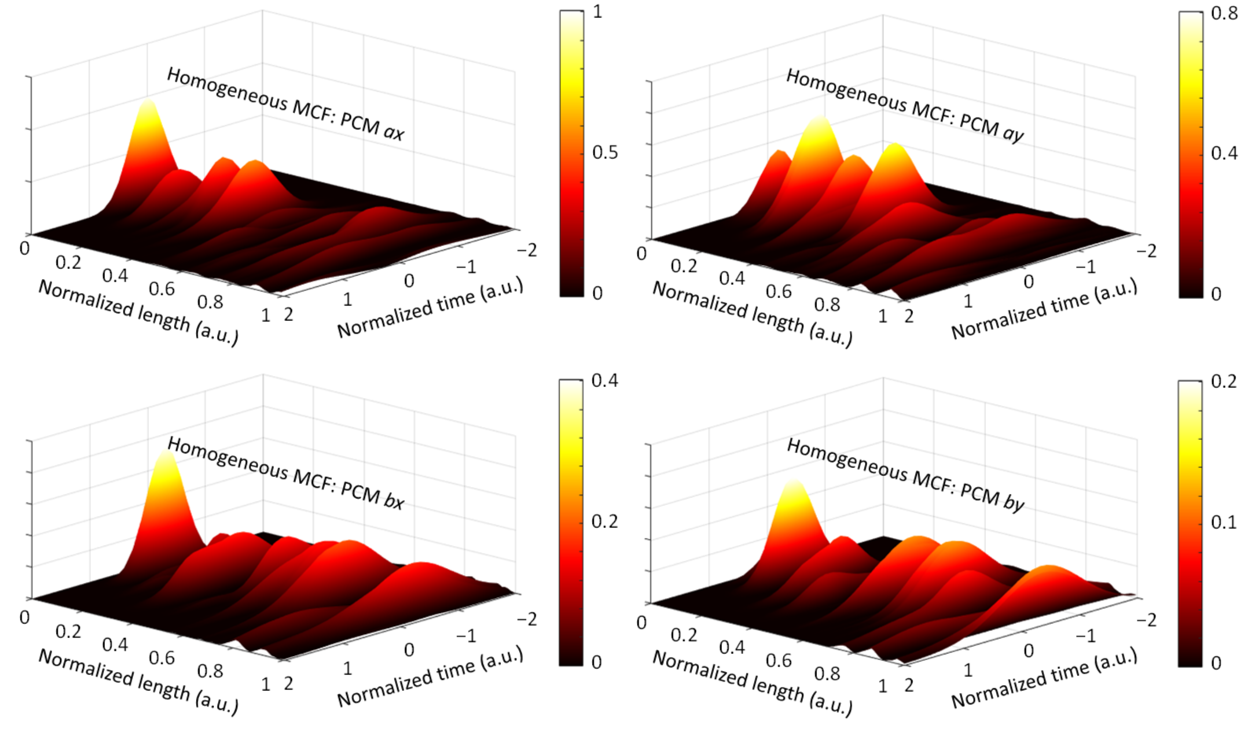

Supplementary Figure S4. Higher-order IMCD in homogeneous MCF. 200-fs Gaussian optical pulse propagated along a 40-m 2-core homogeneous MCF considering higher-order PhMD effects and a random linear and circular birefringence given by the bending radius and twist rate distributions $R_{\mathrm{B}} = \mathrm{N}(\mu = 100, \sigma^2 = 40)$ cm and $f_{\mathrm{T}} = \mathrm{N}(\mu = 0.1, \sigma^2 = 0.01)$ turns/m along 50 birefringent segments. (Colorbar: normalized intensity).

Nevertheless, as verified in Fig. S5, the pulse distortion induced by the second-order PhMD is reduced in the heterogeneous case given that the mode-coupling is minimized due to a higher intrinsic index-mismatching Δn between the cores a and b. As can be noted from Eq. (83), H_{ax} tends to 1 when the phase-mismatching between the cores a and b is much higher than the coupling coefficients, which verifies that the mode-coupling reduction allows us to minimize the effects of the IMCD in both cores. In the same way as in Fig. S4, note that the additional pulse distortion observed in Fig. S5 in the PCMs bx and by when comparing these results with Fig. 4(b) of the paper is induced by the PMD of the core b. Furthermore, it should be noticed that the optical intensity oscillates in the core a between the PCMs ax and ay because of the external circular birefringence induced by the twist rate.

On the other hand, in the homogeneous case, we can also induce an external perturbation (e.g. reducing the average value of the bending radius) to increase the equivalent index-mismatching between the PCMs reducing the higher-order effects of the IMCD. Additional strategies as trench-assisted MCFs or disordered MCFs based on the transverse Anderson localization [24],[25] can also be employed to reduce the pulse distortion induced by the first- and higher-order effects of the IMCD, as mentioned before.

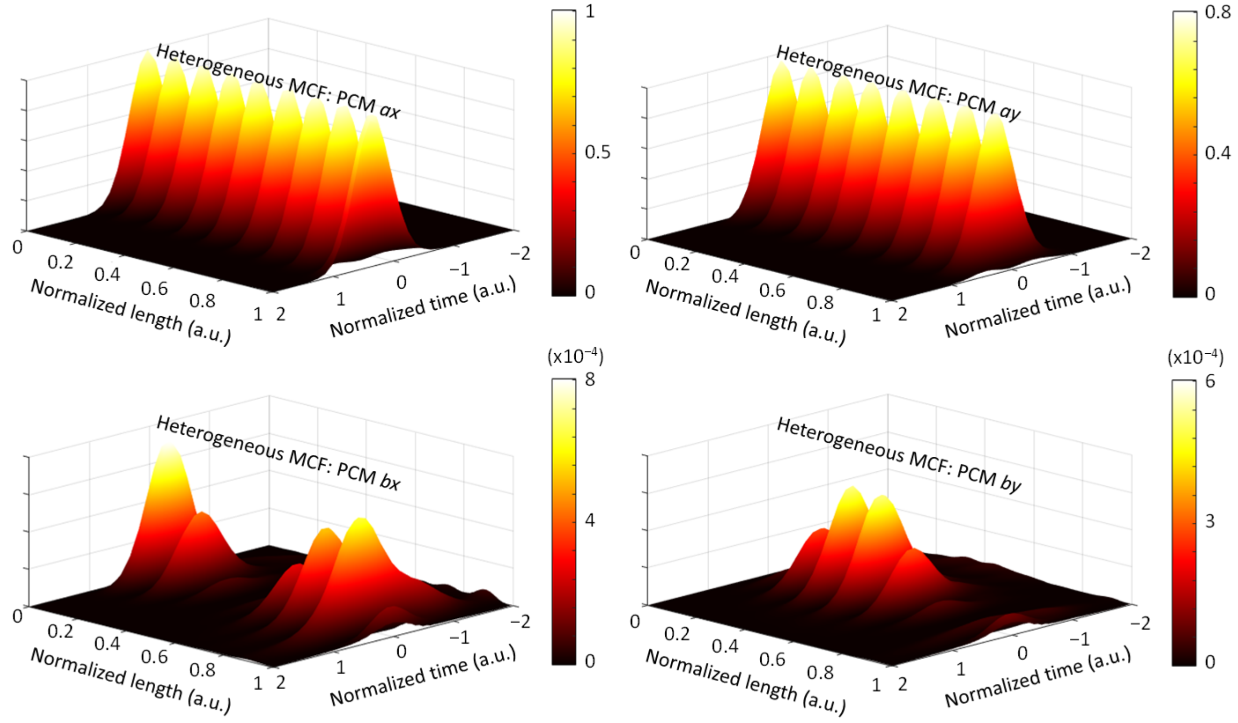

Supplementary Figure S5. Higher-order IMCD in heterogeneous MCF. 200-fs Gaussian optical pulse propagated along a 40-m 2-core heterogeneous MCF considering higher-order PhMD effects and a random linear and circular birefringence given by the bending radius and twist rate distributions $R_\mathrm{B} = \mathrm{N}(\mu = 100, \sigma^2 = 40)$ cm and $f_\mathrm{T} = \mathrm{N}(\mu = 0.1, \sigma^2 = 0.01)$ turns/m along 50 birefringent segments. (Colorbar: normalized intensity).

3.4 IMCD length

Finally, once we know in general terms the effects of the IMCD in ultra-short optical pulses, it is natural to ask about the fiber length scales over which the dispersive effects of the IMCD should be considered in the pulse propagation phenomena when comparing this optical dispersion with the first-order GVD. So far, we have discussed that the IMCD is reduced when inducing random perturbations in the medium. Therefore, the IMCD and the GVD should be compared in MCFs without including birefringence effects. To this end, we analyse the GVD and the IMCD lengths, defined as the fiber length scales over which their dispersive effects should be considered. The GVD length is defined in [7] as a function of the pulse half-width at $1/e$

of the peak power. Nonetheless, in this work we have investigated the IMCD using the full-width at $1/2e$ of the peak power (T_{P}). Thus, in order to compare the GVD with the IMCD, we define the GVD length as a function of T_{P}:

$$L_{\mathrm{GVD}} := T_{\mathrm{P}}^2 / \left| \beta_{ax}^{(2)} \right| . \tag{85}$$

In the same way, the CCD length is defined as the minimum fiber length over which the pulse splitting can be observed. Hence, considering the PCMs ax and bx, it is straightforward to conclude from Eq. (77) that the CCD length should be defined as:

$$L_{\mathrm{CCD}} := T_{\mathrm{P}} / 2 \left| \widetilde{k}_{ax,bx}^{(1)} \right| . \tag{86}$$

Furthermore, taking into account that the second-order PhMD induces a linear chirp between the PCMs ax and bx when $\Delta\beta_{bx,ax}^{(2)} \neq 0$, the PhMD length is defined in similar way as the GVD length:

$$L_{\mathrm{PhMD}} := T_{\mathrm{P}}^2 / \left| \Delta\beta_{bx,ax}^{(2)} \right| . \tag{87}$$

All in all, the IMCD length should be defined considering the predominant physical impairment, the CCD or the PhMD:

$$L_{\mathrm{IMCD}} := \min \left\{ L_{\mathrm{CCD}}, L_{\mathrm{PhMD}} \right\} . \tag{88}$$

4 Numerical method: local split-step Fourier method

The numerical simulations of the coupled local-mode equations have been performed in Matlab combining the equivalent refractive index model [1] and the split-step Fourier method [7] in each local birefringent segment. Both methods allow us to simulate the MCF perturbations and the linear and nonlinear propagation employing a low computational time. According to the split-step Fourier method, Eq. (65) should be rewritten as:

$$\left(\partial_z + \widehat{\mathrm{D}}_{ax}^{(\mathrm{eq})} + \frac{1}{2}\widehat{\alpha}\right) \mathcal{A}_{ax}(z,t) + j\widehat{\mathrm{M}}_{ax,ay}^{(\mathrm{eq})}\mathcal{A}_{ay}(z,t) + j\sum_{m=b}^{N} \widehat{\mathrm{K}}_{ax,mx}^{(\mathrm{eq})}\mathcal{A}_{mx}(z,t) = \widehat{\mathrm{N}}_{ax}^{(\mathrm{eq})}\mathcal{A}_{ax}(z,t)\,, \tag{89}$$

where $\widehat{\mathrm{N}}_{ax}^{(\mathrm{eq})}$ is the operator modelling the nonlinear propagation of the PCM ax:

$$\begin{aligned}
\widehat{\mathrm{N}}_{ax}^{(\mathrm{eq})} =& -j\widetilde{q}_{ax}^{(\mathrm{I})(0)}\left|\mathcal{A}_{ax}(z,t)\right|^2 - \frac{\widetilde{q}_{ax}^{(\mathrm{I})(1)}}{\mathcal{A}_{ax}(z,t)}\partial_t\left(\left|\mathcal{A}_{ax}(z,t)\right|^2\mathcal{A}_{ax}(z,t)\right)\\
&- j\frac{2}{3}\widetilde{g}_{ax,ay}^{(\mathrm{I})(0)}\left|\mathcal{A}_{ay}(z,t)\right|^2 - \frac{2}{3}\frac{\widetilde{g}_{ax,ay}^{(\mathrm{I})(1)}}{\mathcal{A}_{ax}(z,t)}\partial_t\left(\left|\mathcal{A}_{ay}(z,t)\right|^2\mathcal{A}_{ax}(z,t)\right)\\
&- \frac{1}{3}\frac{\exp\left(-j2\Delta\phi_{ay,ax}^{(0)}(z;t)\right)}{\mathcal{A}_{ax}(z,t)}\left[j\widetilde{g}_{ax,ay}^{(\mathrm{I})(0)}\mathcal{A}_{ax}^{*}(z,t)\,\mathcal{A}_{ay}^{2}(z,t) + \widetilde{g}_{ax,ay}^{(\mathrm{I})(1)}\partial_t\left(\mathcal{A}_{ax}^{*}(z,t)\,\mathcal{A}_{ay}^{2}(z,t)\right)\right]\\
&- j\widetilde{q}_{ax}^{(\mathrm{R})(0)}\left(f(t)*\left|\mathcal{A}_{ax}(z,t)\right|^2\right) - \frac{\widetilde{q}_{ax}^{(\mathrm{R})(1)}}{\mathcal{A}_{ax}(z,t)}\partial_t\left[\left(f(t)*\left|\mathcal{A}_{ax}(z,t)\right|^2\right)\mathcal{A}_{ax}(z,t)\right]\\
&- j\widetilde{g}_{ax,ay}^{(\mathrm{R})(0)}\left(h(t)*\left|\mathcal{A}_{ay}(z,t)\right|^2\right) - \frac{\widetilde{g}_{ax,ay}^{(\mathrm{R})(1)}}{\mathcal{A}_{ax}(z,t)}\partial_t\left[\left(h(t)*\left|\mathcal{A}_{ay}(z,t)\right|^2\right)\mathcal{A}_{ax}(z,t)\right]\\
&- j\frac{1}{2}\frac{\widetilde{g}_{ax,ay}^{(\mathrm{R})(0)}}{\mathcal{A}_{ax}(z,t)}\left\{\left[u(t)*\left(\mathcal{A}_{ax}(z,t)\,\mathcal{A}_{ay}^{*}(z,t)\right)\right]\mathcal{A}_{ay}(z,t)\right\}\\
&- \frac{1}{2}\frac{\widetilde{g}_{ax,ay}^{(\mathrm{R})(1)}}{\mathcal{A}_{ax}(z,t)}\partial_t\left\{\left[u(t)*\left(\mathcal{A}_{ax}(z,t)\,\mathcal{A}_{ay}^{*}(z,t)\right)\right]\mathcal{A}_{ay}(z,t)\right\}\\
&- j\frac{1}{2}\exp\left(-j2\Delta\phi_{ay,ax}^{(0)}(z;t)\right)\frac{\widetilde{g}_{ax,ay}^{(\mathrm{R})(0)}}{\mathcal{A}_{ax}(z,t)}\left\{\left[u(t)*\left(\mathcal{A}_{ax}^{*}(z,t)\,\mathcal{A}_{ay}(z,t)\right)\right]\mathcal{A}_{ay}(z,t)\right\}\\
&- \frac{1}{2}\exp\left(-j2\Delta\phi_{ay,ax}^{(0)}(z;t)\right)\frac{\widetilde{g}_{ax,ay}^{(\mathrm{R})(1)}}{\mathcal{A}_{ax}(z,t)}\partial_t\left\{\left[u(t)*\left(\mathcal{A}_{ax}^{*}(z,t)\,\mathcal{A}_{ay}(z,t)\right)\right]\mathcal{A}_{ay}(z,t)\right\}.
\end{aligned} \tag{90}$$

As can be seen in Fig. S6, the MCF length is assumed to be composed by different birefringent segments with a random orientation of the local principal axes. In addition, each birefringent segment is divided in several small fiber segments where the split-step Fourier method is applied. This method obtains an approximate solution of Eq. (89) by considering that in each small fiber segment, the linear and nonlinear propagation can be decoupled [7]. The LHS of Eq. (89), which describes the linear propagation and the linear mode-coupling, is simulated in the frequency domain, while the RHS, which accounts for the nonlinear propagation and the nonlinear mode-coupling, is simulated in the time domain. A further description of the split-step Fourier method combining both linear and nonlinear terms can be found in [7].

However, additional considerations of the coupling coefficients and real phase functions $\phi_{mi}(z,\omega;t)$ should be indicated to complete the description of the numerical model. In order to include the longitudinal and temporal MCF perturbations, the equivalent refractive index model of [1] is revisited to calculate the phase functions and the linear and nonlinear coupling coefficients in the frequency domain.

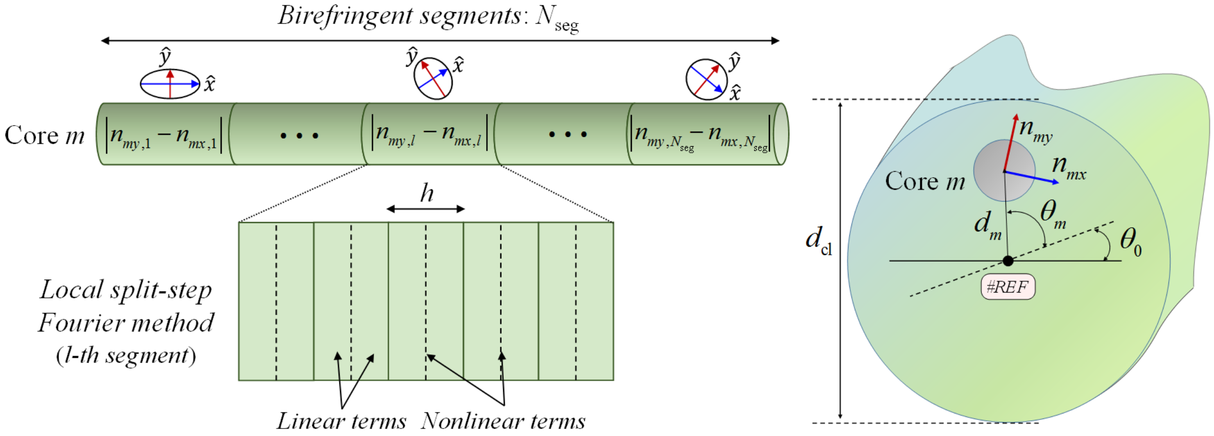

Supplementary Figure S6. Multi-core fiber simulation model. The MCF length is divided in different birefringent segments (N_{seg}) and local modes with a different time-varying retardation and random orientation of the local principal axes. Each birefringent segment is composed by small short distances (h) where the split-step Fourier method is applied.

Let us consider a short MCF distance h in a given l-th birefringent segment of the PCM mi, as depicted in Fig. S6. We assume to be constant along the l-th segment length: the bending radius $R_{\text{B},l}$, the twist rate $f_{\text{T},l}$ and the intrinsic linear birefringence $|n_{my,l}(t) - n_{mx,l}(t)|$. However, the intrinsic linear birefringence is found to be temporal dependent when simulating time-varying fluctuations in the optical medium (see Fig. 6 of the manuscript). Therefore, the phase function $\phi_{mi,l}$ can be written in each short MCF distance h as:

$$\phi_{mi,l}(z,\omega;t) = \int_{\langle h\rangle} \beta^{(\text{eq})}_{mi,l}(z,\omega;t)\,\mathrm{d}z \simeq \beta^{(\text{eq})}_{mi,l}(z,\omega;t)\,h = \left[\beta_{mi}(\omega) + \beta^{(\text{B+S})}_{mi,l}(z,\omega;t)\right]h. \tag{91}$$

The ideal phase constant $\beta_{mi}(\omega)$ can be approximated by a third-order Taylor series expansion as follows:

$$\beta_{mi}(\omega) \simeq \beta_{mi}(\omega_0) + \sum_{k=1}^{3}\frac{1}{k!}(\omega-\omega_0)^k\,\beta^{(k)}_{mi,\omega_0} \simeq \frac{\omega_0}{c_0}n_{mi} + \sum_{k=1}^{3}\frac{1}{k!}(\omega-\omega_0)^k\,\beta^{(k)}_{mi,\omega_0}, \tag{92}$$

with n_{mi} the refractive index of the PCM mi at the centre of the pulse bandwidth, which includes the intrinsic linear birefringence in highly-birefringent cores. In lowly-birefringent cores, n_{mi} is the material refractive index of the core m (n_m) at the centre of the pulse bandwidth. In addition, the longitudinal and temporal perturbations of the l-th segment are calculated using the equivalent refractive index model of [1] as follows:

$$\beta^{(\text{B+S})}_{mi,l}(z,\omega;t) \simeq \frac{\omega}{c_0}n^{(\text{B+S})}_{mi,l}(z,\omega_0;t) = \frac{\omega}{c_0}\left(n^{(\text{eq})}_{mi,l}(z,\omega_0;t) - n_{mi}\right); \tag{93}$$

$$n^{(\text{eq})}_{mi,l}(z,\omega_0;t) \simeq n_{mi,l}(t)\left[1 + \frac{d_m}{R_{\text{B},l}}\cos\left(2\pi f_{\text{T},l}z + \theta_0 + \theta_m\right)\right]. \tag{94}$$

In Eq. (94) $n_{mi,l}(t)$ describes the temporal birefringence fluctuation of the l-th birefringent segment, θ_0 is the offset of the twist angle of the MCF reference axis at $z = 0$; θ_m is the offset of the twist angle of the core m measured from the MCF reference axis; and d_m is the Euclidean distance of the core m to the MCF centre (see Fig. S6). The temporal birefringence fluctuation $|n_{my,l}(t) - n_{mx,l}(t)|$ is modelled by a Gaussian random process $\text{N}\left(\mu(t), \sigma^2\right)$. The mean $\mu(t) = \Delta n_m(t)$ is the value of the intrinsic linear birefringence of the core m at $\omega = \omega_0$, and it is assumed temporal dependent. The variance $\sigma^2 = \delta n_{m,l}$ includes the photo-elastic effect in the l-th segment [26]:

$$\delta n_{m,l} \simeq \xi + 0.011 n_m^3 d_{\rm cl}^2 / R_{{\rm B},l}^2, \tag{95}$$

where $\xi = 10^{-8}$, $d_{\rm cl}$ is the cladding diameter and n_m is the material refractive index of the core m at the centre of the pulse bandwidth. The parameter ξ is included in the equivalent refractive index model to describe the intrinsic longitudinal fluctuations of $|n_{my,l}(t) - n_{mx,l}(t)|$ between adjacent birefringent segments, according to usual manufacture imperfections in optical fibers [27]. Finally, the value $n_{mi,l}(t)$ is calculated as:

$$n_{mi,l}(t) \sim n_{mi} \pm \frac{1}{2} {\rm N}\left(\mu(t) = \Delta n_m(t), \sigma^2 = \delta n_{m,l}\right). \tag{96}$$

It is worth mentioning that Eq. (93) allows us to describe the random birefringence of the PCM mi. In contrast with the equivalent refractive index model reported in [1], Eq. (93) includes not only the birefringence induced by the bending and twisting conditions, but also the local random linear birefringence $n_{mi,l}(t) - n_{mi}$ induced by manufacturing imperfections in the l-th segment, which allows us to model the PMD between orthogonal PCMs of a given core m. On the other hand, although in the numerical simulations we have not considered temporal changes in the bending radius and twist rate, in a real deployed MCF system the environmental factors could induce temporal fluctuations in the bending radius and twist rate of the optical media. In such a case, $R_{{\rm B},l}$ and $f_{{\rm T},l}$ should be regarded as time-dependent variables.

Moreover, the coupling coefficients can be calculated numerically by using Eqs. (51)-(54). To this end, the transversal local eigenfunction $F_{mi,l}$ in the l-th segment can be calculated from the closed-form expressions detailed in [6] for the $\rm LP_{01}$ mode in the cores and cladding regions. Considering that the local eigenfunction depends on the longitudinal and temporal fibers perturbations, it should be calculated by using the equivalent refractive index model. Specifically, the eigenfunction $F_{mi,l}$ can be expressed in a local polar coordinate system (r, φ, z) of a given core m as:

$$F_{mi,l}(r, \omega; z, t) = \begin{cases} J_0\left(\frac{u_{mi,l}(z,\omega;t)}{R_{0,m}} r\right) / J_0\left(u_{mi,l}(z, \omega; t)\right); & r \leq R_{0,m} \\ K_0\left(\frac{w_{mi,l}(z,\omega;t)}{R_{0,m}} r\right) / K_0\left(w_{mi,l}(z, \omega; t)\right); & r > R_{0,m} \end{cases}, \tag{97}$$

where $R_{0,m}$ is the radius of the core m (assumed identical in each core in this work), and $u_{mi,l}$ and $w_{mi,l}$ are the modal parameters of the PCM mi:

$$u_{mi,l}(z, \omega; t) = \left(1 + \sqrt{2}\right) V_{mi,l}(z, \omega; t) / \left[1 + \left(4 + V_{mi,l}^4(z, \omega; t)\right)^{1/4}\right]; \tag{98}$$

$$w_{mi,l}(z, \omega; t) = \sqrt{V_{mi,l}^2(z, \omega; t) - u_{mi,l}^2(z, \omega; t)}. \tag{99}$$

An alternative and more accurate expression of $u_{mi,l}$ to estimate the crosstalk among PCMs can be found in [28] as $u_{mi,l} \simeq 2.405 \exp\left((0.5\delta_{mi,l} - 1)/V_{mi,l}\right)$, where $\delta_{mi,l} = ((n_{mi,l}^{\rm (eq)})^2 - n_{\rm cl}^2)/(n_{mi,l}^{\rm (eq)})^2$, $n_{\rm cl}$ is the cladding refractive index and $V_{mi,l}$ is the normalized frequency:

$$V_{mi,l}(z, \omega; t) = \frac{\omega}{c_0} R_{0,m} C_{{\rm F},m} \sqrt{\left(n_{mi,l}^{\rm (eq)}(z, \omega_0; t)\right)^2 - n_{\rm cl}^2}, \tag{100}$$

with $C_{{\rm F},m}$ an auxiliary parameter, referred to as the correction factor of the core m, included in Eq. (100) to calculate the correct value of the normalized frequency when using the nominal value of $R_{0,m}$. In real MCFs, the core radius presents longitudinal variations along the fiber length due to manufacturing imperfections. Hence, the average value of $R_{0,m}$ can be found to be different from the nominal value indicated in the MCF datasheet. In order to consider these core radius imperfections, the constant $C_{{\rm F},m}$ should be calculated by using the cut-off wavelength of each core ($\lambda_{{\rm C},m}$) as a reference:

$$C_{{\rm F},m} = \frac{2.405 \lambda_{{\rm C},m}}{2\pi R_{0,m} \sqrt{n_m^2 - n_{\rm cl}^2}}. \tag{101}$$

In our case, the correction factor is found to be $C_{\mathrm{F},m} \simeq 0.9$ when assuming $\lambda_{\mathrm{C},m} = 1410$ nm, $R_{0,m} = 4$ μm, $n_m = 1.452$ and $n_{\mathrm{cl}} = 1.444$ according to the datasheet of the MCF Fibercore SM-4C1500(8.0/125). Using Eqs. (97)-(101) it is straightforward to verify that $\partial_z F_{mi} \simeq \partial_z^2 F_{mi} \simeq 0$ in $\delta z \sim \lambda_0$ for usual values of f_{T} and R_{B} in real MCFs [1], in line with our initial assumptions in Eqs. (28) and (29).

In addition, in order to reduce the computational time of the numerical simulations in step-index MCFs, the coupling coefficients can also be calculated by using the closed-form expressions of [1] and [21] along with the equivalent refractive index model. Thus, the next expressions can be applied in each l-th birefringent segment:

$$\widetilde{m}_{ax,ay,l}\left(z,\omega;t\right) \simeq \frac{\pi}{2}\frac{\omega}{c_0}\left|p_{11}-p_{12}\right| f_{\mathrm{T},l} R_{0,a} \frac{n_a^4}{n_{ax,l}^{(\mathrm{eq})}\left(z,\omega_0;t\right)}; \tag{102}$$

$$\widetilde{k}_{ax,bx,l}\left(z,\omega;t\right) \simeq 2\frac{\omega}{c_0}\left(n_{ax,l}^{(\mathrm{eq})}\left(z,\omega_0;t\right) - n_{\mathrm{cl}}\right)\frac{J_0\left(u_{ax,l}\right)J_1\left(u_{ax,l}\right)}{u_{ax,l}\left[J_0^2\left(u_{ax,l}\right)+J_1^2\left(u_{ax,l}\right)\right]}\frac{K_0\left(w_{bx,l}d_{ab}/R_{0,b}\right)}{K_0\left(w_{bx,l}\right)}; \tag{103}$$

$$\widetilde{q}_{ax,l}^{(\mathrm{S})}\left(z,\omega;t\right) \simeq \widetilde{g}_{ax,ay}^{(\mathrm{S})}\left(z,\omega;t\right) \simeq \frac{1}{8}\frac{\omega}{c_0}\frac{\gamma_{\mathrm{S}}\left(\omega\right)}{n_{ax,l}^{(\mathrm{eq})}\left(z,\omega_0;t\right)}\frac{H_{ax,l}^2}{R_{0,a}^2}\frac{\left[1-\exp\left(-4R_{0,a}^2/H_{ax,l}^2\right)\right]}{J_0^2\left(u_{ax,l}\right)\left[J_0^2\left(u_{ax,l}\right)+J_1^2\left(u_{ax,l}\right)\right]}, \tag{104}$$

where p_{11} and p_{12} are components of the photo-elastic tensor ($p_{11} - p_{12} = 0.149$) [22], n_a is the material refractive index of the core a, $H_{ax,l}$ is the modal field radius of the PCM ax in the l-th segment given by the expression $H_{ax,l} \simeq R_{0,a}(0.65 + 1.619V_{ax,l}^{-3/2} + 2.879V_{ax,l}^{-6})$ [7], and $\gamma_{\mathrm{I}}\left(\omega\right)$ and $\gamma_{\mathrm{R}}\left(\omega\right)$ are the nonlinear parameters defined in Section 1.3. Considering the low frequency dependence of the nonlinear nature of silica fibers [22], the nonlinear fiber regime is simulated in the time domain using the operator $\widehat{\mathrm{N}}_{ax}^{(\mathrm{eq})}$ assuming a first-order Taylor series approximation in the nonlinear coupling coefficients. Moreover, note that the Kerr effect could also be modelled in Eq. (93). Nevertheless, this approach could increase the computational time of the coupled local-mode equations, since the nonlinear changes of the refractive index depend on the power of the optical pulses. Hence, it is a less time-consuming approach to model the nonlinear effects only by the nonlinear coupling coefficients.

An additional fundamental remark should be made from Fig. S6. As mentioned before, the local orientation of the fiber principal axes is assumed to be different between adjacent segments. In particular, this is modelled through the longitudinal dependence of the first-order susceptibility $\widetilde{\boldsymbol{\chi}}^{(1)}$. The diagonal terms are modified when varying $n_{mi}^{(\mathrm{eq})}$ by using Eq. (94), and the off-diagonal terms are given by Eq. (20), which is calculated using Eq. (13) of [1]. As a result, the local eigenstate of each birefringent segment is changed as a consequence of the longitudinal fluctuations of the twist rate. For short MCF distances, a low variance of the f_{T} distribution seems to be sufficiently accurate to describe the MCF birefringence, as was experimentally demonstrated in [1]. Nonetheless, when large MCF distances of several kilometres are involved, the orientation of the principal axes should be adequately randomized by increasing the variance of the f_{T} distribution, or combining this approach with additional strategies such as the phase plates [29]. In this fashion, the computational time may also be reduced by inserting phase plates between birefringent segments and reducing the variance of the f_{T} distribution.

Finally, we include in Tables S1 and S2 the main MCF parameters employed in the numerical calculations. Additional fiber parameters employed in the numerical simulations are listed below[6]:

- Fiber length: $L = 40$ m
- Optical carrier wavelength: $\lambda_0 = 1550$ nm
- Cut-off wavelength of cores a and b (*): $\lambda_{\mathrm{C},a} = \lambda_{\mathrm{C},b} = 1410$ nm
- Core radius of cores a and b (*): $R_{0,a} = R_{0,b} = 4$ μm

[6]The symbol (*) indicates the fiber parameters retrieved from the Fibercore SM-4C1500(8.0/125) datasheet.

- Core-to-core distance: $d_{ab} = 26$ μm
- Distance of core a and b to the MCF centre: $d_a = d_b = d_{ab}/\sqrt{2}$
- Cladding diameter (*): $d_{\text{cl}} = 125$ μm
- Cladding refractive index (*): $n_{\text{cl}} = 1.444$
- Nonlinear refractive index: $n_{\text{NL}}(\omega) \simeq n_{\text{NL}}^{(0)} + (\omega - \omega_0)\, n_{\text{NL}}^{(1)}$, with $n_{\text{NL}}^{(0)} = 2.6 \cdot 10^{-20}$ m^2/W and $n_{\text{NL}}^{(1)} = 8.3 \cdot 10^{-24}$ ps$\cdot$m^2/W
- Nonlinear parameters: $\gamma_{\text{I}}(\omega) \simeq \gamma_{\text{I}}^{(0)} + (\omega - \omega_0)\, \gamma_{\text{I}}^{(1)}$ and $\gamma_{\text{R}}(\omega) \simeq \gamma_{\text{R}}^{(0)} + (\omega - \omega_0)\, \gamma_{\text{R}}^{(1)}$; with $\gamma_{\text{I}}^{(0)} = 7.5 \cdot 10^{-20}$ m^2/W, $\gamma_{\text{I}}^{(1)} = 2.4 \cdot 10^{-23}$ ps$\cdot$m^2/W, $\gamma_{\text{R}}^{(0)} = 1.6 \cdot 10^{-20}$ m^2/W, and $\gamma_{\text{R}}^{(1)} = 5.2 \cdot 10^{-24}$ ps$\cdot$m^2/W
- Attenuation coefficient (*): $\alpha^{(0)} = 0.44$ dB/km $= 0.051$ Np/km, $\alpha^{(1)} = 3.7 \cdot 10^{-4}$ ps$\cdot$Np/km
- Peak Power: P_0
- Pulse width (full-width at $1/2e$ of the peak power): $T_{\text{P}} \simeq 1.56 \cdot T_{\text{FWHM}}$ for Gaussian pulses and $T_{\text{P}} \simeq 1.7 \cdot T_{\text{FWHM}}$ for Hyperbolic-Secant pulses (FWHM: Full Width at Half Maximum)
- Material refractive index of cores a and b: n_a, n_b
- Intrinsic linear birefringence in the l-th birefringent segment: $|n_{my,l}(t) - n_{mx,l}(t)| \sim \text{N}(\Delta n_m(t), \delta n_{m,l})$. Mean included in Table S1. Variance given by Eq. (95).
- Number of birefringent segments: N_{seg}
- Number of small fiber segments in each birefringent segment: N_{small}
- Bending radius: R_{B}
- Twist rate: f_{T}

Simulation	T_P	P_0	Pulse	n_a	n_b	$\Delta n_a(t)$	$\Delta n_b(t)$	N_seg	N_small	R_B	f_T
	(fs)	(dBm)								(cm)	(turns/m)
Fig. 2(a)	350	0	Gaussian	1.452	1.452	0	0	1	200	∞	0
Fig. 2(b)	350	0	Gaussian	1.452	1.452	0	0	1	200	10	0
Fig. 2(c)	350	0	Gaussian	1.452	1.452	0	0	1	200	1	0
Fig. 3(a)	250	0	Gaussian	1.452	1.452	0	0	50	10	N(100,40)	0
Fig. 3(b)	250	0	Gaussian	1.452	1.452	0	0	50	10	N(10,2)	0
Fig. 4(a)	200	0	Gaussian	1.452	1.452	0	0	50	10	N(100,40)	0
Fig. 4(b)	200	0	Gaussian	1.454	1.452	0	0	50	10	N(100,40)	0
Fig. 5	600	40.7	Sech	1.452	1.452	$2{\cdot}10^{-7}$	$2{\cdot}10^{-7}$	50	10	N(100,40)	N(0.1,0.01)
Fig. 6	150	0	Gaussian	1.452	1.452	Fig. 6(a)	Fig. 6(a)	50	10	100	0
Fig. S2	350	0	Gaussian	1.452	1.452	0	0	1	200	∞	0
Fig. S3(a)	250	0	Gaussian	1.452	1.452	0	0	50	10	N(100,40)	N(0.1,0.01)
Fig. S3(b)	250	0	Gaussian	1.452	1.452	0	0	50	10	N(10,2)	N(0.1,0.01)
Fig. S4	200	0	Gaussian	1.452	1.452	$4{\cdot}10^{-7}$	$2{\cdot}10^{-6}$	50	10	N(100,40)	N(0.1,0.01)
Fig. S5	200	0	Gaussian	1.454	1.452	$4{\cdot}10^{-7}$	$2{\cdot}10^{-6}$	50	10	N(100,40)	N(0.1,0.01)

Supplementary Table S1. Simulation parameters employed in the numerical calculations. $\mathrm{N}(\mu, \sigma^2)$ is the Normal distribution of mean μ and variance σ^2.

Simulation	GVD	GVD$_\mathrm{comp}$	$\Delta\beta^{(1)}_{bx,ax}$	$\beta^{(2)}_{ax}$	$\beta^{(3)}_{ax}$	$\beta^{(2)}_{bx}$	$\beta^{(3)}_{bx}$
			(ps/km)	(ps^2/km)	(ps^3/km)	(ps^2/km)	(ps^3/km)
Fig. 2(a)	OFF	–	0	−21.75	0.16	−21.75	0.16
Fig. 2(b)	OFF	–	0	−21.75	0.16	−21.75	0.16
Fig. 2(c)	OFF	–	0	−21.75	0.16	−21.75	0.16
Fig. 3(a)	OFF	–	0	−21.75	0.16	−21.75	0.16
Fig. 3(b)	OFF	–	0	−21.75	0.16	−21.75	0.16
Fig. 4(a)	ON	ON	0.28	−21.75	0.16	−21.95	0.16
Fig. 4(b)	ON	ON	6.5	−21.75	0.16	−22.75	0.26
Fig. 5	ON	OFF	0.2	−1	0.1	−1.1	0.1
Fig. 6	ON	ON	0.28	−21.75	0.16	−21.95	0.16
Fig. S2	OFF	–	0	−21.75	0.16	−21.75	0.16
Fig. S3(a)	OFF	–	0	−21.75	0.16	−21.75	0.16
Fig. S3(b)	OFF	–	0	−21.75	0.16	−21.75	0.16
Fig. S4	ON	ON	0.28	−21.75	0.16	−21.95	0.16
Fig. S5	ON	ON	6.5	−21.75	0.16	−22.75	0.26

Supplementary Table S2. Dispersive parameters employed in the numerical calculations. GVD: ON/OFF indicates whether the chromatic dispersion is included in the simulation (ON) or not (OFF). GVD$_\mathrm{comp}$: ON/OFF indicates whether the chromatic dispersion is compensated (ON) or not (OFF) along the MCF propagation in each PCM using the GVD parameters of the PCM ax as a reference. That is, assuming $\beta^{(r)}_{ax} = 0$ and $\beta^{(r)}_{bx} \equiv \Delta\beta^{(r)}_{bx,ax}$ with $r \in \{2, 3\}$. Dispersive parameters are assumed similar for the y-polarization (lowly-birefringent cores). The time domain is normalized using $\beta^{(1)}_{ax}$ as a reference. Hence, the relative group delay between the PCMs bx and ax only depends on $\Delta\beta^{(1)}_{bx,ax}$ but not on $\beta^{(1)}_{ax}$, i.e., $\beta^{(1)}_{ax}$ is just a gauge variable in the numerical simulations.

References

[1] Macho, A., Meca, C. G., Fraile-Peláez, F. J., Morant, M. & Llorente, R. Birefringence effects in multi-core fiber: coupled local-mode theory. *Opt. Express* **24**(19), 21415–21434 (2016).

[2] Saleh, B. E. A. & Teich, M. C. *Fundamentals of Photonics* (Wiley, 2007).

[3] Weiner, A. M. *Ultrafast Optics* (John Wiley & Sons, 2009).

[4] Boyd, R. W. *Nonlinear Optics* (Elsevier, 2008).

[5] Marcuse, D. *Theory of Dielectric Optical Waveguides* (Elsevier, 1974).

[6] Gloge, D. Weakly guiding fibers. *Applied Optics* **10**(10), 2252–2258 (1971).

[7] Agrawal, G. P. *Nonlinear Fiber Optics* (Elsevier, 2013).

[8] Mamyshev P. V. & Chernikov, S. V. Ultrashort-pulse propagation in optical fibers. *Opt. Lett.* **15**(19), 1076–1078 (1990).

[9] Chiang, K. S. Coupled-mode equations for pulse switching in parallel waveguides. *IEEE J. Quant. Electron.* **33**(6), 950–954 (1997).

[10] Someda, C. G. & Cauduro, F. Propagation in a decoupled twin-core waveguide: a frequency-domain analysis. *IEEE Phot. Journal* **4**(2), 422–426 (2012).

[11] Liu, M. & Chiang, K. S. Pulse propagation in a decoupled two-core fiber. *Opt. Express* **18**(20), 21261–21268 (2010).

[12] Kartashov, Y. V., Konotop, V. V. & Malomed, B. A. Dark solitons in dual-core waveguides with dispersive coupling. *Opt. Lett.* **40**(17), 4126–4129 (2015).

[13] Kartashov, Y. V., Malomed, B. A., Konotop, V. V., Lobanov, V. E. & Torner, L. Stabilization of spatiotemporal solitons in Kerr media by dispersive coupling. *Opt. Lett.* **40**(6), 1045–1048 (2015).

[14] Li, J. H., Chiang, K. S. & Chow, K. W. Suppression of pulse splitting in two-core optical fibers with Kerr nonlinearity. *J. Opt. Soc. Am. B* **30**(2), 460–467 (2013).

[15] Liu, M. & Chiang, K. S. Effects of intrapulse stimulated Raman scattering on short pulse propagation in a nonlinear two-core fiber. *Applied Physics B* **87**, 45–52 (2007).

[16] Li, Q., Xie, Y., Zhu, Y. & Zhao, Z. Effects of second-order coupling coefficient dispersion on short-pulse propagation and switching in an active two-core nonlinear fiber coupler. *J. Lightwave Technol.* **27**(15), 2933–2940 (2009).

[17] Liu, M., Chiang. K. S. & Shum, P. Evaluation of intermodal dispersion in a two-core fiber with non-identical cores. *Opt. Commun.* **219**, 171–176 (2003).

[18] Jackson, J. D. *Classical Electrodynamics* (Wiley & Sons, 1998).

[19] Huard, S. *Polarization of Light* (John Wiley & Sons, 1997).

[20] Lin, Q. & Agrawal, G. Raman response function for silica fibers. *Opt. Lett.* **31**(21), 3086–3088 (2006).

[21] Macho, A., Morant, M. & Llorente, R. Unified model of linear and nonlinear crosstalk in multi-core fiber. *J. Lightwave Technol.* **34**(13), 3035–3046 (2016).

[22] Weber, M. J. *Handbook of Optical Materials* (CRC Press, 2003).

[23] Wai, P. K. A., Kath, W. I., Menyuk, C. R. & Zhang, J. W. Nonlinear polarization-mode dispersion in optical fibers with randomly varying birefringence. *J. Opt. Soc. Am. B.* **24**(11), 2967–2979 (1997).

[24] Scwartz, T., Bartal, G., Fishman, S. & Segev, M. Transport and Anderson localization in disordered two-dimensional photonic lattices. *Nature* **446**, 52–55 (2007).

[25] Karbasi, S. *et al.* Image transport through a disordered optical fibre mediated by transverse Anderson localization. *Nature Commun.* **5**, 3362 (2014).

[26] Iizuka, K. *Elements of Photonics Volume I* (Wiley-Interscience, 2002).

[27] Brodsky, M., Frigo, N. J., & Tur, M. Polarization mode dispersion. In *Optical Fiber Telecommunications V A: Components and Subsystems* (eds. Kaminow, I., Li, T., Willner, A. E.) 605-669 (Elsevier, 2008).

[28] Snyder, A. Coupled-mode theory for optical fibers. *J. Opt. Soc. Am.* **62**, 1267—1277 (1972).

[29] Mumtaz, S., Essiambre, R. J. & Agrawal, G. P. Nonlinear propagation in multimode and multicore fibers: generalization of the Manakov equations. *J. Lightwave Technol.* **31**(3), 398–406 (2013).
